# Synthesis and Assessment of Antiplatelet and Antithrombotic Activity of 4-Amino-Substituted 5-Oxoproline Amides and Peptides

**DOI:** 10.3390/molecules28217401

**Published:** 2023-11-02

**Authors:** Victor P. Krasnov, Irina A. Nizova, Alexey Yu. Vigorov, Tatyana V. Matveeva, Galina L. Levit, Mikhail I. Kodess, Marina A. Ezhikova, Pavel A. Slepukhin, Dmitry A. Bakulin, Ivan N. Tyurenkov, Valery N. Charushin

**Affiliations:** 1 Postovsky Institute of Organic Synthesis, Russian Academy of Sciences (Ural Branch), Ekaterinburg 620108, Russia; nizova@ios.uran.ru (I.A.N.); vigorov@ios.uran.ru (A.Y.V.); matveeva@ios.uran.ru (T.V.M.); ca512@ios.uran.ru (G.L.L.); nmr@ios.uran.ru (M.I.K.); ema@ios.uran.ru (M.A.E.); slepukin@ios.uran.ru (P.A.S.); charushin@ios.uran.ru (V.N.C.); 2 Laboratory of Pharmacology of Cardiovascular Agents, Scientific Center for Innovative Medicines, Volgograd State Medical University, Volgograd 400131, Russia; mbfdoc@gmail.com (D.A.B.); fibfuv@mail.ru (I.N.T.); 3 Chemical Engineering Institute, Ural Federal University, Ekaterinburg 620002, Russia

**Keywords:** 5-oxoproline, pyroglutamic acid, piperidine, morpholine, phenylalanine, histidine, amides, peptides, in vivo experiments

## Abstract

Venous thromboembolism is a serious problem because it significantly increases the risk of developing vascular complications in elderly patients with obesity or immobilization, cancer, and many other diseases. Thus, there is a need to study new therapeutic strategies, including new medicinal agents for the efficient and safe correction of thrombus disorders. In this work, we have synthesized a number of new amides and peptides of 4-amino-5-oxoprolines and studied their antiplatelet and antithrombotic activity in experiments in vitro and in vivo. It has been found that the newly obtained compounds slow down the process of thrombus formation in a model of arterial and venous thrombosis, without affecting plasma hemostasis parameters. (2*S*,4*S*)-4-Amino-1-(4-fluorophenyl)-5-oxoprolyl-(*S*)-phenylalanine proved to be the most efficient among the studied derivatives. The results obtained indicate the advisability of further studies on 5-oxoproline derivatives in order to design pharmaceutical agents for the prevention and treatment of the consequences of thrombosis.

## 1. Introduction

Thromboembolic complications remain a serious medical problem, representing one of the leading causes of mortality in cardiology, oncology, pulmonology, and traumatology patients. Among these complications, pulmonary embolism (PE) stands out as a particularly dangerous condition, for which postoperative mortality is 10–20% [1,2,3]. In addition, PE is the third most common cardiovascular disease with a significant public health burden, affecting 250–300 thousand people annually in Europe and in the United States [4,5].

Moreover, venous thromboembolism (VTE) is emerging as another major issue, ranking third among all cardiovascular diseases, second only to myocardial infarction and ischemic stroke. The significance of VTE goes beyond its direct consequences, since VTE significantly increases the risk of developing vascular complications in elderly patients with obesity or immobilization, cancer, and many other diseases [6,7,8].

Thus, there is a need to study new therapeutic strategies, including new medicinal agents, for the efficient and safe correction of thrombus disorders [9,10].

**Figure 1:**
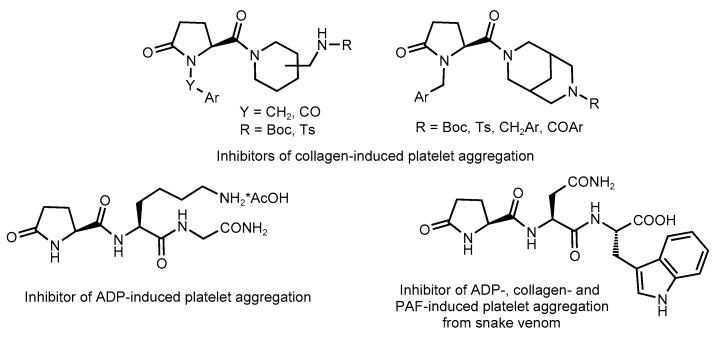
Selected literature examples of 5-oxoproline derivatives with antiplatelet activity. Amides of *N*-substituted 5-oxoprolines as inhibitors of collagen-induced platelet aggregation [11,12] and short peptides with *N*-terminal 5-oxoproline as inhibitors of platelet aggregation [13,14].

It is known that, among the derivatives of 5-oxoproline (or pyroglutamic acid, Glp), there are compounds exhibiting antiplatelet activity, slowing down collagen-induced platelet aggregation in vitro and in vivo [11,12]. Short peptides containing *N*-terminal 5-oxoproline have also been reported to exhibit antiplatelet activity: for example, Glp-Lys-GlyNH_2_ inhibits ADP-induced platelet aggregation in vitro and in vivo [13], and Glp-Asn-Trp inhibits platelet aggregation induced by ADP, collagen, and the platelet activating factor in vitro [14] (Figure 1).

**Figure 2:**
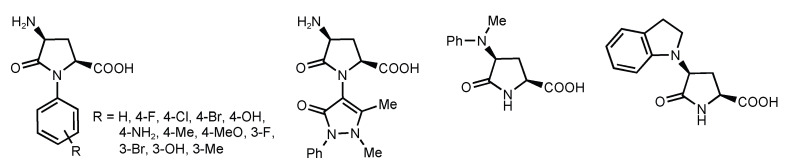
4-Amino-5-oxoprolines as platelet aggregation inhibitors studied in previous works [15,16].

We have previously shown that 4-amino-1-aryl-5-oxoprolines and 4-(arylamino)-5-oxoprolines exhibit antiplatelet and antithrombotic activity in vitro and in vivo (Figure 2); the best results in this type of activity were shown by (2*S*,4*S*)-4-amino-1-(4-fluorophenyl)-5-oxoproline [15,16]. Some derivatives of this series also demonstrated cerebroprotective and psychotropic activity in experiments in vivo [16,17].

In this work, we synthesized a number of new amides and peptides of 4-amino-substituted 5-oxoprolines and tested their antiplatelet and antithrombotic activity in both in vitro and in vivo experiments.

## 2. Results and Discussion

### 2.1. Chemistry

**Scheme 1:**
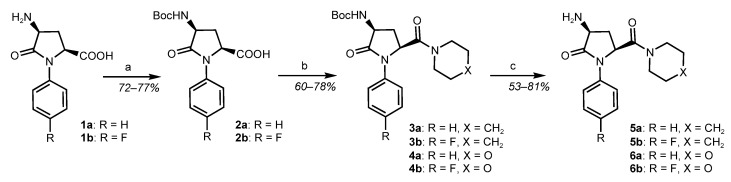
Synthesis of amides **5a,b** and **6a,b**. (a) Boc_2_O, K_2_CO_3_, H_2_O–*i*PrOH, 35–40 °C, 2 h; (b) piperidine (for **3a,b**) or morpholine (for **4a,b**), EDC × HCl, HOBt, DIEA, MeCN, rt, 1–3 days; and (c) 1. CF_3_CO_2_H, CH_2_Cl_2_, rt, 2 h; 2. 1 N NaOH, H_2_O (pH = 9), rt, 30 min.

(2*S*,4*S*)-4-Amino-1-phenyl-5-oxoproline (**1a**) [18] and (2*S*,4*S*)-4-amino-1-(4-fluorophenyl)-5-oxoproline (**1b**) [15] were chosen as the starting derivatives of 4-amino-5-oxoprolines (Scheme 1). During the first stage, the 4-amino group of 5-oxoprolines **1a,b** was protected by introducing a *tert*-butoxycarbonyl (Boc) group through the action of Boc_2_O to afford acids **2a,b** in high yields. The coupling reaction with piperidine or morpholine was carried out using EDC × HCl (1-ethyl-3-(3-dimethylaminopropyl)carbodiimide hydrochloride) as a coupling agent in the presence of HOBt·(1-hydroxybenzotriazole hydrate) and DIEA (*N*,*N*-diisopropylethylamine). In the next stage, the Boc group was removed through the action of trifluoroacetic acid (TFA) in CH_2_Cl_2_ and the target compounds **5a,b** and **6a,b** were isolated in moderate to high yields using alkalization with aqueous NaOH.

**Scheme 2:**
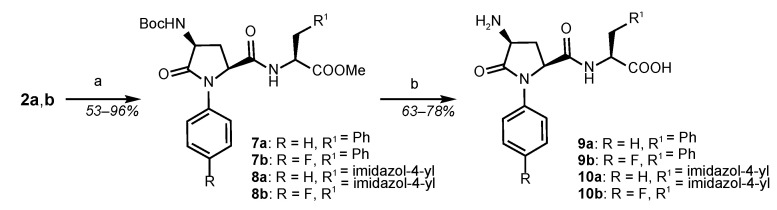
Synthesis of dipeptides **9a,b** and **10a,b**. (a) PheOMe × HCl, HBTU (for **7a,b**) or HisOMe × 2HCl, TBTU (for **8a,b**), DIEA, MeCN, rt, 2–3 days; and (b) 1. CF_3_CO_2_H, CH_2_Cl_2_, rt, 2 h; 2. 1 *N* NaOH (3 eq.), rt, 4 h; additional treatment with Amberlite^®^ IR-120 (H^+^) for **10a,b**.

For the synthesis of dipeptides, acids **2a,b** were coupled to methyl (*S*)-phenylalaninate or (*S*)-histidinate under the action of HBTU (hexafluorophosphate benzotriazole tetramethyl uranium) or TBTU (2-(1*H*-benzotriazole-1-yl)-1,1,3,3-tetramethylaminium tetrafluoroborate) as coupling agents to obtain compounds **7a,b** or **8a,b**, respectively (Scheme 2). The protecting groups in peptides **7–8a,b** were removed using a sequential treatment with TFA and aqueous NaOH in order to form the corresponding peptides **9–10a,b**. **9a,b** precipitated from an aqueous alkali solution upon acidification with 1*M* HCl to pH 3–4; the dipeptides **10a,b** were isolated on an ion-exchange resin (Scheme 2).

**Scheme 3:**
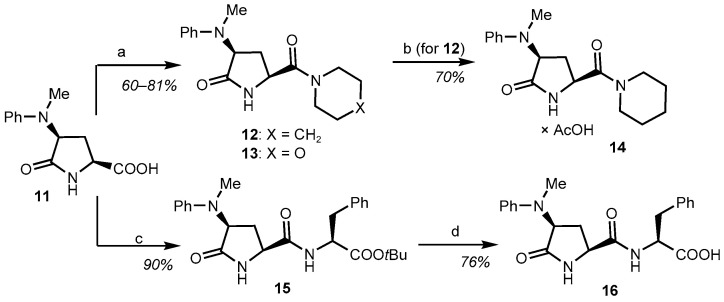
Synthesis of compounds **12**, **13**, and **16**. (a) Piperidine (for **12**) or morpholine (for **13**), HBTU, DMF, rt, 1 day; (b) AcOH, *n*-hexane–EtOAc 10:1, −10 °C, 3 days; (c) PheO*t*Bu, HBTU, DIEA, DMF, rt, 1 day; and (d) CF_3_CO_2_H, rt, 2 h.

Starting from the previously described (2*S*,4*S*)-4-[(methyl)(phenyl)amino]-5-oxoproline (**11**) [19], we synthesized amides with piperidine (**12**) and morpholine (**13**) (Scheme 3). The coupling to the amino component (piperidine or morpholine) was carried out under the action of HBTU in DMF. **12** and **13** are fine crystals (powders). The crystallization of compound **12** from a 10:1 *n*-hexane–ethyl acetate mixture in the presence of one equiv. AcOH made it possible to obtain single crystals of compound **14** in the form of a solvate with AcOH, which were suitable for an X-ray diffraction analysis. The coupling of 5-oxoproline **11** with *tert*-butyl (*S*)-phenylalaninate under the action of HBTU in DMF, followed by cleavage of the *tert*-butyl ester with TFA in compound **15** yielded dipeptide **16** (Scheme 3).

**Figure 3:**
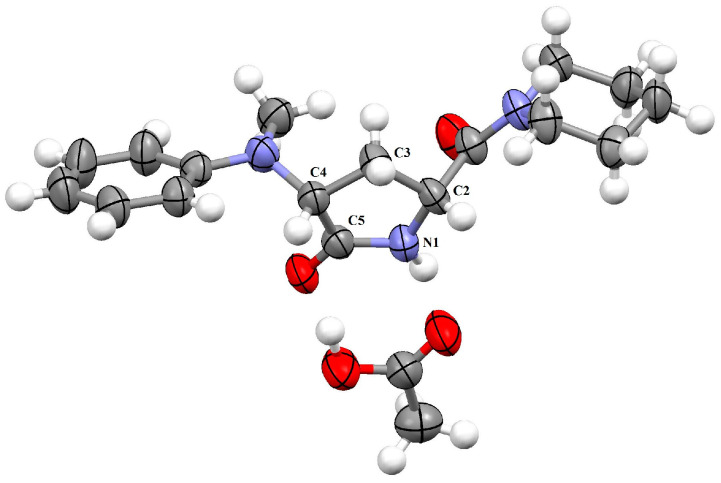
ORTEP structure of compound **14** (thermal ellipsoids of 50% probability).

According to the XRD data, solvate **14** is crystallized in the non-centrosymmetrical space group as a pure enantiomer (see the Supplementary Materials, Table S1). Figure 3 shows the general view of compound **14**. The mean bond distances and angles in the molecules are near the expected values. In particular, the N atoms of the CON-group have a planar configuration with significant asymmetry in the N–C bonds. The N atom of the arylalkylamino group has a pyramidal configuration with a deviation of the N atom from the plane of the nearest C atoms on the 0.23 Å. The pyrrolidine moiety has, approximately, a planar configuration.

**Figure 4:**
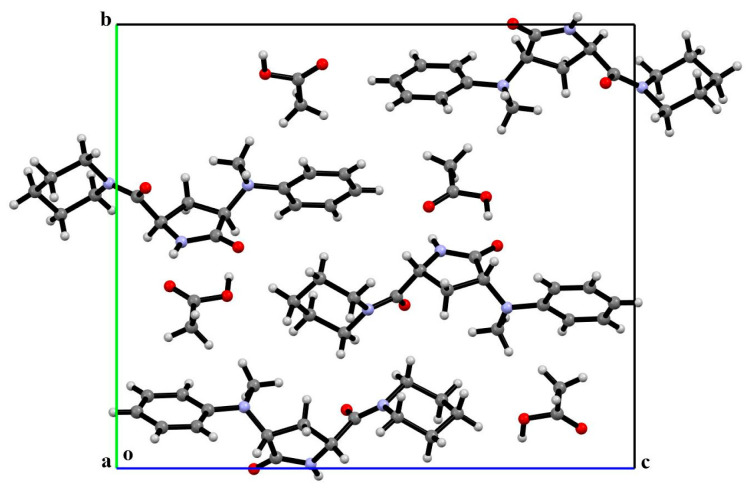
Unit cell of compound **14**; for edge dimensions a, b, and c, see the Supplementary Materials (Table S1).

It is notable that compound **12** is crystallized as solvate **14** with AcOH (1:1). The AcOH is placed near the lactam moiety C(5)O–NH and forms a dimeric intermolecular H-bond without transferring the H atoms toward the C(4)N nitrogen atom and without the formation of salt. This observation is confirmed by a strong asymmetry in the bond distances of the C–O(H) and C=O groups of the AcOH molecule. In the crystal, solvate **14** forms the layers oriented in the plane (100) (Figure 4).

The structure of compounds **2b**, **3–10**, and **12–16** was confirmed using ^1^H, ^19^F, ^13^C NMR spectroscopy and elemental analysis. For the NMR spectra of the compounds obtained, see the Supplementary Materials, Figures S1–S61.

The assignment of signals in the NMR spectra was carried out via an analogy with [15,18,19] and was confirmed using the data of 2D experiments ^1^H–^1^H HSQC, HMBC, and ^1^H–^1^H NOESY for compounds **8a** (Figures S31 and S32), **10a** (Figures S43–S45), and **14** (Figures S55–S57).

The differentiation of the C(2)H and C(4)H signals in structure **8a** is based on the analysis of the HMBC spectra (Figure S20), in which there are cross peaks between the NH-Boc protons and the C(4) carbon, on the one hand, and NH-His and C(2), on the other hand. In the absence of characteristic NH signals in the ^1^H NMR spectra, as in compounds **10a** (solution in D_2_O) and **14**, experiments based on nOe measurements were used to unambiguously assign the H-2 and H-4 signals. In the 2D NOESY spectra for compound **10a** there was a correlation between H-2 and the ortho protons of the phenyl substituent at N-1; for compound **14**, there were correlations between H-2 and the NCH_2_ of piperidine and between the H-4 and the protons of the *N*-methyl group.

The significant difference in the chemical shifts of the nonequivalent protons of the 5-oxoproline ring C(3)H^A^H^B^, which is 0.5–1.1 ppm, indicates the *cis*-configuration of the synthesized compounds **2b**, **3–10**, **12–16**, in accordance with the previously established pattern [15,18,19]. It should be noted that compounds **9a** and **9b** are poorly soluble in DMSO-*d*_6_ (0.3–0.5 mg/mL); therefore, their NMR spectra were recorded in a CF_3_CO_2_D solution. In the ^13^C NMR spectra of compounds **9a** (Figure S37) and **9b** (Figure S39), a doubling of almost all signals was observed, which was likely due to the protonation of the *N* and *O* atoms and the formation of salt forms.

It should be also noted that, in the ^1^H (or ^19^F) NMR spectra of the Boc-protected intermediates **2b**, **3a,b**, **4a,b**, **7a,b**, and **8a,b**, we observed double sets of some protons with a certain ratio of intensities. This is probably caused by the existence of conformers due to the restricted rotation around a partial double bond (HN–C(O)).

### 2.2. Antiplatelet Activity of the Studied Compounds In Vitro

The antiplatelet activity of compounds **5a,b**, **6a,b**, **9a,b**, **10a,b**, **12**, **13**, and **16** was studied on the model of adenosine-5-diphosphoric acid (ADP)-induced platelet aggregation using platelet-rich rat plasma in accordance with the method described in [20]. The tested compounds and acetylsalicylic acid (a reference drug) were dissolved in distilled water and tested at different final concentrations: 10^−5^, 10^−6^, 10^−7^, and 10^−8^ M (Table 1). After the incubation of the test compounds with the plasma, an ADP solution was added to the aggregometer cuvette, and the antiplatelet activity was evaluated. For each plasma sample, the degree of ADP-induced aggregation was first measured after their incubation with distilled water (control) and, subsequently, with the test compounds at four different dilutions in order to calculate the Δ% inhibition of platelet aggregation.

From the data presented in Table 1, it can be seen that the amides with morpholine (compounds **6b** and **13**), as well as the peptides with (*S*)-phenylalanine **9a** and **9b**, most pronouncedly inhibited platelet aggregation. The antiplatelet activity of these compounds was comparable to the activity of ASA and persisted even with further dilution to a concentration of 10^−8^ M.

### 2.3. Antithrombotic Activity of the Studied Compounds In Vivo

The antithrombotic activity of the studied compounds was assessed in vivo (Table 2), in models of arterial thrombosis and deep vein thrombosis, as well as under bleeding conditions. To evaluate the in vivo antithrombotic activity, the test compounds were administered at their most active equimolar doses (1/10 MW equivalent in mg/kg), determined at the previous stage of the study. In all three tests, the amides with morpholine **6b** and **13**, as well as the peptides with (*S*)-phenylalanine **9a** and **9b**, showed a pronounced antithrombotic activity.

All the studied compounds did not significantly affect the parameters of plasma hemostasis in the experimental animals (thrombin time (TT), prothrombin time (PT), activated partial thromboplastin time (aPTT), and fibrinogen content), which indicates the key involvement of the platelet component of hemostasis in the implementation of the antithrombotic effect of these compounds (Table 3).

### 2.4. Assessment of Acute Toxicity

Acute toxicity was determined for the compounds that showed the greatest activity: **1b**, **6a,b**, **9a,b**, **10a,b**, and **13**. Acute toxicity was expressed as a dose (LD_50_) that caused the death of 50% of the animals (white outbred male mice) within 14 days after oral administration. The LD_50_ values for compounds **9a** and **9b** were determined to be 2.5 g/kg, while for compounds **1b**, **6a,b**, **10a,b**, and **13**, the LD_50_ exceeded 3 g/kg, which indicates the low toxicity of the most active compounds.

Noteworthy is the fact that there was a slight inhibition of platelet aggregation in the in vitro experiments (Table 1), while, in the in vivo experiments, compounds **9a** and **9b** showed a pronounced antithrombotic activity (Table 2). Unfortunately, at this stage of research, i.e., the screening stage, it is impossible to accurately determine the factors that caused such a difference. These factors may include complex interactions within biological systems, potential pharmacokinetic characteristics (including the possibility for the formation of active metabolites), and other factors affecting antithrombotic activity in vivo, such as the regulation of the blood vessel tone, endothelial function, or thrombus dissolution, i.e., the aspects which are not fully registered during in vitro platelet aggregation assays. Therefore, the identified chemical structures with a noticeable activity and a low toxicity undoubtedly require further study.

## 3. Materials and Methods

### 3.1. Chemistry

#### 3.1.1. Chemistry General Section

(2*S*,4*S*)-4-Amino-5-oxo-1-phenylproline (**1a**) [18], (2*S*,4*S*)-4-amino-1-(4-fluorophenyl)-5-oxoproline (**1b**) [15], (2*S*,4*S*)-4-*tert*-butoxycarbonylamino-5-oxo-1-phenylproline (**2a**) [18], and (2*S*,4*S*)-4-[(methyl)(phenyl)amino]-5-oxoproline (**11**) [19] were obtained as previously described. The other reagents are commercially available and were purchased from Alfa Aesar (Heysham, UK). The solvents were purified according to traditional methods [21] and used freshly distilled. The melting points were obtained on a SMP3 apparatus (Barloworld Scientific, Stone, Staffordshire, UK) and are uncorrected. The optical rotations were measured on a Perkin Elmer M341 polarimeter (Perkin Elmer, Waltham, MA, USA). The reactions were monitored with thin layer chromatography (TLC) using silica gel-precoated Sorbfil plates (Imid, Krasnodar, Russia); the compounds were visualized using UV irradiation at 254 nm and iodine vapors. A flash column chromatography was performed using Silica gel 60 (230–450 mesh) (Alfa Aesar, Heysham, UK). The isolation of compounds **10a,b** was performed using ion-exchange resin Amberlite^®^ IR-120 (H^+^ form) (Acros Organics, Geel, Belgium). The ^1^H and ^19^F NMR spectra were recorded on the Bruker AVANCE 500 or Bruker DRX-400 instruments (Bruker, Karlsruhe, Germany), with operating frequencies of 500 and 476 or 400 and 376 MHz, respectively. The chemical shifts are given in ppm and are referenced to TMS (or DSS) and hexafluorobenzene as internal standards, and the multiplicities are reported as s (singlet), d (doublet), t (triplet), q (quartet), and m (multiplet). The ^13^C NMR spectra were recorded on a Bruker AVANCE 500 spectrometer (operating frequency of 126 MHz). For the NMR spectra of the compounds obtained, see the Supplementary Materials, Figures S1–S61. Elemental analyses were performed using Perkin Elmer 2400 II CHNS/O-analyzer (Perkin Elmer, Waltham, MA, USA).

#### 3.1.2. Synthesis

(2*S*,4*S*)*-*4-*tert*-Butoxycarbonylamino-1-(4-fluorophenyl)-5-oxoproline (**2b**).

A solution of Boc_2_O (2.252 g, 10.33 mmol) in *i*PrOH (27 mL) was added to a solution of compound **1b** (1.893 g, 7.95 mmol) and K_2_CO_3_ (1.206 g, 8.74 mmol) in water (66 mL) under stirring and heating at 35–40 °C. The reaction mixture was stirred at 35–40 °C for 2 h, cooled to room temperature, diluted with water (250 mL), and extracted with *n*-hexane (30 mL). The aqueous layer was acidified with citric acid hydrate to pH = 3 under stirring at room temperature. The precipitate was filtered off and washed with water (2 × 10 mL). To yield was 2.070 g (77%) of compound **2b** as colorless crystals m.p. 226–228 °C (decomp.). [α]_D_^25^ −33.8 (*c* 0.5, acetone). ^1^H NMR (500 MHz, DMSO-*d_6_*) δ (ppm) [conformers ***A*** and ***B***, 83:17]: 1.40 (s, 9H, Me-Boc), 1.91 (ddd, *J* = 11.6, 11.0, 10.2 Hz, 0.83H, H-3B, conformer ***A***), 1.96–2.06 (m, 0.17H, H-3B, conformer ***B***), 2.70 (ddd, *J* = 11.6, 8.5, 7.4 Hz, 1H, H-3A), 4.09–4.15 (m, 0.17H, H-4, conformer ***B***), 4.41 (dt, *J* = 10.9, 9.2 Hz, 0.83H, H-4, conformer ***A***), 4.82 (dd, *J* = 9.4, 7.2 Hz, 1H, H-2), 6.98 (br.d, *J* ≈ 9.0 Hz, 0.17H, NH, conformer ***B***), 7.22 (t, *J* = 8.9 Hz, 2H, H-3′,5′), 7.31 (d, *J* = 8.8 Hz, 0.83H, NH, conformer ***A***), 7.42 (dd, *J* = 9.1, 4.9 Hz, 2H, H-2′,6′), 13.07 (s, 1H, COOH). ^19^F NMR (376 MHz, DMSO-*d_6_*) δ (ppm) [conformers ***A*** and ***B***, 83:17]: 45.05–45.12 (m, 0.17F, F-4′, conformer ***B***), 45.20 (tt, *J* = 8.9, 4.5 Hz, 0.83F, F-4′ conformer ***A***). ^13^C NMR (126 MHz, DMSO-*d_6_*) δ (ppm): 28.2 (Me-Boc), 29.4 (C-3), 51.2 (C-4), 56.7 (C-2), 78.2 (C-Boc), 115.2 (d, *J* = 22.5 Hz, C-3′, C-5′), 123.4 (d, *J* = 8.4 Hz, C-2′, C-6′), 135.0 (d, *J* = 2.0 Hz, C-1′), 155.3 (NCO Boc), 159.1 (d, *J* = 241.7 Hz, C-4′), 171.7 (C-5), 172.0 (COO). Calcd (%) for C_16_H_19_FN_2_O_5_: C 56.80, H 5.66, N 8.28, F 5.62. Found (%): C 56.77, H 5.67, N 8.26, F 5.57.

General Procedure for the Synthesis of Compounds **3a,b** and **4a,b**.

HOBt hydrate (0.459 g, 3.00 mmol), piperidine (0.294 mL, 3.00 mmol) or morpholine (0.261 g, 3.00 mmol), and DIEA (1.045 mL, 6.00 mmol) were added to a suspension of compound **2a** or **2b** (3.00 mmol) in MeCN (10 mL) under stirring at room temperature. The reaction mixture was cooled to +5 °C; EDC×HCl (0.575 g, 3.00 mmol) was added. The reaction mixture was stirred for 1–3 days at room temperature, poured in water (100 mL), and extracted with CH_2_Cl_2_ (4 × 12 mL). The combined organic layers were washed successively with 5% of aqueous citric acid (3 × 5 mL), 5% of aqueous Na_2_CO_3_ (3 × 5 mL), and water (5 mL), and then evaporated to dryness under a reduced pressure.

(2*S*,4*S*)-(4-*tert*-Butoxycarbonylamino-5-oxo-1-phenylprolyl)piperidine (**3a**). 

Yield 0.912 g (78%). Colorless crystals m.p. 154–156 °C. [α]_D_^25^ −13.2 (*c* 1.0, EtOH). ^1^H NMR (500 MHz, DMSO-*d_6_*) δ (ppm) [conformers ***A*** and ***B***, 85:15]: 1.31–1.39 (m, 2H, CH_2_ piperidine), 1.40 (s, 9H, Me-Boc), 1.45–1.61 (m, 4H, 2 CH_2_ piperidine), 1.76 (q, *J* = 10.4 Hz, 0.85H, H-3B, conformer ***A***), 1.81–1.90 (m, 0.15H, H-3B, conformer ***B***), 2.66 (ddd, *J* = 11.7, 8.3, 7.1 Hz, 1H, H-3A), 3.30–3.39 (m, 2H, NCH_2_ piperidine), 3.54–3.63 (m, 2H, NCH_2_ piperidine), 4.13–4.20 (m, 0.15H, H-4, conformer ***B***), 4.42 (q, *J* = 9.4 Hz, 0.85H, H-4, conformer ***A***), 5.38 (t, *J* = 7.8 Hz, 1H, H-2), 6.86–6.90 (m, 0.15H, NH, conformer ***B***), 7.12–7.15 (m, 1H, Ph), 7.21 (d, *J* = 9.1 Hz, 085H, NH, conformer ***A***), 7.32–7.36 (m, 4H, Ph). ^13^C NMR (126 MHz, DMSO-*d_6_*) δ (ppm): 23.9, 25.3 and 26.4 (3 CH_2_ piperidine), 28.1 (Me-Boc), 29.7 (C-3), 42.6 and 45.5 (2 NCH_2_ piperidine), 51.5 (C-4), 54.3 (br. s, C-2), 78.2 (C-Boc), 121.2 (C-2′,6′), 124.6 (C-4′), 128.3 (C-3′,5′), 138.8 (C-1′), 155.3 (NCO Boc), 167.5 (C-5), 171.5 (NCO). Calcd (%) for C_21_H_29_N_3_O_4_: C 65.10, H 7.54, N 10.84. Found (%): C 65.07, H 7.48, N 10.66.

(2*S*,4*S*)-[4-*tert*-Butoxycarbonylamino-1-(4-fluorophenyl)-5-oxoprolyl]piperidine (**3b**).

Yield 0.730 g (60%). Colorless crystals m.p. 86–89 °C. [α]_D_^25^ −19.7 (*c* 1.0, EtOH). ^1^H NMR (400 MHz, DMSO-*d_6_*) δ (ppm) [conformers ***A*** and ***B***, 88:12]: 1.33–1.38 (m, 2H, CH_2_ piperidine), 1.40 (s, 9H, Me-Boc), 1.45–1.61 (m, 4H, 2 CH_2_ piperidine), 1.76 (q, *J* = 10.4 Hz, 0.88H, H-3B, conformer ***A***), 1.80–1.90 (m, 0.12H, H-3B, conformer ***B***), 2.66 (ddd, *J* = 11.7, 9.1, 7.3 Hz, 1H, H-3A), 3.28–3.39 (m, 2H, NCH_2_ piperidine, overlapped by H_2_O), 3.55 (t, *J* = 5.1 Hz, 2H, NCH_2_ piperidine), 4.10–4.20 (m, 0.12H, H-4, conformer ***B***), 4.41 (q, *J* = 9.5 Hz, 0.88H, H-4, conformer ***A***), 5.35 (dd, *J* = 8.5, 7.2 Hz, 1H, H-2), 6.85–6.91 (m, 0.12H, NH, conformer ***B***), 7.19 (t, *J* = 8.9 Hz, 2H, H-3′,5′), 7.21 (br.d, *J* = 9.7 Hz, 0.88H, NH, conformer ***A***), 7.36 (dd, *J* = 9.0, 4.9 Hz, 2H, H-′,6′). ^19^F NMR (376 MHz, DMSO-*d_6_*) δ (ppm) [conformers ***A*** and ***B***, 88:12]: 44.83–44.88 (br.m, 0.12F, F-4′, conformer ***B***), 45.02 (tt, *J* = 9.0, 4.9 Hz, 0.88F, F-4′, conformer ***A***). ^13^C NMR (126 MHz, DMSO-*d_6_*) δ (ppm): 23.9, 25.3 and 26.4 (3 CH_2_ piperidine), 28.1 (Me-Boc), 29.7 (C-3), 42.6 and 45.5 (2 NCH_2_ piperidine), 51.3 (C-4), 54.6 (br.s, C-2), 78.2 (C-Boc), 115.0 (d, *J* = 22.4 Hz, C-3′, C-5′), 123.6 (d, *J* = 8.2 Hz, C-2′, C-6′), 135.1 (d, *J* = 2.4 Hz, C-1′), 155.3 (CO-Boc), 159.0 (d, *J* = 241.7 Hz, C-4′), 167.3 (C-5), 171.6 (NCO). Calcd (%) for C_21_H_28_FN_3_O_4_: C 62.21, H 6.96, N 10.36, F 4.69. Found (%): C 61.93, H 6.89, N 10.41, F 4.61.

(2*S*,4*S*)-(4-*tert*-Butoxycarbonylamino-5-oxo-1-phenylprolyl)morpholine (**4a**).

Yield 0.806 g (69%). Colorless crystals m.p. 87–90 °C. [α]_D_^25^ −12.8 (*c* 1.0, EtOH). ^1^H NMR (500 MHz, DMSO-*d_6_*) δ (ppm) [conformers ***A*** and ***B***, 85:15]: 1.40 (s, 9H, Me-Boc), 1.79 (q, *J* = 10.3 Hz, 0.85H, H-3B, conformer ***A***), 1.83–1.93 (m, 0.15H, H-3B, conformer ***B***), 2.70 (dt, *J* = 11.7, 7.8 Hz, 1H, H-3A), 3.35–4.12 (m, 8H, 4 CH_2_ morpholine), 4.10–4.20 (br.m, 0.15H, H-4, conformer ***B***), 4.41 (q, *J* = 9.4 Hz, 0.85H, H-4, conformer ***A***), 5.35 (t, *J* = 7.6 Hz, 1H, H-2), 6.87–6.94 (br.m, 0.15H, NH, conformer ***B***), 7.13–7.16 (m, 1H, H-4′), 7.25 (d, *J* = 8.9 Hz, 0.85H, NH, conformer ***A***), 7.33–7.38 (m, 4H, Ph). ^13^C NMR (126 MHz, DMSO-*d_6_*) δ (ppm): 28.1 (Me-Boc), 29.6 (C-3), 42.1 and 45.2 (2 NCH_2_ morpholine), 51.4 (C-4), 54.2 (br.s, C-2), 66.1 and 66.2 (2 OCH_2_ morpholine), 78.2 (C-Boc), 121.3 (C-2′, C-6′), 124.7 (C-4′), 128.4 (C-3′, C-5′), 138.7 (C-1′), 155.3 (CO-Boc), 167.9 (CO-morpholine), 171.5 (C-5). Calcd (%) for C_20_H_27_N_3_O_5_: C 61.68, H 6.99, N 10.79. Found (%): C 61.49, H 6.95, N 10.54.

(2*S*,4*S*)-[4-*tert*-Butoxycarbonylamino-1-(4-fluorophenyl)-5-oxoprolyl]morpholine (**4b**). 

Yield 0.758 g (62%). Colorless crystals m.p. 102–105 °C. [α]_D_^25^ −19.1 (*c* 1.0, EtOH). ^1^H NMR (500 MHz, DMSO-*d_6_*) δ (ppm) [conformers ***A*** and ***B***, 87:13]: 1.40 (s, 9H, Me-Boc), 1.78 (q, *J* = 10.4 Hz, 0.87H, H-3B, conformer ***A***), 1.85–1.93 (m, 0.13H, H-3B, conformer ***B***), 2.70 (ddd, *J* = 11.5, 8.2, 7.5 Hz, 1H, H-3A), 3.33–3.70 (m, 8H, 4 CH_2_ morpholine), 4.11–4.17 (m, 0.13H, H-4, conformer ***B***), 4.41 (q, *J* = 9.4 Hz, 0.87H, H-4, conformer ***A***), 5.33 (dd, *J* = 8.3, 7.3 Hz, 1H, H-2), 6.90–6.94 (m, 0.13H, NH, conformer ***B***), 7.20 (t, *J* = 8.8 Hz, 2H, H-3′, H-5′), 7.26 (d, *J* = 8.9 Hz, 0.87H, NH, conformer ***A***), 7.37 (dd, *J* = 8.8, 4.9 Hz, 2H, H-2′, H-6′). ^19^F NMR (376 MHz, DMSO-*d_6_*) δ (ppm) [conformers ***A*** and ***B***, 87:13]: 44.93–45.03 (m, 0.13F, F-4′, conformer ***B***), 45.05–45.16 (m, 0.87F, F-4′, conformer ***A***). ^13^C NMR (126 MHz, DMSO-*d_6_*) δ (ppm): 28.1 (Me-Boc), 29.6 (C-3), 42.0 and 45.2 (2 NCH_2_ morpholine), 51.3 (C-4), 54.5 (br.s, C-2), 66.1 and 66.2 (2 OCH_2_ morpholine), 78.2 (C-Boc), 115.1 (d, *J* = 22.4 Hz, C-3′, C-5′), 123.6 (d, *J* = 8.2 Hz, C-2′, C-6′), 135.0 (C-1′), 155.1 (CO-Boc), 159.1 (d, *J* = 241.7 Hz, C-4′), 167.7 (CO morpholine), 171.5 (C-5). Calcd (%) for C_20_H_26_FN_3_O_5_: C 58.96, H 6.43, N 10.31, F 4.66. Found (%): C 58.70, H 6.61, N 10.35, F 4.39.

General Procedure for the Synthesis of Compounds **5a,b** and **6a,b**.

TFA (1.5 mL) was added to a solution of compounds **3a,b** and **4a,b** (2.00 mmol) in CH_2_Cl_2_ (6 mL). The reaction mixture was stirred at room temperature for 2 h, then evaporated to dryness under a reduced pressure. The residue was treated with dry diethyl ether and cooled to +5 °C. The precipitate was filtered off, dried in vacuo, and then dissolved in water (2 mL). The solution was alkalized with 1 *N* NaOH to pH = 9 and extracted with CH_2_Cl_2_ (4 × 10 mL). The combined organic layers were dried over Na_2_SO_4_ and evaporated to dryness under a reduced pressure.

(2*S*,4*S*)-(4-Amino-5-oxo-1-phenylprolyl)piperidine (**5a**).

Yield 0.471 g (81%). Light yellow hygroscopic powder m.p. 140–141 °C. [α]_D_^25^ −19.0 (*c* 1.0, H_2_O). ^1^H NMR (500 MHz, DMSO-*d_6_*) δ (ppm): 1.31–1.43 (m, 2H, CH_2_ piperidine), 1.47–1.62 (m, 5H, H-3B and 2 CH_2_ piperidine), 2.00 (br.s, 2H, NH_2_), 2.67 (dt, *J* = 12.5, 8.3 Hz, 1H, H-3A), 3.32–3.42 (m, 2H, NCH_2_ piperidine), 3.49 (dd, *J* = 8.5, 7.9 Hz, H-4), 3.54–3.63 (m, 2H, NCH_2_ piperidine), 5.35 (t, *J* = 7.2 Hz, 1H, H-2), 7.11 (t, *J* = 7.3 Hz, 1H, H-4′), 7.33 (dd, *J* = 8.4, 7.6 Hz, 2H, H-3′, H-5′), 7.41 (dd, *J* = 8.4, 1.0 Hz, 2H, H-2′, H-6′). ^13^C NMR (126 MHz, DMSO-*d_6_*) δ (ppm): 23.9, 25.3 and 26.4 (3 CH_2_ piperidine), 32.1 (C-3), 42.6 and 45.5 (2 NCH_2_ piperidine), 53.0 (C-4), 55.0 (C-2), 121.0 (C-2′, C-6′), 124.4 (C-4′), 128.4 (C-3′, C-5′), 139.0 (C-1′), 168.5 (C-5), 175.5 (NCO). Calcd (%) for C_16_H_21_N_3_O_2_ × 0.2H_2_O: C 66.05, H 7.41, N 14.44. Found (%): C 66.04, H 7.18, N 14.39.

(2*S*,4*S*)-[4-Amino-1-(4-fluorophenyl)-5-oxoprolyl] piperidine Hydrate (**5b**).

Yield 0.246 g (38%). Light yellow hygroscopic powder m.p. 140–144 °C. [α]_D_^25^ −21.0 (*c* 1.0, H_2_O). ^1^H NMR (500 MHz, DMSO-*d_6_*) δ (ppm): 1.32–1.43 (m, 2H, CH_2_ piperidine), 1.46–1.62 (m, 5H, H-3B and 2 CH_2_ piperidine), 1.96 (br.s, 2H, NH_2_), 2.68 (ddd, *J* = 12.5, 8.8, 7.8 Hz, 1H, H-3A), 3.30–3.42 (m, 2H, NCH_2_ piperidine, overlapped by H_2_O), 3.49 (t, *J* = 8.3 Hz, 1H, H-4), 3.56 (t, *J* = 5.4 Hz, 2H, NCH_2_ piperidine), 5.33 (t, *J* = 7.3 Hz, 1H, H-2), 7.19 (t, *J* = 8.9 Hz, 2H, H-3′, H-5′), 7.42 (t, *J* = 8.9 Hz, 2H, H-2′, H-6′). ^19^F NMR (470 MHz, DMSO-*d_6_*) δ (ppm): 44.74 (tt, *J* = 8.9, 4.8 Hz, F-4′). ^13^C NMR (126 MHz, DMSO-*d_6_*) δ (ppm): 23.9, 25.3 and 26.4 (3 CH_2_ piperidine), 32.2 (C-3), 42.6 and 45.5 (2 NCH_2_ piperidine), 52.8 (C-4), 55.3 (C-2), 115.1 (d, *J* = 22.3 Hz, C-3′, C-5′), 123.3 (d, *J* = 8.2 Hz, C-2′, C-6′), 135.3 (d, *J* = 2.7 Hz, C-1′), 158.9 (d, *J* = 241.4 Hz, C-4′), 168.3 (NCO), 175.5 (C-5). Calcd (%) for C_16_H_20_FN_3_O_2_ × H_2_O: C 59.43, H 6.86, N 12.99, F 5.88. Found (%): C 59.70, H 6.58, N 13.11, F 6.01.

(2*S*,4*S*)-(4-Amino-5-oxo-1-phenylprolyl)morpholine (**6a**). 

Yield 0.471 g (81%). Colorless hygroscopic powder m.p. 157–159 °C. [α]_D_^25^ −22.4 (*c* 1.0, H_2_O). ^1^H NMR (500 MHz, DMSO-*d_6_*) δ (ppm): 1.59 (ddd, *J* = 12.5, 7.5, 7.2 Hz, 1H, H-3B), 1.97 (br.s, 2H, NH_2_), 2.69 (ddd, *J* = 12.5, 8.7, 7.8 Hz, 1H, H-3A), 3.40 (t, *J* = 5.0 Hz, 2H, NCH_2_ morpholine), 3.46–3.55 (m, 3H, H-4 and NCH_2_ morpholine), 3.58–3.72 (m, 4H, 2 OCH_2_ morpholine), 5.34 (t, *J* = 7.3 Hz, 1H, H-2), 7.13 (tt, *J* = 7.3, 1.2 Hz, 1H, H-4′), 7.35 (dd, *J* = 8.6, 7.3 Hz, 2H, H-3′, H-5′), 7.42 (dd, *J* = 8.6, 1.2 Hz, 2H, H-2′, H-6′). ^13^C NMR (126 MHz, DMSO-*d_6_*) δ (ppm): 31.4 (C-3), 42.1 and 45.2 (2 NCH_2_ morpholine), 53.0 (C-4), 54.9 (br.s, C-2), 66.1 and 66.2 (2 OCH_2_ morpholine), 121.1 (C-2′, C-6′), 124.5 (C-4′), 128.4 (C-3′, C-5′), 138.9 (C-1′), 169.0 (NCO), 175.5 (C-5). Calcd (%) for C_15_H_19_N_3_O_3_ × 0.1H_2_O: C 61.88, H 6.65, N 14.43. Found (%): C 61.84, H 6.81, N 14.23.

(2*S*,4*S*)-[4-Amino-1-(4-fluorophenyl)-5-oxoprolyl]morpholine Hemihydrate (**6b**). 

Yield 0.512 g (81%). Colorless hygroscopic powder m.p. 64–66 °C. [α]_D_^25^ −27.1 (*c* 1.0, H_2_O). ^1^H NMR (500 MHz, DMSO-*d_6_*) δ (ppm): 1.58 (dt, *J* = 12.5, 7.5 Hz, 1H, H-3B), 2.01 (br.s, 2H, NH_2_), 2.70 (dt, *J* = 12.5, 8.2 Hz, 1H, H-3A), 3.40 (t, *J* = 4.9 Hz, 2H, NCH_2_ morpholine), 3.45–3.54 (m, 3H, H-4 and NCH_2_ morpholine), 3.56–3.70 (m, 4H, 2 OCH_2_ morpholine), 5.32 (t, *J* = 7.4 Hz, 1H, H-2), 7.20 (t, *J* = 8.9 Hz, 2H, H-3′, H-5′), 7.43 (dd, *J* = 9.2, 5.0 Hz, 2 H, H-2′, H-6′). ^19^F NMR (376 MHz, DMSO-*d_6_*) δ (ppm): 44.98 (tt, *J* = 8.9, 5.0 Hz, F-4′). ^13^C NMR (126 MHz, DMSO-*d_6_*) δ (ppm): 32.0 (C-3), 42.1 and 45.2 (2 NCH_2_ morpholine), 52.7 (C-4), 55.1 (C-2), 66.1 and 66.2 (2 OCH_2_ morpholine), 115.1 (d, *J* = 22.4 Hz, C-3′, C-5′), 123.4 (d, *J* = 8.2 Hz, C-2′, C-6′), 135.2 (d, *J* = 2.6 Hz, C-1′), 158.9 (d, *J* = 241.6 Hz, C-4′), 168.8 (NCO), 175.4 (C-5). Calcd (%) for C_15_H_18_FN_3_O_3_ × 0.5H_2_O: C 56.95, H 6.05, N 13.28, F 6.00. Found (%): C 57.13, H 5.97, N 13.10, F 6.12.

General Procedure for the Synthesis of Compounds **7a,b**. 

HBTU (1.138 g, 3.00 mmol) was added to a solution of compound **2a** (or **2b**) (3.00 mmol), DIEA (1.568 mL, 9.00 mmol), and methyl (*S*)-phenylalaninate hydrochloride (0.647 g, 3.00 mmol) in MeCN (10 mL) under stirring at +5 °C. The reaction mixture was stirred at room temperature for 2 days and then poured in water (100 mL). The precipitate was filtered off and washed with water (2 × 10 mL).

Methyl (2*S*,4*S*)-(4-*tert*-Butoxycarbonylamino-5-oxo-1-phenylprolyl)-(*S*)-phenylalaninate (**7a**). 

Yield 1.387 g (96%). Colorless crystals m.p. 191–193 °C. [α]_D_^25^ −20.6 (*c* 1.0, EtOH). ^1^H NMR (500 MHz, DMSO-*d_6_*) δ (ppm) [conformers ***A*** and ***B***, 88:12]: 1.41 (s, 9H, Me-Boc), 1.78 (q, *J* = 10.4 Hz, 0.88H, H-3B Pro, conformer ***A***), 1.86–1.92 (m, 0.12H, H-3B Pro, conformer ***B***), 2.57 (ddd, *J* = 12.1, 8.7, 7.6 Hz, 1H, H-3A Pro), 2.88 (dd, *J* = 13.9, 9.9 Hz, 1H, H-3B Phe), 3.04 (dd, *J* = 13.9, 5.0 Hz, 1H, H-3A Phe), 3.55 (s, 3H, OMe), 4.16–4.22 (m, 0.12H, H-4 Pro, conformer ***B***), 4.38–4.44 (m, 1.88H, 0.88H-4 Pro, conformer ***A***, and H-2 Phe), 4.72 (t, *J* = 7.8 Hz, 1H, H-2 Pro), 6.79–6.84 (m, 0.12H, NH Pro, conformer ***B***), 7.10 (t, *J* = 7.2 Hz, 1H, H-4′ Pro), 7.14 (d, *J* = 9.1 Hz, 0.88H, NH Pro, conformer ***A***), 7.16–7.31 (m, 9H, Ar), 8.89 (d, *J* = 8.0 Hz, 1H, NH Phe). ^13^C NMR (126 MHz, DMSO-*d_6_*) δ (ppm): 28.2 (Me-Boc), 29.9 (br.s, C-3 Pro), 36.2 (C-3 Phe), 51.3 (br.s, C-4 Pro), 51.9 (OMe), 53.4 (C-2 Phe), 57.4 (C-2 Pro), 78.3 (C Boc), 120.7 (C-2′, C-6′ Pro), 124.5 (br.s, C-4′ Pro), 126.6 (C-4′ Phe), 128.2 (C-3′, C-5′ Phe), 128.3 (C-3′, C-5′ Pro), 129.0 (C-2′, C-6′ Phe), 137.0 (C-1′ Phe), 138.3 (C-1′ Pro), 155.3 (CO Boc), 170.3, 171.4, and 171.9 (C-5, CO Pro, and CO Phe). Calcd (%) for C_26_H_31_N_3_O_6_: C 64.85, H 6.49, N 8.73. Found (%): C 64.65, H 6.58, N 8.93.

Methyl (2*S*,4*S*)-[4-*tert*-Butoxycarbonylamino-1-(4-fluorophenyl)-5-oxoprolyl]-(*S*)-phenylalaninate (**7b**). 

Yield 1.379 g (92%). Colorless crystals m.p. 199–200 °C. [α]_D_^25^ −26.0 (*c* 1.0, EtOH). ^1^H NMR (500 MHz, DMSO-*d_6_*) δ (ppm) [conformers ***A*** and ***B***, 88:12]: 1.41 (s, 9H, Boc), 1.78 (q, *J* = 10.4 Hz, 0.88H, H-3B Pro, conformer ***A***), 1.86–1.93 (m, 0.12H, H-3B Pro, conformer ***B***), 2.56 (dt, *J* = 11.8, 8.1 Hz, 1H, H-3A Pro), 2.86 (dd, *J* = 13.9, 9.9 Hz, 1H, H-3B Phe), 3.03 (dd, *J* = 13.9, 4.9 Hz, 1H, H-3A Phe), 3.55 (s, 3H, OMe), 4.15–4.22 (m, 0.12H, H-4 Pro, conformer ***B***), 4.38–4.43 (m, 1.88H, H-2 Phe and 0.88H-4 Pro, conformer ***A***), 4.68 (t, *J* = 7.7 Hz, 1H, H-2 Pro), 6.79–6.84 (m, 0.12H, NH Pro, conformer ***B***), 7.06 (t, *J* = 8.8 Hz, 2H, H-2′, H-6′ Pro), 7.13–7.16 (m, 3H, Ar), 7.23–7.30 (m, 5H, Ar), 8.87 (d, *J* = 8.0 Hz, 1H, NH Phe). ^19^F NMR (470 MHz, DMSO-*d_6_*) δ (ppm) [conformers ***A*** and ***B***, 88:12]: 44.84–44.92 (m, 0.12F, F-4′, conformer ***B***), 45.10 (tt, *J* = 9.0, 4.5 Hz, 0.88F, F-4′, conformer ***A***). ^13^C NMR (126 MHz, DMSO-*d_6_*) δ (ppm): 28.2 (Me-Boc), 29.9 (C-3 Pro), 36.2 (C-3 Phe), 51.2 (br.s, C-4 Pro), 52.0 (OMe), 53.3 (C-2 Phe), 57.7 (br.s, C-2 Pro), 78.3 (C Boc), 115.0 (d, *J* = 22.5 Hz, C-3′, C-5′ Pro), 123.2 (d, *J* = 8.0 Hz, C-2′, C-6′ Pro), 126.5 (C-4′ Phe), 128.2 (C-3′, C-5′ Phe), 129.0 (C-2′, C-6′ Phe), 134.6 (d, *J* = 1.8 Hz, C-1′ Pro), 137.0 (C-1′ Phe), 155.3 (CO Boc), 159.0 (d, *J* = 242.1 Hz, C-4′ Pro), 170.0, 171.4, and 171.9 (CO Glp, C-5 Glp, CO Phe). Calcd (%) for C_26_H_30_FN_3_O_6_: C 62.52, H 6.05, N 8.41, F 3.80. Found (%): C 62.56, H 6.09, N 8.61, F 3.82.

General Procedure for the Synthesis of Compounds **8a,b**. TBTU (0.960 g, 3.00 mmol) was added to a solution of compound **2a** (or **2b**) (3.00 mmol), DIEA (2.090 mL, 12.0 mmol), and methyl (*S*)-histidinate dihydrochloride (0.726 g, 3.00 mmol) in MeCN (10 mL) under stirring at +5 °C. The reaction mixture was stirred at room temperature for 3 days, poured in water (100 mL), and extracted with CH_2_Cl_2_ (4 × 12 mL). The combined organic layers were washed with 5% aqueous Na_2_CO_3_ (3 × 5 mL) and water (5 mL), dried over Na_2_SO_4_, and evaporated to dryness under a reduced pressure. The residue was dried in vacuo.

Methyl (2*S*,4*S*)-(4-*tert*-Butoxycarbonylamino-5-oxo-1-phenylprolyl)-(*S*)-histidinate (**8a**). 

Yield 0.891 g (63%). Colorless crystals m.p. 226–228 °C (decomp.). [α]_D_^25^ −38.5 (*c* 1.0, EtOH). ^1^H NMR (400 MHz, DMSO-*d_6_*) δ (ppm) [conformers ***A*** and ***B***, 84:16]: 1.41 (s, 9H, Me-Boc), 1.81 (br.q, *J* = 10.2 Hz, 0.84H, H-3B Pro, conformer ***A***), 1.88–1.98 (m, 0.16H, H-3B Pro, conformer ***B***), 2.58 (dt, *J* = 11.7, 8.0 Hz, 1H, H-3A Pro), 2.83 (dd, *J* = 14.6, 8.9 Hz, 1H, H-3B His), 2.92 (dd, *J* = 14.6, 5.3 Hz, 1H, H-3A His), 3.55 (s, 3H, OCH_3_), 4.14–4.26 (m, 0.16H, H-4 Pro, conformer ***B***), 4.38–4.46 (m, 1.84H, H-2 His and H-4 Pro, conformer ***A***), 4.75 (t, *J* = 7.7 Hz, 1H, H-2 Pro), 6.77 (s, 1H, H-5′, His), 6.80–6.86 (m, 0.16H, NH Pro, conformer ***B***), 7.11 (t, *J* = 7.1 Hz, 1H, H-4′ Pro), 7.16 (d, *J* = 9.0 Hz, 0.84H, NH Pro, conformer ***A***), 7.26–7.35 (m, 4H, Ar), 7.57 (d, *J* = 1.0 Hz, 1H, H-2′ His), 8.83 (d, *J* = 7.7 Hz, 1H, CONH His), 11.5–12.5 (br.s, 1H, NH imidazole). ^13^C NMR (126 MHz, DMSO-*d_6_*) δ (ppm): 28.1 (Me-Boc), 28.7 (C-3 His), 29.9 (C-3 Pro), 51.3 (C-4 Pro), 51.8 (OMe), 52.2 (C-2 His), 57.4 (C-2 Pro), 78.2 (C-Boc), 116.3 (C-5′ His), 120.8 (C-2′, C-6′ Pro), 124.5 (C-4′ Pro), 128.3 (C-3′, C-5′ Pro), 133.0 (C-4′ His), 134.9 (C-2′ His), 138.3 (C-1′ Pro), 155.2 (CO Boc), 170.1 (CO Pro), 171.4 (CO His), 171.8 (C-5 Pro). Calcd (%) for C_23_H_29_N_5_O_6_: C 58.59, H 6.20, N 14.85. Found (%): C 58.58, H 6.10, N 14.88.

Methyl (2*S*,4*S*)-[4-*tert*-Butoxycarbonylamino-1-(4-fluorophenyl)-5-oxoprolyl]-(*S*)-histidinate Hemihydrate (**8b**). 

Yield 0.793 g (53%). Colorless crystals m.p. 205–207 °C (water) (decomp.). [α]_D_^25^ −39.4 (*c* 1.0, EtOH). ^1^H NMR (500 MHz, DMSO-*d_6_*) δ (ppm) [conformers ***A*** and ***B***, 87:13]: 1.40 (s, 9 H, Me-Boc), 1.81 (br.q, *J* = 10.2 Hz, 0.87H, H-3B Pro, conformer ***A***), 1.88–1.98 (br.m, 0.13H, H-3B Pro, conformer ***B***), 2.58 (ddd, *J* = 12.0, 8.7, 7.6 Hz, 1H, H-3A Pro), 2.82 (dd, *J* = 14.6, 8.8 Hz, 1H, H-3B His), 2.91 (dd, *J* = 14.6, 5.1 Hz, 1H, H-3A His), 3.55 (s, 3H, OMe), 4.13–4.23 (br.m, 0.13H, H-4 Pro, conformer ***B***), 4.39–4.45 (m, 1.87H, H-2 His and H-4 Pro, conformer ***A***), 4.72 (t, *J* = 7.7 Hz, 1H, H-2 Pro), 6.76 (s, 1H, H-5′ His), 6.81–6.86 (br.s, 0.13H, NH Boc, conformer ***B***), 7.13 (t, *J* = 8.8 Hz, 2H, H-3′, H-5′ Pro), 7.17 (d, *J* = 8.9 Hz, 0.87H, NH Boc, conformer ***A***), 7.33 (dd, *J* = 9.0, 4.9 Hz, 2H, H-2′, H-6′ Pro), 7.55 (d, *J* = 1.0 Hz, 1H, H-2′ His), 8.81 (d, *J* = 7.7 Hz, 1H, CONH His), 11.86 (br.s, 1H, NH imidazole). ^19^F NMR (376 MHz, DMSO-*d_6_*) δ (ppm) [conformers ***A*** and ***B***, 87:13]: 44.82–44.93 (m, 0.13F, F-4′, conformer ***B***), 45.06 (tt, *J* = 8.8, 4.9 Hz, 0.87F, F-4′, conformer ***A***). ^13^C NMR (126 MHz, DMSO-*d_6_*) δ (ppm): 28.1 (Me Boc), 28.7 (C-3 His), 29.9 (C-3 Pro), 51.2 (C-4 Pro), 51.8 (OMe), 52.2 (C-2 His), 57.7 (C-2 Pro), 78.3 (C Boc), 115.0 (d, *J* = 22.4 Hz, C-3′, C-5′ Pro), 116.4 (C-5′ His), 123.0 (d, *J* = 7.8 Hz, C-2′, C-6′ Pro), 133.0 (C-4′ His), 134.6 (C-1′ Pro), 134.9 (C-2′ His), 155.3 (CO Boc), 158.9 (d, *J* = 241.8 Hz, C-4′ Pro), 169.9 (CO Pro), 171.4 (CO His), 171.9 (C-5 Pro). Calcd (%) for C_23_H_28_FN_5_O_6_ × 0.5H_2_O: C 55.42, H 5.86, N 14.05, F 3.81. Found (%): C 55.15, H 5.96, N 13.95, F 4.05.

General Procedure for the Synthesis of Compounds **9a,b** and **10a,b**. 

TFA (2 mL) was added to a solution of an appropriate protected dipeptide **7a,b** (or **8a,b**) (2.00 mmol) in CH_2_Cl_2_ (6 mL) under stirring at room temperature. The reaction mixture was stirred at room temperature for 2 h and evaporated to dryness under a reduced pressure. The residue was treated with dry diethyl ether; the precipitate was filtered off and dried in vacuo, then dissolved in 1 N NaOH (6.0 mL, 6.0 mmol). The reaction mixture was kept at room temperature for 4 h. In the case of compounds **9a,b**, the reaction mixture was acidified with 1 M HCl to a pH 3–4 and kept overnight at +5 … +10 °C; the precipitate was filtered off, washed with water (2 × 2 mL), and dried in vacuo. In the case of compounds **10a,b**, the reaction mixture was acidified with TFA to a pH 4. The resulting solution was treated with ion-exchange resin Amberlite^®^ IR-120(H); the resin was washed with water; then, the target compound was eluted with a water–pyridine 9:1 mixture. The eluate was evaporated to dryness under a reduced pressure.

(2*S*,4*S*)-(4-Amino-5-oxo-1-phenylprolyl)-(*S*)-phenylalanine Hemihydrate (**9a**).

Yield 0.587 g (78%). Colorless crystals m.p. 279–281 °C (decomp.). [α]_D_^25^ +97.6 (*c* 1.0, TFA). ^1^H NMR (500 MHz, CF_3_CO_2_D) δ (ppm): 2.70 (dt, *J* = 15.1, 3.5 Hz,1H, H-3B Pro), 3.09 (dd, *J* = 14.4, 4.7 Hz, 1H, H-3B Phe), 3.17 (dd, *J* = 14.4, 6.4 Hz, 1H, H-3A Phe), 3.23 (dt, *J* = 15.1, 8.0 Hz, 1H, H-3A Pro), 4.71 (dd, *J* = 7.8, 3.4 Hz, 1H, H-2 Pro), 5.04 (t, *J* = 5.5 Hz, 1H, H-2 Phe), 5.18 (dd, *J* = 8.0, 3.2 Hz, 1H, H-4 Pro), 6.71 (d, *J* = 7.5 Hz, 2H, H-2′, H-6′ Pro), 7.18 (t, *J* = 7.5 Hz, 2H, H-3′, H-5′ Pro), 7.26 (t, *J* = 7.4 Hz, 1H, H-4′ Pro), 7.31–7.36 (m, 2H, Ar), 7.55–7.59 (m, 3H Ar). ^13^C NMR (126 MHz, CF_3_CO_2_D) δ (ppm): 29.1 (C-3 Pro), 38.5 (C-3 Phe), 53.7 (br.s, C-4 Pro), 56.0 and 56.1 (C-2 Phe), 64.9 and 65.0 (C-2 Pro), 126.7 (C-2′, C-6′ Pro), 129.2 (C-4′ Pro), 130.50 and 130.54 (C-3′, C-5′ and C-2′, C6′ Phe), 131.6 (C-4′ Phe), 131.9 (C-3′, C-5′ Pro), 136.1 and 136.2 (C-1′ Pro), 172.1 (br.s, CO Pro), 173.36 and 173.44 (C-5, Pro), 175.97 and 175.99 (COOH Phe). Calcd (%) for C_20_H_21_N_3_O_4_×0.5H_2_O: C 63.82, H 5.89, N 11.16. Found (%): C 64.08, H 5.59, N 11.15.

(2*S*,4*S*)-[4-Amino-1-(4-fluorophenyl)-5-oxoprolyl]-(*S*)-phenylalanine (**9b**).

Yield 0.570 g (74%). Colorless crystals m.p. 298–301 °C (decomp.). [α]_D_^25^ +86.9 (*c* 1.0, TFA). ^1^H NMR (500 MHz, CF_3_CO_2_D) δ (ppm): 2.68 (dt, *J* = 15.2, 3.4 Hz, 1H, H-3B Pro), 3.12 (dd, *J* = 14.5, 7.5 Hz, 1H, H-3B Phe), 3.18–3.24 (m, 2H, H-3A Phe, H-3A Pro), 4.70 (dd, *J* = 8.8, 4.4 Hz, 1H, H-2 Pro), 5.02 (dd, *J* = 7.3, 4.7 Hz, 1H, H-2 Phe), 5.12 (dd, *J* = 7.8, 3.5 Hz, 1H, H-4 Pro), 6.82 (d, *J* = 7.7 Hz, 2H Ar), 7.17–7.23 (m, 4H Ar), 7.27–7.32 (m, 3H, Ar). ^13^C NMR (126 MHz, CF_3_CO_2_D) δ (ppm): 29.31 and 29.35 (C-3 Pro), 38.8 (C-3 Phe), 54.3 and 54.4 (C-4 Pro), 56.6 and 56.7 (C-2 Phe), 65.4 and 66.2 (C-2 Pro), 119.2 (d, *J* = 23.6 Hz, C-3′, C5′ Pro), 129.0 (d, *J* = 9.1 Hz, C-2′, C-6′ Pro), 129.9 (C-4′ Phe), 130.6 (C-2′, C-6′ Phe), 131.0 (C-3′, C-5′ Phe), 132.0 (d, *J* = 2.9 Hz, C-1′ Pro), 135.6 and 135.8 (C-1′ Phe), 165.3 (d, *J* = 246.7 Hz, C-4′ Pro), 172.6 and 172.7 (CO Pro), 173.9 and 174.0 (C-5 Pro), 177.48 and 177.49 (COOH Phe). ^19^F NMR (376 MHz, DMSO-*d_6_*) δ (ppm): 44.92–45.07 (m, F-4′). Calcd (%) for C_20_H_20_FN_3_O_4_×0.1H_2_O: C 62.04, H 5.26, N 10.85, F 4.91. Found (%): C 61.78, H 5.21, N 10.76, F 4.87.

(2*S*,4*S*)-(4-Amino-5-oxo-1-phenylprolyl)-(*S*)-histidine Hemihydrate (**10a**). 

Yield 0.462 g (63%). Slightly colored crystals m.p. 255–257 °C (decomp.). [α]_D_^25^ −30.3 (*c* 1.0, H_2_O). ^1^H NMR (500 MHz, D_2_O) δ (ppm): 2.04 (ddd, *J =* 13.3, 8.9, 7.6 Hz, 1H, H-3B Pro), 2.85 (dd, *J =* 15.6, 10.0 Hz, 1H, H-3B His), 2.98 (ddd, *J =* 13.3, 9.0, 7.4 Hz, 1H, H-3A Pro), 3.08 (dd, *J =* 15.6, 4.0 Hz, 1H, H-3A His), 4.05 (t, *J =* 9.0 Hz, 1H, H-4 Pro), 4.38 (dd, *J =* 10.0, 4.0 Hz, 1H, H-2 His), 4.89 (t, *J =* 7.5 Hz, 1H, H-2 Pro), 6.65 (s, 1H, H-5′ His), 7.20–7.22 (m, 2H, H-2′, H-6′ Pro), 7.37–7.43 (m, 3H, Ar), 8.06 (s, 1H, H-2′ His). ^13^C NMR (126 MHz, D_2_O) δ (ppm): 30.6 (C-3 His), 32.2 (C-3 Pro), 53.9 (C-4 Pro), 57.1 (C-2 His), 63.2 (C-2 Pro), 119.1 (C-5′ His), 127.0 (C-2′, C-6′ Pro), 130.7 (C-4′ Pro), 132.1 (C-3′, C-5′ Pro), 133.4 (C-4′ His), 136.7 (C-2′ His), 138.3 (C-1′ Pro), 173.7 (CO Pro), 177.1 (C-5 Pro), 179.1 (CO His). Calcd (%) for C_17_H_19_N_5_O_4_×0.5H_2_O: C 55.73, H 5.50, N 19.12. Found (%): C 55.86, H 5.43, N 18.78.

(2*S*,4*S*)-[4-Amino-1-(4-fluorophenyl)-5-oxoprolyl]-(*S*)-histidine Hydrate (**10b**). 

Yield 0.529 g (68%). Slightly colored crystals m.p. 235–236 °C (decomp.). [α]_D_^25^ − 32.5 (*c* 1.0, H_2_O). ^1^H NMR (500 MHz, D_2_O) δ (ppm): 2.05 (ddd, *J =* 13.3, 8.9, 7.6 Hz, 1H, H-3B Pro), 2.85 (dd, *J =* 15.7, 10.1 Hz, 1H, H-3B His), 2.96 (ddd, *J =* 13.3, 9.1, 7.3 Hz, 1H, H-3A Pro), 3.08 (dd, *J =* 15.7, 4.0 Hz, 1H, H-3A His), 4.04 (t, *J =* 9.0 Hz, 1H, H-4 Pro), 4.38 (dd, *J =* 10.1, 4.0 Hz, 1H, H-2 His), 4.82 (t, *J =* 7.4 Hz, 1H, H-2 Pro), 6.73 (s, 1H, H-5′ His), 7.11 (t, *J* = 8.8 Hz, 2H, H-3′, H-5′ Pro), 7.20 (dd, *J* = 9.0, 4.9 Hz, 2H, H-2′, H-6′ Pro), 8.06 (s, 1H, H-2′ His). ^13^C NMR (126 MHz, D_2_O) δ (ppm): 30.7 (C-3 His), 32.2 (C-3 Pro), 53.9 (C-4 Pro), 57.1 (C-2 His), 63.5 (C-2 Pro), 118.9 (d, *J* = 23.0 Hz, C-3′, C-5′ Pro), 119.0 (C-5′ His), 129.5 (d, *J* = 8.9 Hz, C-2′, C-6′ Pro), 133.9 (C-4′ His), 134.3 (d, *J* = 2.8 Hz, C-1′ Pro), 136.9 (C-2′ His), 164.0 (d, *J* = 245.4 Hz, C-4′ Pro), 173.5 (CO Pro), 177.5 (C-5 Pro), 179.20 (CO His). ^19^F NMR (376 MHz, D_2_O) δ (ppm): 48.82 (tt, *J* = 8.5, 4.9 Hz, F-4′ Pro). Calcd (%) for C_17_H_18_N_5_O_4_F×0.75H_2_O: C 52.51, H 5.05, N 18.01, F 4.88. Found (%): C 52.73, H 4.81, N 17.88, F 5.01.

(2*S*,4*S*)-4-[(Methyl)(phenyl)amino]-5-oxoprolylpiperidine (**12**).

Piperidine (1.395 mL, 14.12 mmol) and HBTU (1.953 g, 5.15 mmol) were added to a solution of compound **11** (1.00 g, 4.27 mmol) in DMF (12 mL) under stirring at 0 °C. The reaction mixture was stirred at room temperature for 1 day; then, ethyl acetate (130 mL) was added, and the reaction mixture was washed with 5% aqueous citric acid (6 × 25 mL), 5% aqueous Na_2_CO_3_ (6 × 25 mL), and saturated NaCl solution (3 × 25 mL), dried over Na_2_SO_4_, and evaporated to dryness under a reduced pressure. The residue was purified with flash column chromatography on silica gel (benzene–EtOAc 10:1 to EtOAc as an eluent). The slow-eluting fractions were combined, evaporated to dryness under a reduced pressure, and treated with *n*-hexane to afford 0.772 g (60%) of compound **12** as colorless crystals m.p. 147–148 °C. [α]_D_^25^ +135.0 (*c* 1.0, CHCl_3_). ^1^H NMR (500 MHz, CDCl_3_) δ (ppm): 1.46–1.68 (m, 6H, piperidine), 2.00 (ddd, *J* = 12.8, 9.9, 9.2 Hz, 1H, H-3B), 2.74 (ddd, *J* = 12.8, 9.8, 9.0 Hz, 1H, H-3A), 2.85 (s, 3H, NMe), 3.31–3.41 (m, 2H, NCH_2_, piperidine), 3.47 (ddd, *J* = 12.9, 7.9, 3.8 Hz, 1H, NCH piperidine), 3.64 (ddd, *J* = 12.9, 6.7, 4.3 Hz, 1H, NCH piperidine), 4.40 (dd, *J* = 9.0, 7.5 Hz, 1H, H-2), 4.70 (t, *J* = 9.2 Hz, 1H, H-4), 6.74–6.78 (m, 2H, NH, H-4′), 6.81 (d, *J* = 8.8 Hz, 2H, H-2′, H-6′), 7.23 (dd, *J* = 8.8, 7.3 Hz, 2H, H-3′, H-5′). ^13^C NMR (126 MHz, CDCl_3_) δ (ppm): 24.3, 25.4 and 26.4 (3 CH_2_ piperidine), 28.7 (C-3), 33.8 (NMe), 43.4 and 45.8 (2 NCH_2_ piperidine), 50.7 (C-2), 60.3 (C-4), 113.5 (C-2′,6′), 117.8 (C-4′), 129.1 (C-3′, C-5′), 149.5 (C-1′), 168.5 (NCO), 173.8 (C-5). Calcd (%) for C_17_H_23_N_3_O_2_: C 67.75, H 7.69, N 13.94. Found (%): C 67.89, H 7.62, N 13.88.

(2*S*,4*S*)-4-[(Methyl)(phenyl)amino]-5-oxoprolylmorpholine (**13**).

Morpholine (1.33 mL, 15.37 mmol) and HBTU (1.943 g, 5.12 mmol) were added to a solution of compound **11** (1.00 g, 4.27 mmol) in CH_2_Cl_2_ (20 mL) under stirring at 0 °C. The reaction mixture was stirred at room temperature for 1 day and evaporated to dryness under a reduced pressure. At first, the residue was purified with flash column chromatography on silica gel (CHCl_3_–MeOH–TEA from 10:0.1:0.2 to 10:0.3:0.2 as eluent). The slow-eluting fractions were combined and evaporated to dryness under a reduced pressure. The residue was once more subjected to flash column chromatography on silica gel (benzene–EtOAc 85:15 → EtOAc → EtOAc–MeOH 100:1). The slow-eluting fractions were combined and evaporated to dryness to afford 1.049 g (81%) of compound **13** as a semisolid. [α]_D_^25^ +114.9 (*c* 1.0, CHCl_3_). ^1^H NMR (500 MHz, CDCl_3_) δ (ppm): 1.99 (ddd, *J* = 12.8, 9.8, 9.0 Hz, 1H, H-3B), 2.71 (ddd, *J* = 12.8, 8.7, 7.5 Hz, 1H, H-3A), 2.84 (s, 3H, NMe), 3.38–3.49 (m, 2H, CH_2_ morpholine), 3.58–3.73 (m, 6H, 3 CH_2_ morpholine), 4.45 (dd, *J* = 9.0, 7.5 Hz, 1H, H-2), 4.74 (t, *J* = 9.2 Hz, 1H, H-4), 6.78 (t, *J* = 7.3 Hz, 1H, H-4′), 6.82 (d, *J* = 8.8 Hz, 2H, H-2′, H-6′), 6.98 (br.s, 1H, NH), 7.24 (dd, *J* = 8.8, 7.3 Hz, 2H, H-3′, H-5′). ^13^C NMR (126 MHz, CDCl_3_) δ (ppm): 28.0 (C-3), 33.9 (NMe), 42.5 and 45.3 (2 NCH_2_ morpholine), 50.7 (C-2), 60.3 (C-4), 66.4 and 66.6 (2 OCH_2_ morpholine), 113.5 (C-2′, C-6′), 118.0 (C-4′), 129.3 (C-3′, C-5′), 149.2 (C-1′), 169.3 (NCO), 174.8 (C-5). Calcd (%) for C_16_H_21_N_3_O_3_: C 63.35, H 6.98, N 13.85. Found (%): C 63.16, H 7.05, N 13.74.

(2*S*,4*S*)-4-[(Methyl)(phenyl)amino]-5-oxoprolylpiperidine, Solvate with AcOH (**14**).

AcOH (57 μL, 1.0 mmol) and hot *n*-hexane (30 mL) were added to a solution of compound **12** (0.301 g, 1.0 mmol) in ethyl acetate (3 mL). The reaction mixture was kept at −10 °C for 3 days. The precipitate was filtered off and washed with *n*-hexane–EtOAc 10:1 (5 mL) to afford 0.253 g (70%) of solvate **14** as large, colorless, transparent blocks with m.p. 87–89 °C. [α]_D_^25^ +106.0 (*c* 1.0, CHCl_3_). ^1^H NMR (500 MHz, CDCl_3_) δ (ppm): 1.49–1.70 (m, 6H, 3 CH_2_ piperidine), 2.04 (ddd, *J* = 12.9, 9.7, 9.2 Hz, 1H, H-3B), 2.07 (s, 3H, CH_3_), 2.74 (ddd, *J* = 12.9, 8.3, 7.7 Hz, 1H, H-3A), 2.86 (s, 3H, NMe), 3.35–3.43 (m, 2H, NCH_2_ piperidine), 3.53 (ddd, *J* = 13.0, 7.6, 4.2 Hz, 1H, NCH^B^ piperidine), 3.63 (ddd, *J* = 13.0, 6.9, 4.2 Hz, 1H, NCH^A^ piperidine), 4.43 (dd, *J* = 8.8, 7.6 Hz, 1H, H-2), 4.73 (t, *J* = 9.2 Hz, 1H, H-4), 6.77 (t, *J* = 7.3 Hz, 1H, H-4′), 6.82 (d, *J* = 8.2 Hz, 2H, H-2′, H-6′), 6.98 (br.s, 1H, NH), 7.24 (dd, *J* = 8.8, 7.3 Hz, 2H, H-3′, H-5′), 11.12 (br.s, 1H, CO_2_H). ^13^C NMR (126 MHz, CDCl_3_) δ (ppm): 20.8 (CH_3_), 24.0 (C-4′′), 25.2 and 26.4 (C-3′′, C-5′′), 58.5 (C-3), 33.7 (NMe), 43.4 and 45.8 (C-2″, C-6″), 50.7 (C-2), 60.2 (C-4), 113.4 (C-2′, C-6′), 117.6 (C-4′), 129.1 (C-3′, C-5′), 149.4 (C-1′), 168.5 (NCO), 174.2 (C-5), 174.6 (COO). Calcd (%) for C_19_H_27_N_3_O_4_: C 63.14, H 7.53, N 11.62. Found (%): C 63.14, H 7.72, N 11.63.

*tert*-Butyl (*2S*,4*S*)-4-[(Methyl)(phenyl)amino]-5-oxoprolyl-(*S*)-phenylalaninate (**15**).

*tert-*Butyl (*S*)-phenylalaninate hydrochloride (1.592 g, 6.18 mmol), HBTU (2.342 g, 6.18 mmol), and DIEA (3.05 mL, 17.51 mmol) were added to a solution of compound **11** (1.205 g, 5.14 mmol) in DMF (10 mL) under stirring at 0 °C. The reaction mixture was stirred at room temperature for 1 day; then, ethyl acetate (150 mL) was added, and the reaction mixture was washed with 5% aqueous citric acid (6 × 25 mL), 5% aqueous Na_2_CO_3_ (6 × 25 mL), and saturated NaCl solution (3 × 25 mL), dried over Na_2_SO_4_, and evaporated to a volume of 20 mL under a reduced pressure. Hot *n*-hexane (50 mL) was added, and the reaction mixture was kept at +10 °C for 3 days. The precipitate was filtered off and washed with *n*-hexane–EtOAc 2:5 (10 mL) to afford 1.350 g (60%) of compound **15**. An additional amount (0.670 g, 30%) was isolated from the filtrate after flash column chromatography on silica gel (benzene–EtOAc 6:4 as an eluent). Total yield 2.020 g (90%). Colorless crystals m.p. 130–133 °C. [α]_D_^25^ +71.7 (*c* 1.0, CHCl_3_). ^1^H NMR (500 MHz, DMSO-*d_6_*) δ (ppm): 1.31 (s, 9H, *t*Bu), 1.64 (ddd, *J* = 12.8, 9.2, 8.1 Hz, 1H, H-3B Pro), 2.60 (ddd, *J* = 12.8, 9.1, 8.0 Hz, 1H, H-3A Pro), 2.68 (s, 3H, NMe), 2.94 (dd, *J* = 13.8, 8.9 Hz, 1H, H-3B Phe), 3.01 (dd, *J* = 13.8, 6.0 Hz, 1H, H-3A Phe), 4.02 (t, *J* = 8.0 Hz, 1H, H-2 Pro), 4.40 (ddd, *J* = 8.9, 7.8, 6.0 Hz, 1H, H-2 Phe), 4.65 (t, *J* = 9.2 Hz, 1H, H-4 Pro), 6.65 (t, *J* = 7.2 Hz, 1H, H-4′ Pro), 6.78 (d, *J* = 8.8 Hz, 2H, H-2′, H-6′ Pro), 7.16 (dd, *J* = 8.8, 7.2 Hz, 2H, H-3′, H-5′ Pro), 7.18–7.29 (m, 5H, Ph), 8.13 (s, 1H, NH Pro), 8.40 (d, *J* = 7.8 Hz, 1H, NH Phe). ^13^C NMR (126 MHz, DMSO-*d_6_*) δ (ppm): 27.4 (CH_3_ *t*Bu), 27.9 (C-3 Pro), 33.2 (NMe), 36.6 (C-3 Phe), 51.9 (C-2 Pro), 54.1 (C-2 Phe), 59.0 (C-4 Pro), 80.7 (C *t*Bu), 112.6 (C-2′, C-6′ Pro), 116.5 (C-4′ Pro), 126.4 (C-4′ Phe), 128.1 (C-2′, C-6′ Phe), 128.8 (C-3′, C-5′ Pro), 129.1 (C-3′, C-5′ Phe), 137.2 (C-1′ Phe), 149.5 (C-1′ Pro), 170.3 (CO Phe), 171.6 (NCO Pro), 173.8 (C-5). Calcd (%) for C_25_H_31_N_3_O_4_: C 68.63, H 7.14, N 9.60. Found (%): C 68.77, H 7.19, N 9.32.

(2*S*,4*S*)-4-[(Methyl)(phenyl)amino]-5-oxoprolyl-(*S*)-phenylalanine (**16**).

A solution of compound **15** (1.080 g, 2.47 mmol) in CF_3_CO_2_H (15 mL) was stirred at room temperature for 2 h and evaporated to dryness under a reduced pressure. The residue was stirred with water (15 mL) for 30 min, evaporated again, dried in vacuo, then crystallized from ethyl acetate (30 mL). The resulting precipitate of the product contained ~30 mol.% of EtOAc (according to ^1^H NMR); so, it was treated with water (40 mL) for 30 min in an ultrasonic bath (100 W), filtered off, washed with water (5 mL), and dried in vacuo to afford 0.725 g (76%) of compound **16** as a light gray powder with m.p. 121–123 °C. [α]_D_^25^ +51.7 (*c* 1.0, DMSO). ^1^H NMR (500 MHz, DMSO-*d_6_*) δ (ppm): 1.59 (ddd, *J* = 12.7, 9.4, 8.2 Hz, 1H, H-3B Pro), 2.57 (ddd, *J* = 12.7, 9.0, 8.1 Hz, 1H, H-3A Pro), 2.66 (s, 3H, NMe), 2.93 (dd, *J* = 13.8, 9.7 Hz, 1H, H-3B Phe), 3.09 (dd, *J* = 13.8, 4.8 Hz, 1H, H-3A Phe), 3.99 (t, *J* = 8.1 Hz, 1H, H-2 Pro), 4.47 (ddd, *J* = 9.7, 8.1, 4.8 Hz, 1H, H-2 Phe), 4.64 (t, *J* = 9.2 Hz, 1H, H-4 Pro), 6.65 (t, *J* = 7.2 Hz, 1H, H-4′ Pro), 6.78 (d, *J* = 8.7 Hz, 2H, H-2′, H-6′ Pro), 7.16 (dd, *J* = 8.7, 7.2 Hz, 2H, H-3′, H-5′ Pro), 7.18–7.21 (m, 1H, Ph), 7.23–7.28 (m, 4H, Ph), 8.11 (s, 1H, NH Pro), 8.33 (d, *J* = 8.1 Hz, 1H, NH Phe), 12.78 (br.s, 1H, CO_2_H). ^13^C NMR (126 MHz, DMSO-*d_6_*) δ (ppm): 28.0 (C-3 Pro), 33.2 (NMe), 36.4 (C-3 Phe), 52.1 (C-2 Pro), 53.4 (C-2 Phe), 59.0 (C-4 Pro), 112.6 (C-2′, C-6′ Pro), 116.5 (C-4′ Pro), 126.4 (C-4′ Phe), 128.2 (C-2′, C-6′ Phe), 128.9 (C-3′, C-5′ Pro), 129.1 (C-3′, C-5′ Phe), 137.6 (C-1′ Phe), 149.6 (C-1′ Pro), 171.7 (NCO Pro), 172.7 (COOH Phe), 174.0 (C-5). Calcd (%) for C_21_H_23_N_3_O_4_×0.3H_2_O: C 65.20, H 6.15, N 10.86. Found (%): C 65.22, H 6.10, N 10.91.

#### 3.1.3. X-ray Diffraction Analysis

The X-ray diffraction study of solvate **14** was carried out according to a standard procedure using an Xcalibur 3 diffractometer (Oxford Diffraction, London, UK) equipped with a CCD detector (λ(Mo-*K*α) = 0.71073 Å, graphite monochromator, ω/2θ scan mode, scanning increment 1°, *T* = 295(2) K). The empirical absorption correction was applied. The structures were solved with direct methods using the OLEX2 software package [22]. The structures were solved using the method of the intrinsic phases in the ShelXT program and refined with ShelXL using the full-matrix least-squared method for non-hydrogen atoms [23]. The hydrogen atoms of the C–H bonds were placed in the calculated positions and were refined in an isotropic approximation. The selected X-ray single-crystal data and structure refinement details are summarized in Table S1. The crystallographic data for solvate **14** have been deposited in the Cambridge Crystallographic Data Centre (CCDC No. 2290217). Copies of the data can be obtained, free of charge, by applying to the CCDC, 12 Union Road, Cambridge CB2 1EZ, UK (https://www.ccdc.cam.ac.uk/structures/; accessed on 1 October 2023).

### 3.2. Study of Biological Activity

#### 3.2.1. General Section

The study of the biological activity of the compounds was carried out in 239 outbred male rats weighing 250–270 g and on 127 outbred male mice (Stolbovaya Branch of the Scientific Center for Biomedical Technologies of the Federal Medical and Biological Agency, Moscow Region, Russia). The age of the animals from which blood was taken for the in vitro studies was 18–24 months. The male rats aged 6–8 months were used for the studies of antiplatelet and antithrombotic activity in vivo. The animals were kept under standard vivarium conditions (22–24 °C, 40–50% humidity, ambient light) during the study. All painful manipulations were performed under general anesthesia with a single intraperitoneal injection of zolazepam 20 mg/kg (Zoletil^®^100; Valdepharm, Val-de-Reuil, France) + xylazine 8 mg/kg (Xyla; Interchemie, Venray, The Netherlands). At the end of the experiment, the animals (except for the untreated animals used for blood collection only) were euthanized in a CO_2_ chamber.

#### 3.2.2. Study of the Antiplatelet Activity In Vitro

The antiplatelet activity of the compounds was determined with a 220LA two-channel laser platelet aggregation analyzer (Biola, Moscow, Russia) using an in vitro model of ADP-induced platelet aggregation, according to a previously described protocol [20]. The activity of the compounds was assessed through the degree of platelet aggregation after the incubation of the platelet-rich plasma with the test compounds at the final concentrations of 1 × 10^−5^, 1 × 10^−6^, 1 × 10^−7^, and 1 × 10^−8^ M. Acetylsalicylic acid (ASA; Sigma-Aldrich, St. Louis, MO, USA) was used as a reference drug [24]. The tested compounds and ASA were dissolved in distilled water (30 µL). Distilled water in a similar volume was used as a control. The blood was collected from the rats with a puncture of their hypoglossal vein [25]. Next, the blood was centrifuged for 15 min at 1000 rpm on a SM-6M centrifuge (Elmi, Riga, Latvia). After centrifugation, the platelet-rich plasma was immediately used. ADP (Sigma-Aldrich, St. Louis, MO, USA) was used as an aggregation inducer at a final concentration of 5 µM. The antiplatelet activity was evaluated with a degree of aggregation defined as the maximum increment of light transmission after the addition of the inducer.

#### 3.2.3. Study of the Antithrombotic Activity In Vivo

The study of the antithrombotic activity of the compounds was carried out in vivo on a model of arterial thrombosis induced by applying 25 µL of a 50% FeCl_3_ solution to the carotid artery of the rats, according to the method described in [26], 24 h and 1 h after the intraperitoneal administration of the compounds at their most active equimolar doses (1/10 MW). Zoletil and xylazine were used for anesthesia. The study was carried out using a Doppler ultrasound scanner (Mini-Max Doppler, St. Petersburg, Russia); the time until the complete occlusion of the vessel was recorded.

The ligation of the inferior vena cava was used as a model for deep vein thrombosis according to the method described in [27]. In the rats under general anesthesia (zoletil and xylazine), the inferior vena cava was ligated 1 cm above the bifurcation site; the resulting thrombus was removed and weighed after 24 h. The test compounds at a dose of 1/10 MW (mg/kg) or the reference drug were administered 1 h before the ligation of the inferior vena cava.

The bleeding time was assessed in the adult male mice (20–22 g) after the intragastric administration of the test compounds. A scalpel was used to transect the tail tip (5 mm). The bleeding tail stump was immersed in normal saline heated to 37 °C, and the time to stop the bleeding was recorded [28]. The test compounds at their most active equimolar doses (1/10 MW) or the reference drug were administered 24 h and 1 h before the experiment.

#### 3.2.4. Study of the Anticoagulant Activity In Vivo

The thrombin time (TT), prothrombin time (PT), activated partial thromboplastin time (aPTT), and fibrinogen content were determined chronometrically with an APG2-01 MINILAB 701 hemostasis analyzer (Unimed, Moscow, Russia) using reagent kits (Renam, Moscow, Russia). The test compounds at a dose of 1/10 MW (mg/kg) were administered intraperitoneally once a day for 3 days, after which the blood was collected from the hypoglossal vein. All the coagulation tests were performed on platelet-poor plasma, stabilized with a 3.8% sodium citrate solution in a ratio of 9:1, followed by centrifugation at 3000 rpm for 15 min, according to the method described in [28].

#### 3.2.5. Study of Acute Toxicity

The acute toxicity of their most active compounds was assessed using the outbred mice. The mortality of the animals was observed over a two-week period after the intragastric administration of the compounds, followed by calculation of the LD_50_.

#### 3.2.6. Statistical Analysis

Statistical data processing was carried out using the GraphPad Prism 6 (GraphPad Software, San Diego, CA, USA) and Microsoft Office Excel 2016 software packages. The normality of data distribution was tested using the Shapiro–Wilk test. A one-way ANOVA with Bonferroni’s multiple comparisons test or a Kruskal–Wallis test with Dunn’s multiple comparisons test was employed to compare the experimental groups with the control group. The data were presented as the mean ± standard error of the mean (M ± SEM). The statistical significance was set to *p* < 0.05.

## 4. Conclusions

In summary, the study of the antiplatelet and antithrombotic activity of the newly obtained amides and peptides of (2*S*,4*S*)-4-amino-1-phenyl-, (2*S*,4*S*)-4-amino-1-(4-fluorophenyl)-, and (2*S*,4*S*)-4-[(methyl)(phenyl)amino]-5-oxoprolines made it possible to identify a number of compounds that exhibit antiplatelet activity in vitro and in vivo. It was found that these compounds slow down the process of thrombus formation in models of arterial and venous thrombosis, without affecting plasma hemostasis parameters. (2*S*,4*S*)-4-Amino-1-(4-fluorophenyl)-5-oxoprolyl-(*S*)-phenylalanine (**9b**) proved to be the most efficient among the studied derivatives. The results obtained indicate the advisability of further studies of 5-oxoproline derivatives in order to design pharmaceutical agents for the prevention and treatment of the consequences of thrombosis (cerebrovascular accident, myocardial infarction, etc.).

## Figures and Tables

**Table 1:** Antiplatelet activity of the test compounds in ADP-induced platelet aggregation in vitro (*M* ± *SEM*, *n* = 8).

Compound	MW	Platelet Aggregation (Δ%)			
		10^−5^ M	10^−6^ M	10^−7^ M	10^−8^ M
Control (H_2_O)		7.4 ± 0.2			
**1b**	274	4.6 ± 0.9 *	5.2 ± 0.5 *	5.2 ± 0.7 *	5.7 ± 1.1
**5a**	291	6.4 ± 0.9	7.0 ± 1.0	7.3 ± 0.9	7.8 ± 0.9
**5b**	323	7.3 ± 1.0	7.5 ± 1.2	7.4 ± 0.9	8.0 ± 1.2
**6a**	289	6.3 ± 0.7	7.2 ± 0.3	7.3 ± 0.2	7.4 ± 0.4
**6b**	316	4.9 ± 0.8 *	5.1 ± 0.6 *	4.8 ± 1.0 *	5.3 ± 1.1 *
**9a**	376	4.3 ± 0.2 *	4.6 ± 0.2 *	5.1 ± 0.3 *	5.5 ± 0.5 *
**9b**	385	4.2 ± 0.8 *	4.5 ± 0.7 *	5.2 ± 1.0 *	5.6 ± 0.6 *
**10a**	365	6.3 ± 0.7	6.1 ± 0.8	6.6 ± 1.0	6.8 ± 1.0
**10b**	389	5.4 ± 0.6 *	6.2 ± 0.7	5.7 ± 1.3	5.6 ± 1.2
**12**	301	6.3 ± 0.7	6.8 ± 0.4	7.3 ± 0.5	7.3 ± 0.4
**13**	303	4.4 ± 0.7 *	5.0 ± 0.4 *	4.9 ± 0.6 *	5.3 ± 0.8 *
**16**	381	6.3 ± 1.2	6.0 ± 1.2	7.0 ± 0.9	7.6 ± 1.0
ASA	180	4.7 ± 0.2 *	5.4 ± 0.4 *	6.0 ± 0.5	6.5 ± 1.0

The data for compound **1b** [15] are given for comparison. * Differences are statistically significant relative to the control group (H_2_O), *p* < 0.05.

**Table 2:** In vivo antithrombotic activity of the tested compounds (M ± SEM; in the control group *n* = 15; in other groups, *n* = 8).

Compound	Duration of Thrombus Formation in the Carotid Artery during the FeCl_3_ Application (min)	Weight of Venous Thrombus during Ligation of the Inferior Vena Cava (mg)	Duration of Bleeding When Cutting off the Tip of the Tail (min)
Control (water)	19.4 ± 1.4	77.8 ± 5.7	5.5 ± 0.6
**1b**	37.0 ± 5.8 *	42.0 ± 16.7 *	8.5 ± 1.9 *
**5a**	21.2 ± 4.7	73.3 ± 3.4	6.0 ± 1.1
**5b**	23.5 ± 3.6	66.3 ± 13.1	6.4 ± 1.4
**6a**	22.8 ± 3.8	68.3 ± 10.9	6.0 ± 1.2
**6b**	28.3 ± 4.6 *	55.6 ± 8.5 *	8.5 ± 1.1 *
**9a**	30.8 ± 6.3 *	41.8 ± 16.2 *	8.6 ± 0.9 *
**9b**	33.0 ± 3.0 *	33.8 ± 11.6 *	10.1 ± 1.6 *
**10a**	27.5 ± 5.1	57.5 ± 8.2 *	7.8 ± 1.3
**10b**	23.0 ± 2.1	66.5 ± 9.7	8.3 ± 0.8 *
**12**	23.9 ± 4.0	71.8 ± 9.1	6.2 ± 1.2
**13**	29.6 ± 5.6 *	58.3 ± 13.6 *	8.5 ± 1.9 *
**16**	23.5 ± 1.7	68.3 ± 10.9	6.2 ± 1.1
ASA	43.0 ± 4.4 *	43.6 ± 9.5 *	7.8 ± 1.0 *

The data for compound **1b** [15] are given for comparison. * Differences are statistically significant relative to the control group (H_2_O), *p* < 0.05.

**Table 3:** Effect of the studied compounds on the parameters of the coagulogram of rat blood in vivo, at a concentration of 1/10 MW in mg/kg (M ± SEM; in the control group *n* = 10; in other groups *n* = 8). aPTT, activated partial thromboplastin time; PT, prothrombin time; and TT, thrombin time.

Compound	aPTT (s)	PT (s)	TT (s)	Fibrinogen (g/L)
Control (H_2_O)	22.4 ± 1.5	14.1 ± 1.3	24.6 ± 1.2	4.4 ± 0.1
**1b**	24.2 ± 3.4	15.8 ± 2.7	29.8 ± 3.7	4.3 ± 0.3
**5a**	21.8 ± 1.0	17.4 ± 0.1	28.5 ± 5.0	4.9 ± 0.4
**5b**	24.2 ± 1.4	16.1 ± 2.9	29.7 ± 2.8	4.7 ± 0.2
**6a**	24.4 ± 2.4	16.9 ± 0.5	29.6 ± 1.7	4.4 ± 0.3
**6b**	23.7 ± 3.8	15.7 ± 1.0	30.5 ± 1.5	4.1 ± 0.4
**9a**	24.3 ± 3.7	13.1 ± 2.9	25.3 ± 4.3	4.2 ± 0.2
**9b**	25.5 ± 4.0	15.7 ± 2.7	27.0 ± 1.0	4.3 ± 0.3
**10a**	23.8 ± 2.6	14.4 ± 1.6	24.5 ± 3.0	4.6 ± 0.4
**10b**	25.4 ± 2.7	13.8 ± 1.6	22.3 ± 4.8	4.3 ± 0.4
**12**	25.5 ± 4.0	15.5 ± 1.9	28.5 ± 0.4	4.3 ± 0.3
**13**	25.3 ± 4.6	16.1 ± 1.4	30.8 ± 2.7	4.7 ± 0.2
**16**	25.5 ± 2.8	16.7 ± 3.1	24.5 ± 2.1	4.9 ± 0.3
ASA	25.9 ± 1.6	11.6 ± 1.2	22.4 ± 3.0	4.2 ± 0.2

Differences are statistically significant relative to the control group (H_2_O), *p* < 0.05.

## Data Availability

Data are contained within the article.

## Supplementary Materials

The following supporting information can be downloaded at: https://www.mdpi.com/article/10.3390/molecules28217401/s1, Figures S1–S61: ^1^H, ^19^F, and ^13^C NMR spectra of compounds **2b**, **3–10a,b**, and **12–16**; 2D ^1^H–^13^C HSQC and HMBC NMR spectra of compounds **8a**, **10a**, and **14**; 2D ^1^H–^1^H NOESY spectra of compounds **10a** and **14**; Table S1: Selected X-ray single-crystal data and structure refinement details of compound **14**.
