# Supplementary material for: Synthesis and Assessment of Antiplatelet and Antithrombotic Activity of 4-Amino-Substituted 5-Oxoproline Amides and Peptides
Source: Molecules. 2023 Nov 2;28(21):7401. doi: 10.3390/molecules28217401 (PMC10648734; doi:10.3390/molecules28217401)
Supplement: Supplementary file 1 [file molecules-28-07401-s001.zip › molecules-2699031-supplementary.pdf]

Supplementary Materials

Synthesis and Assessment of Antiplatelet and Antithrombotic Activity of 4-Substituted 5-Oxoproline Amides and Peptides

Victor P. Krasnov [1,*], Irina A. Nizova [1], Alexey Yu. Vigorov [1], Tatyana V. Matveeva [1], Galina L. Levit [1], Mikhail I. Kodess [1], Marina A. Ezhikova [1], Pavel A. Slepukhin [1], Dmitry A. Bakulin [2], Ivan N. Tyurenkov [2], Valery N. Charushin [1,3]

[1] Postovsky Institute of Organic Synthesis, Russian Academy of Sciences (Ural Branch), Ekaterinburg 620108, Russia

[2] Volgograd State Medical University, Volgograd 400131, Russia

[3] Chemical Engineering Institute, Ural Federal University, Ekaterinburg 620002, Russia

Table of Contents

NMR Spectra

Figure S1. ^{1}H NMR spectrum of **2b** S-3
Figure S2. ^{13}C NMR spectrum of **2b** S-3
Figure S3. ^{19}F NMR spectrum of **2b** S-4
Figure S4. ^{1}H NMR spectrum of **3a** S-4
Figure S5. ^{13}C NMR spectrum of **3a** S-5
Figure S6. ^{1}H NMR spectrum of **3b** S-5
Figure S7. ^{13}C NMR spectrum of **3b** S-6
Figure S8. ^{19}F NMR spectrum of **3b** S-6
Figure S9. ^{1}H NMR spectrum of **4a** S-7
Figure S10. ^{13}C NMR spectrum of **4a** S-7
Figure S11. ^{1}H NMR spectrum of **4b** S-8
Figure S12. ^{13}C NMR spectrum of **4b** S-8
Figure S13. ^{19}F NMR spectrum of **4b** S-9
Figure S14. ^{1}H NMR spectrum of **5a** S-9
Figure S15. ^{13}C NMR spectrum of **5a** S-10
Figure S16. ^{1}H NMR spectrum of **5b** S-10
Figure S17. ^{13}C NMR spectrum of **5b** S-11
Figure S18. ^{19}F NMR spectrum of **5b** S-11
Figure S19. ^{1}H NMR spectrum of **6a** S-12
Figure S20. ^{13}C NMR spectrum of **6a** S-12
Figure S21. ^{1}H NMR spectrum of **6b** S-13
Figure S22. ^{13}C NMR spectrum of **6b** S-13
Figure S23. ^{19}F NMR spectrum of **6b** S-14

Figure S24. ^{1}H NMR spectrum of **7a** S-14
Figure S25. ^{13}C NMR spectrum of **7a** S-15
Figure S26. ^{1}H NMR spectrum of **7b** S-15
Figure S27. ^{13}C NMR spectrum of **7b** S-16
Figure S28. ^{19}F NMR spectrum of **7b** S-16
Figure S29. ^{1}H NMR spectrum of **8a** S-17
Figure S30. ^{13}C NMR spectrum of **8a** S-17
Figure S31. 2D ^{1}H-^{13}C HSQC spectrum of **8a** S-18
Figure S32. 2D ^{1}H-^{13}C HMBC spectrum of **8a** S-18
Figure S33. ^{1}H NMR spectrum of **8b** S-19
Figure S34. ^{13}C NMR spectrum of **8b** S-19
Figure S35. ^{19}F NMR spectrum of **8b** S-20
Figure S36. ^{1}H NMR spectrum of **9a** S-20
Figure S37. ^{13}C NMR spectrum of **9a** S-21
Figure S38. ^{1}H NMR spectrum of **9b** S-21
Figure S39. ^{13}C NMR spectrum of **9b** S-22
Figure S40. ^{19}F NMR spectrum of **9b** S-22
Figure S41. ^{1}H NMR spectrum of **10a** S-23
Figure S42. ^{13}C NMR spectrum of **10a** S-23
Figure S43. 2D ^{1}H-^{13}C HSQC spectrum of **10a** S-24
Figure S44. 2D ^{1}H-^{13}C HMBC spectrum of **10a** S-24
Figure S45. 2D ^{1}H-^{1}H NOESY spectrum of **10a** S-25
Figure S46. ^{1}H NMR spectrum of **10b** S-25
Figure S47. ^{13}C NMR spectrum of **10b** S-26
Figure S48. ^{19}F NMR spectrum of **10b** S-26
Figure S49. ^{1}H NMR spectrum of **12** S-27
Figure S50. ^{13}C NMR spectrum of **12** S-27
Figure S51. ^{1}H NMR spectrum of **13** S-28
Figure S52. ^{13}C NMR spectrum of **13** S-28
Figure S53. ^{1}H NMR spectrum of **14** S-29
Figure S54. ^{13}C NMR spectrum of **14** S-29
Figure S55. 2D ^{1}H-^{13}C HSQC spectrum of **14** S-30
Figure S56. 2D ^{1}H-^{13}C HMBC spectrum of **14** S-30
Figure S57. 2D ^{1}H-^{1}H NOESY spectrum of **14** S-31
Figure S58. ^{1}H NMR spectrum of **15** S-31
Figure S59. ^{13}C NMR spectrum of **15** S-32
Figure S60. ^{1}H NMR spectrum of **16** S-32
Figure S61. ^{13}C NMR spectrum of **16** S-33

X-Ray Diffraction Data

Table S1. Selected X-ray single-crystal data and structure refinement details of compound **14** S-34

NMR Spectra

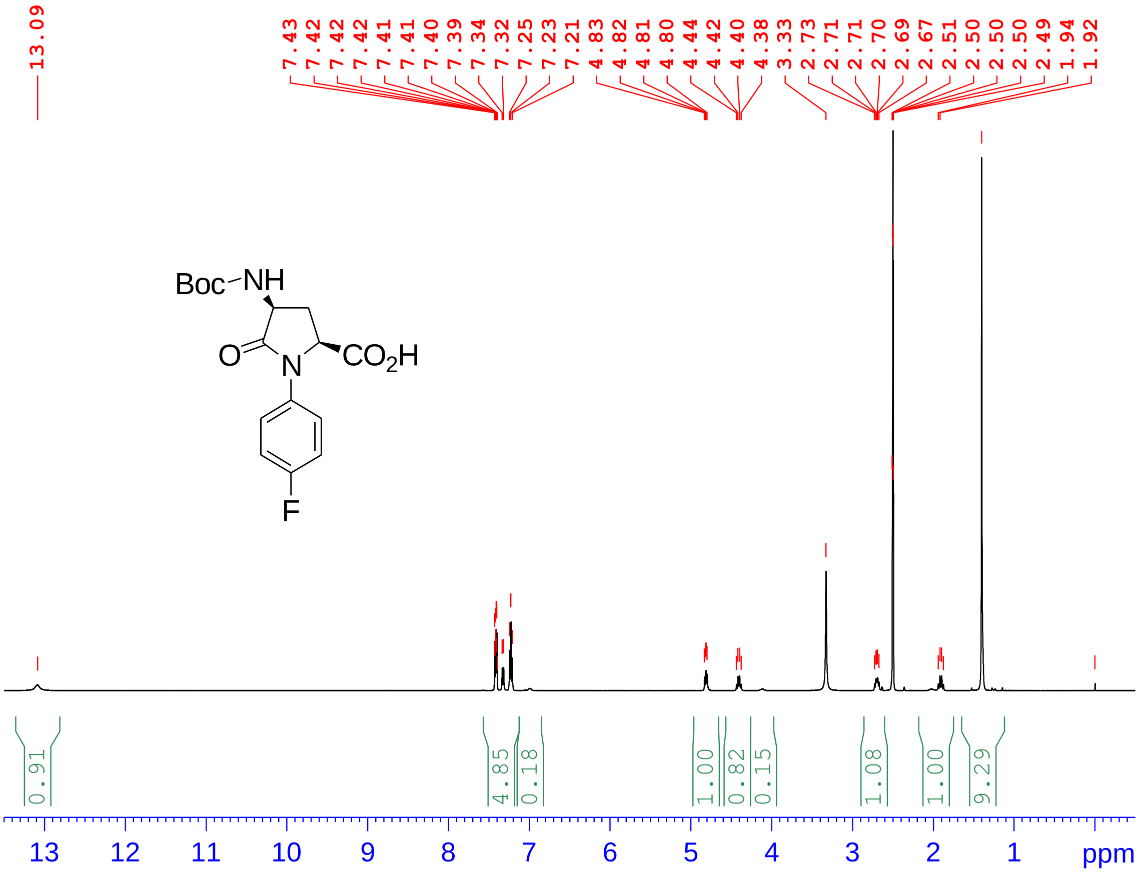

Figure S1. ^{1}H NMR (500 MHz, DMSO-d_6) spectrum of **2b**.

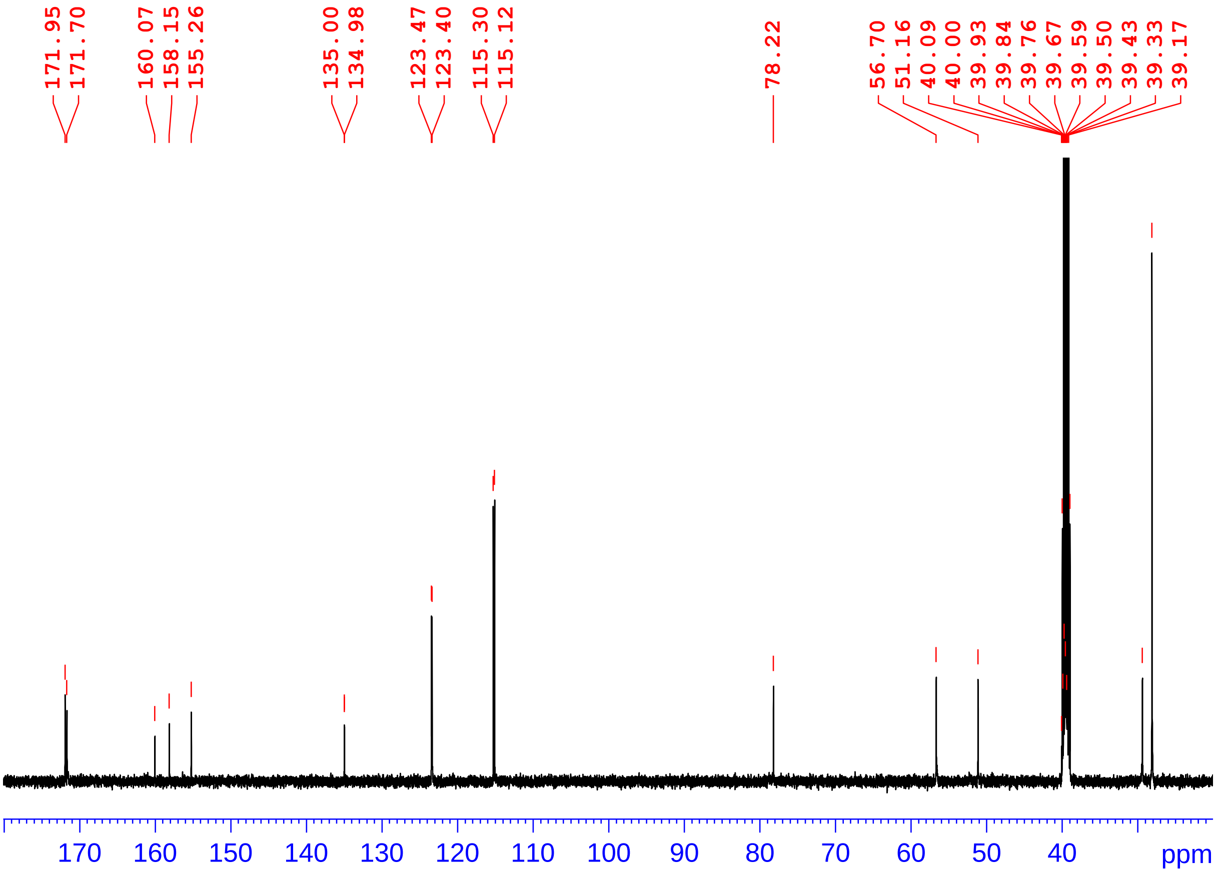

Figure S2. ^{13}C NMR (126 MHz, DMSO-d_6) spectrum of **2b**.

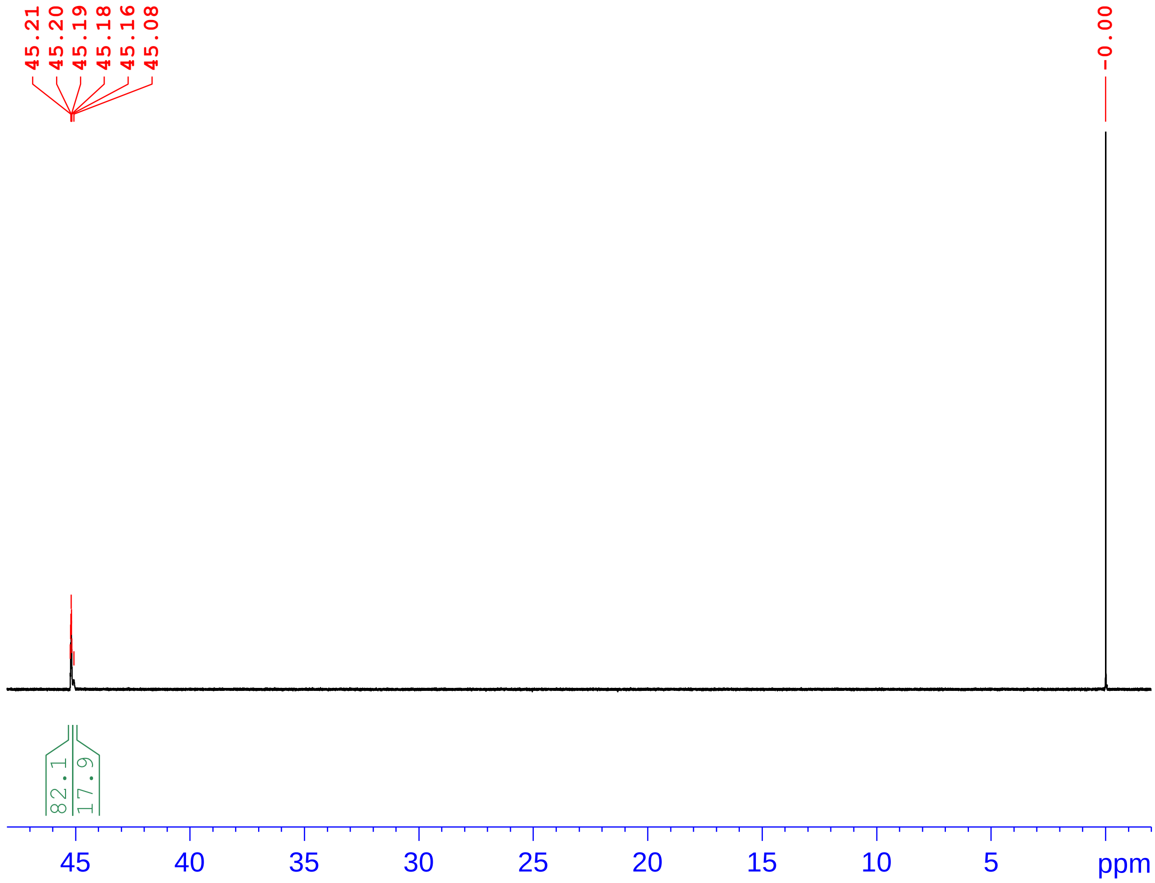

Figure S3. ^{19}F NMR (376 MHz, DMSO-d_6) spectrum of **2b**.

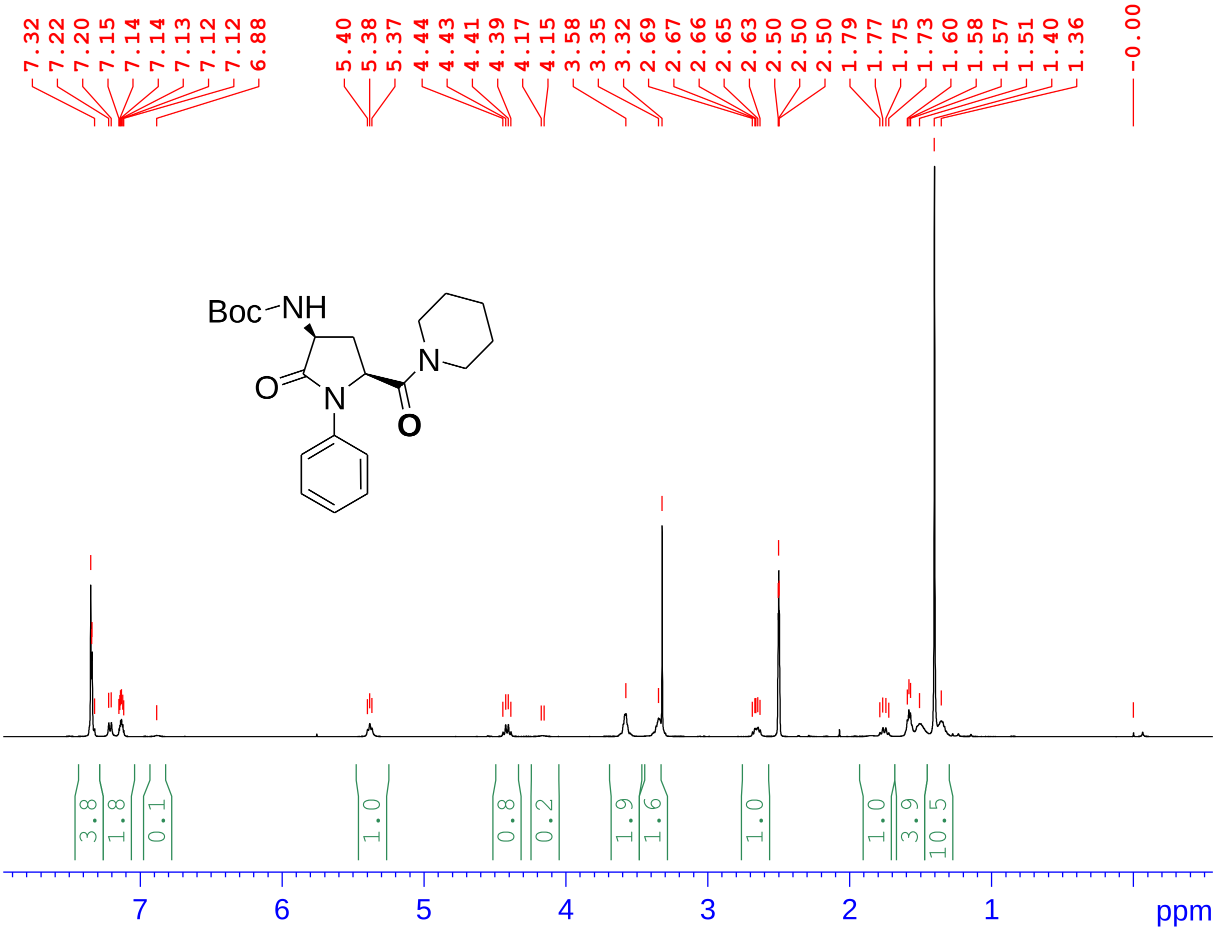

Figure S4. ^{1}H NMR (500 MHz, DMSO-d_6) spectrum of **3a**.

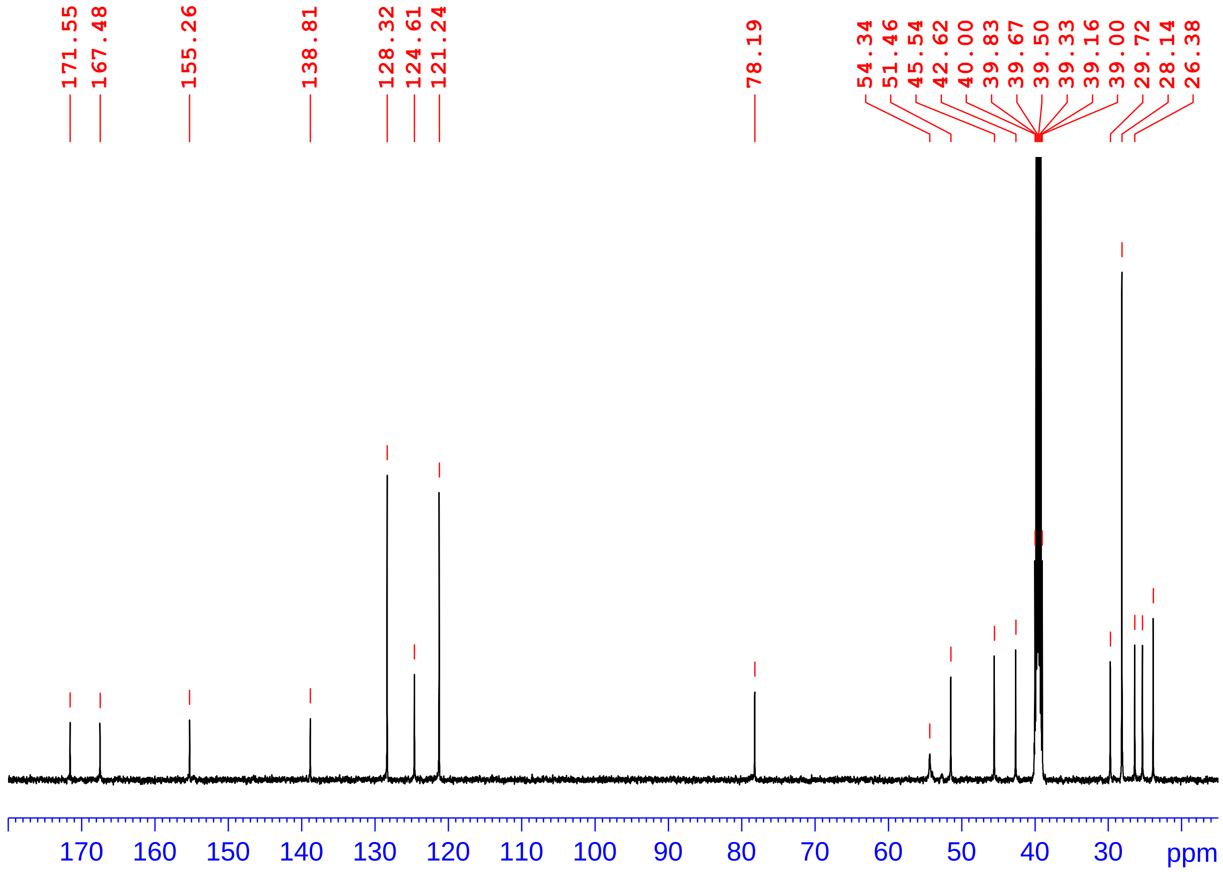

Figure S5. ^{13}C NMR (126 MHz, DMSO-d_6) spectrum of **3a**.

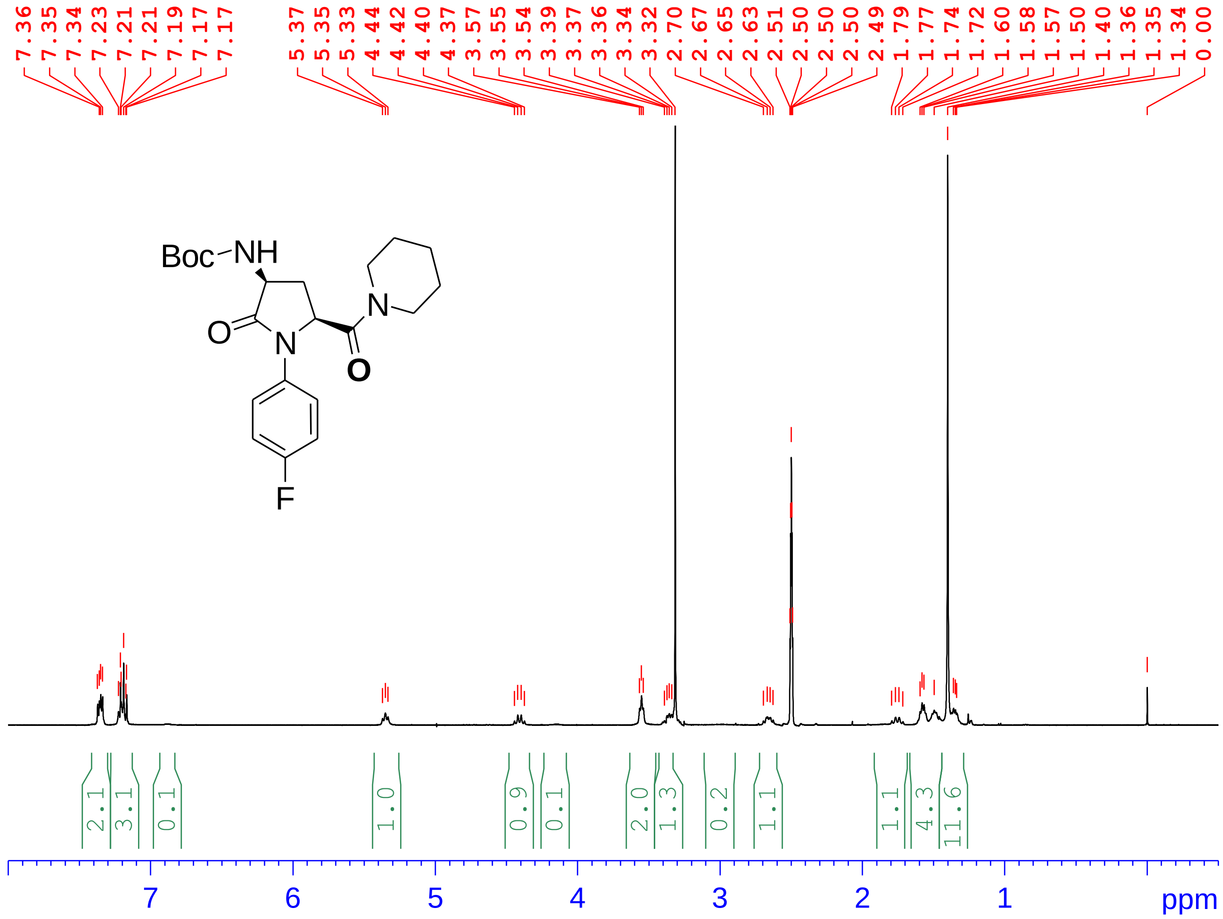

Figure S6. ^{1}H NMR (400 MHz, DMSO-d_6) spectrum of **3b**.

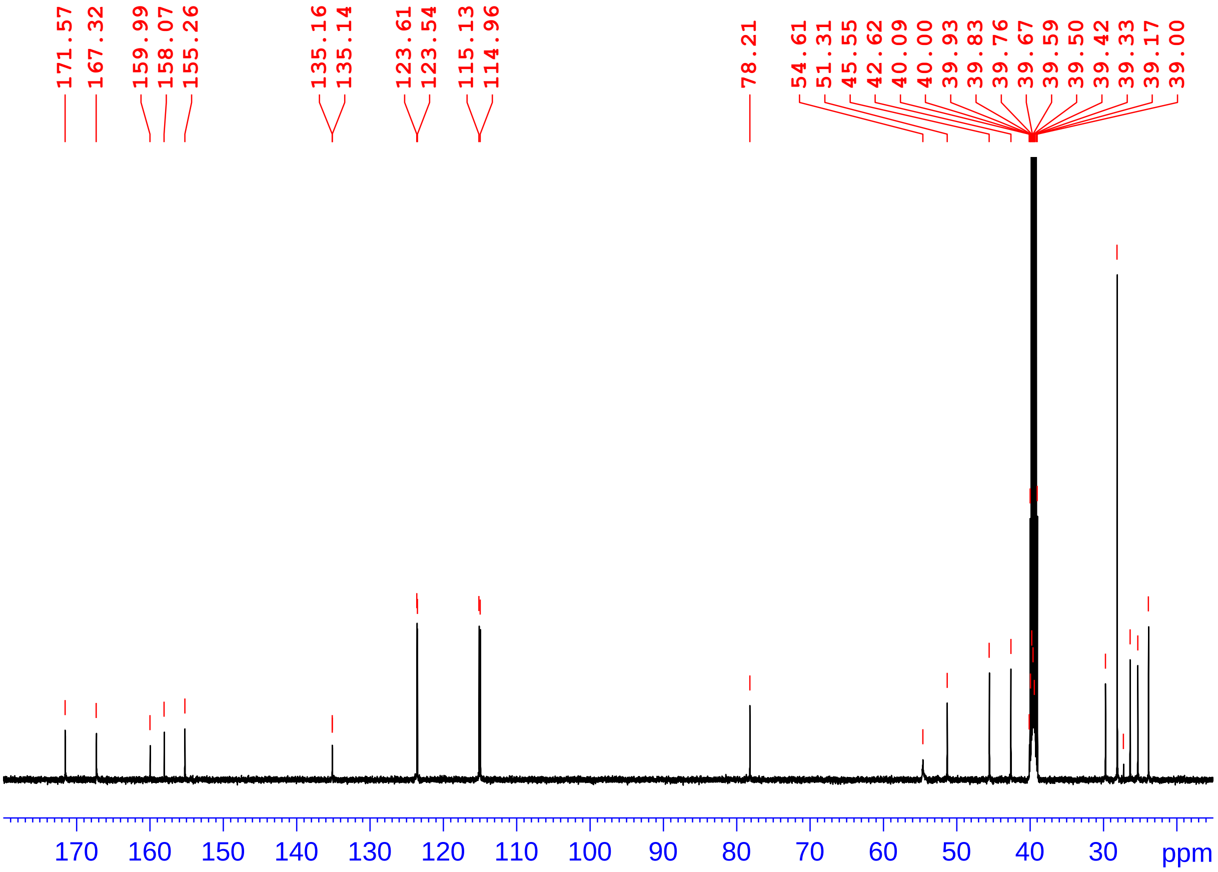

Figure S7. ^{13}C NMR (126 MHz, DMSO-d_6) spectrum of **3b**.

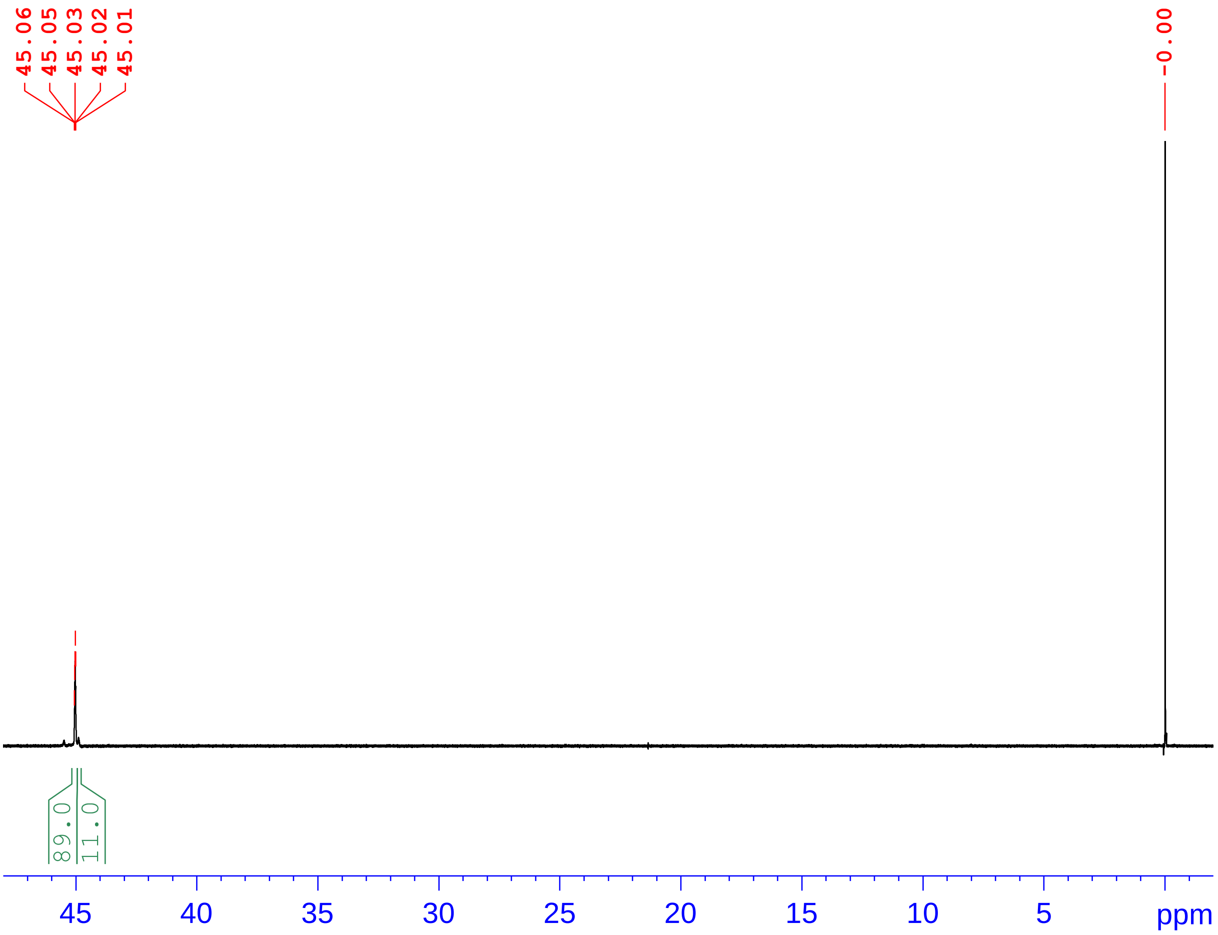

Figure S8. ^{19}F NMR (376 MHz, DMSO-d_6) spectrum of **3b**.

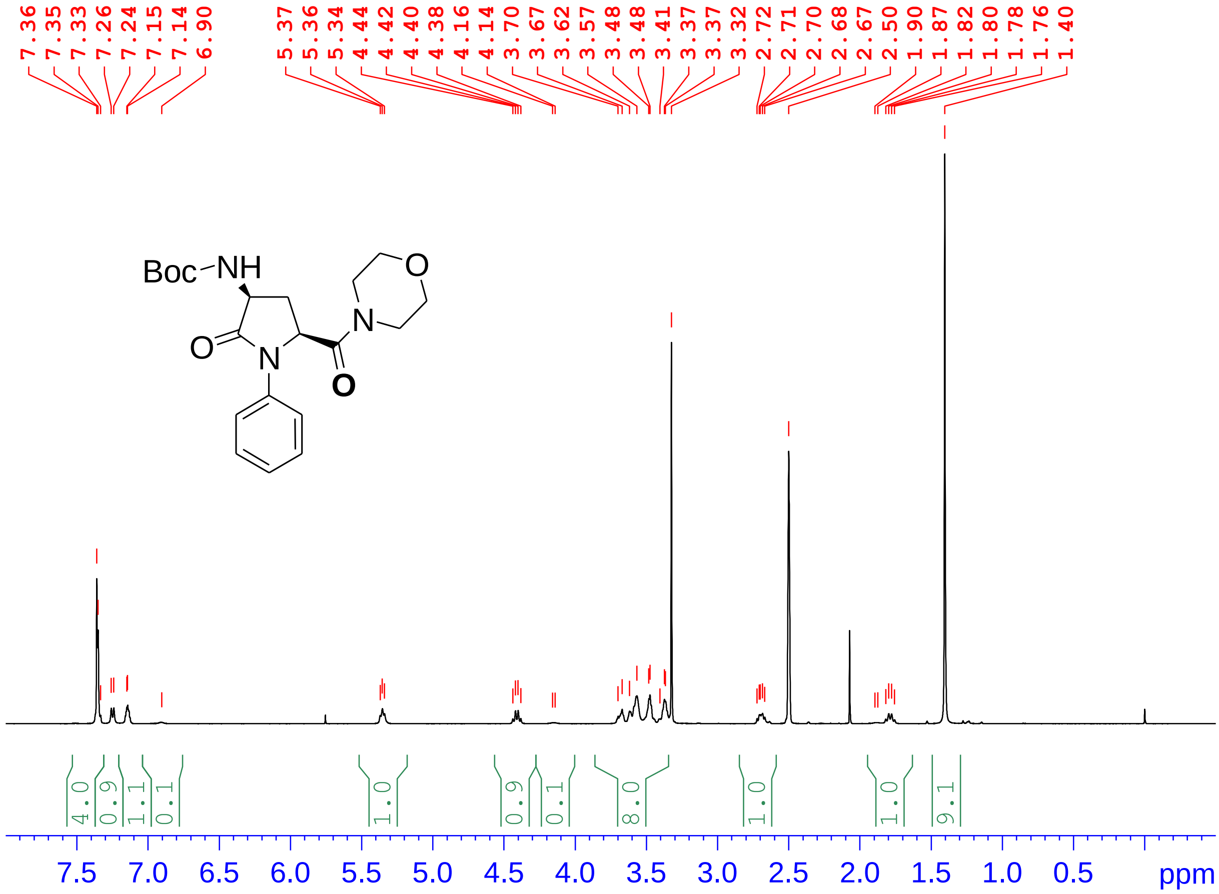

Figure S9. ^{1}H NMR (500 MHz, DMSO-d_6) spectrum of **4a**.

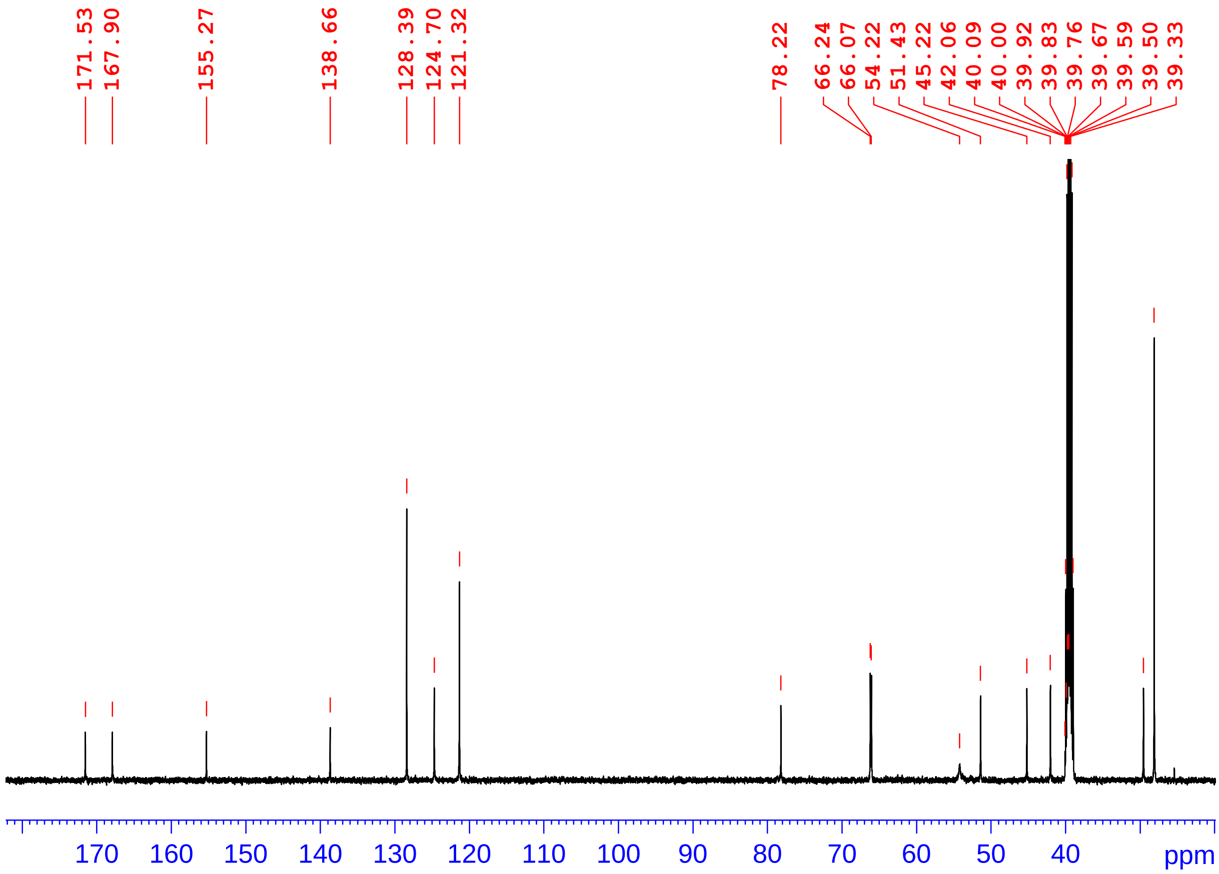

Figure S10. ^{13}C NMR (126 MHz, DMSO-d_6) spectrum of **4a**.

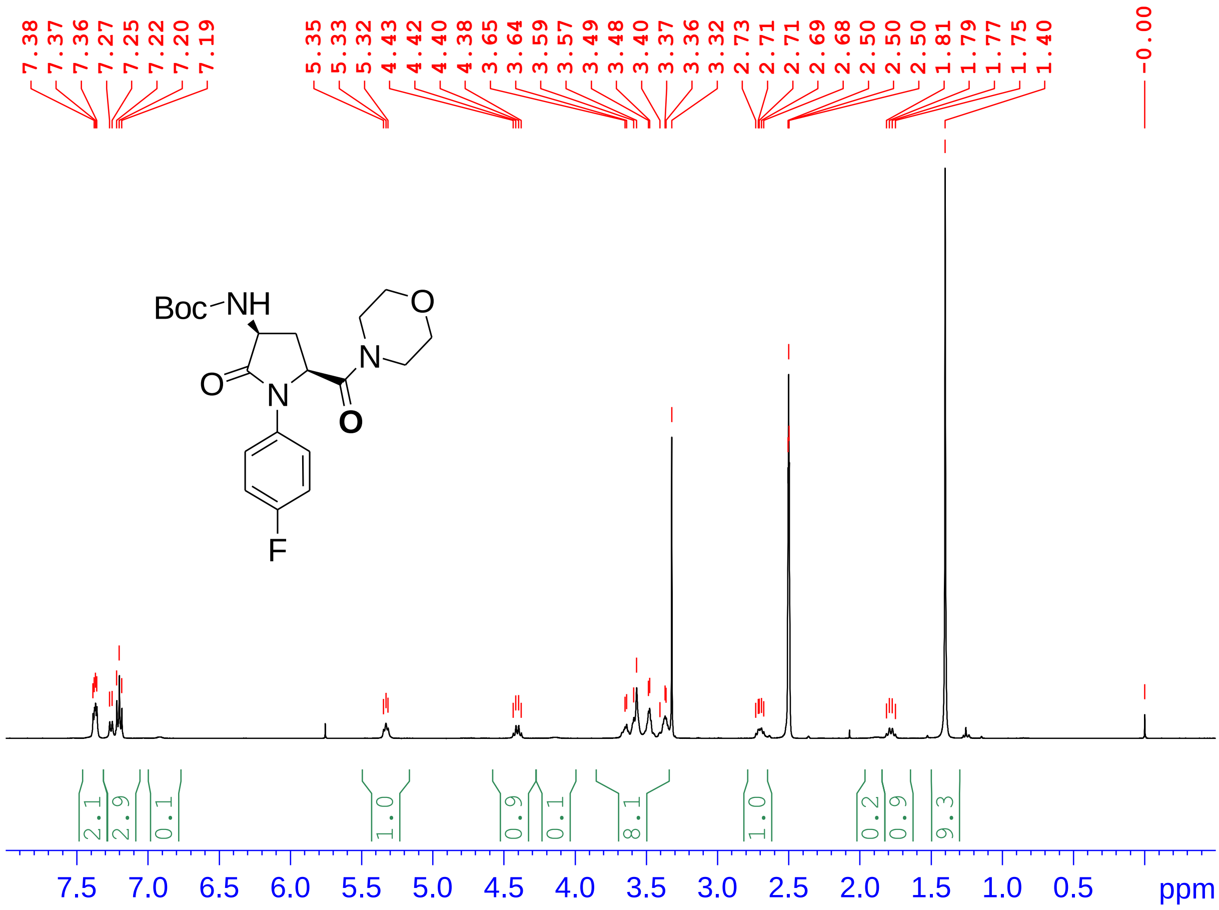

Figure S11. ^{1}H NMR (500 MHz, DMSO-d_6) spectrum of **4b**.

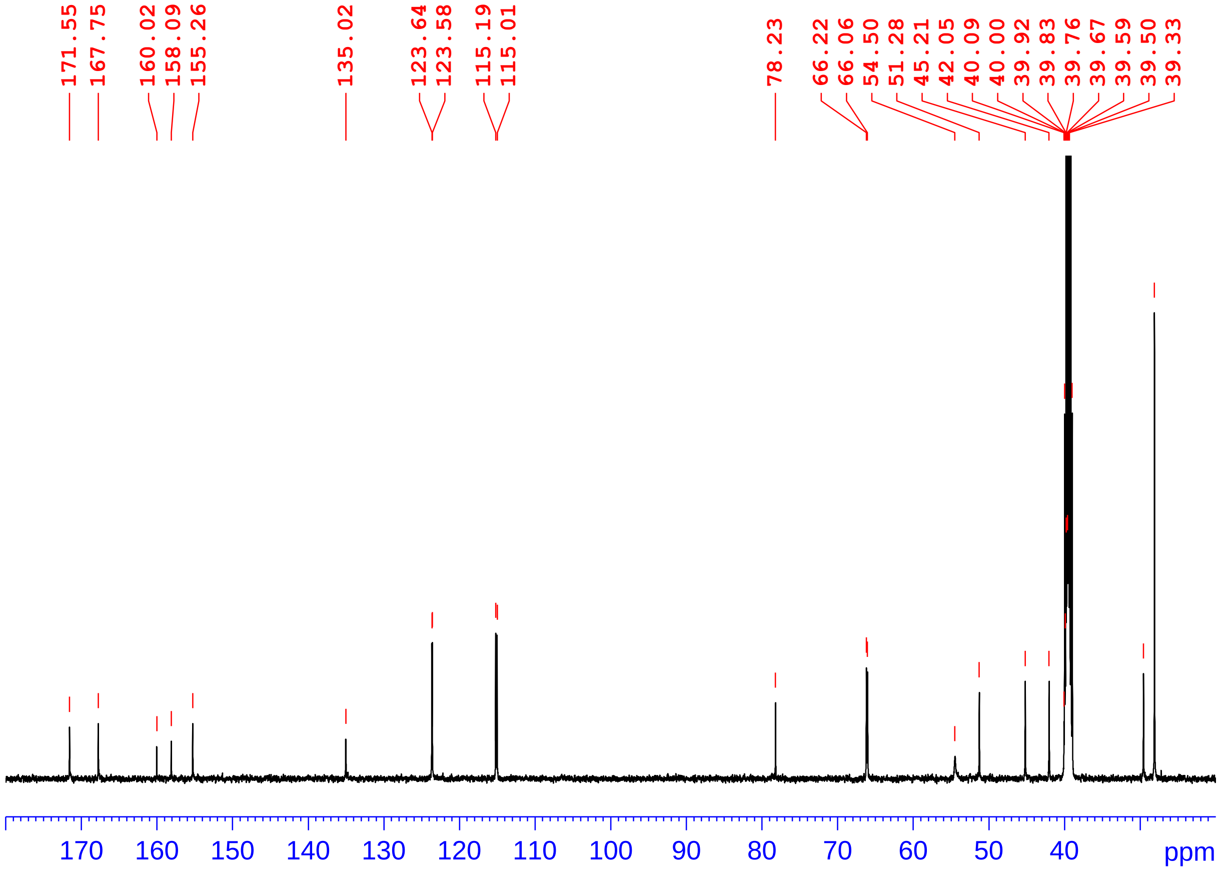

Figure S12. ^{13}C NMR (126 MHz, DMSO-d_6) spectrum of **4b**.

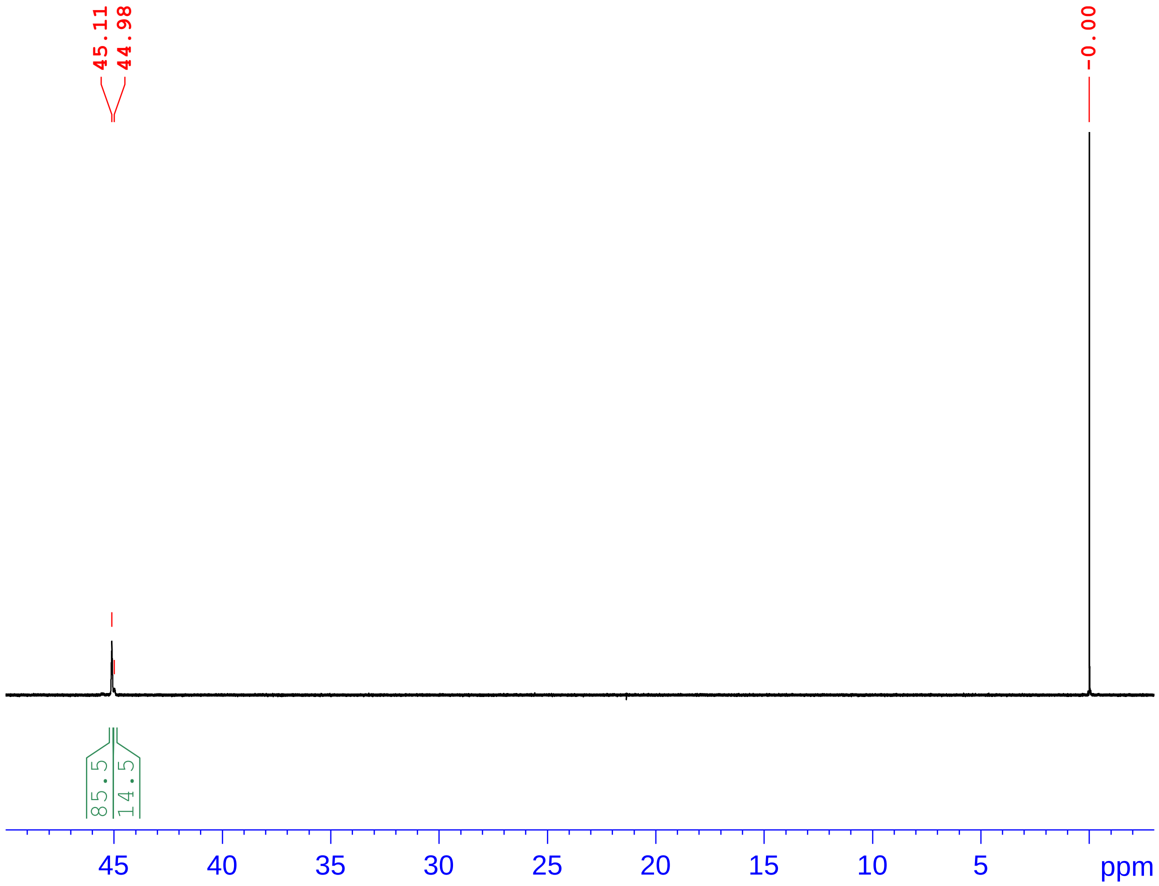

Figure S13. ^{19}F NMR (376 MHz, DMSO-d_6) spectrum of **4b**.

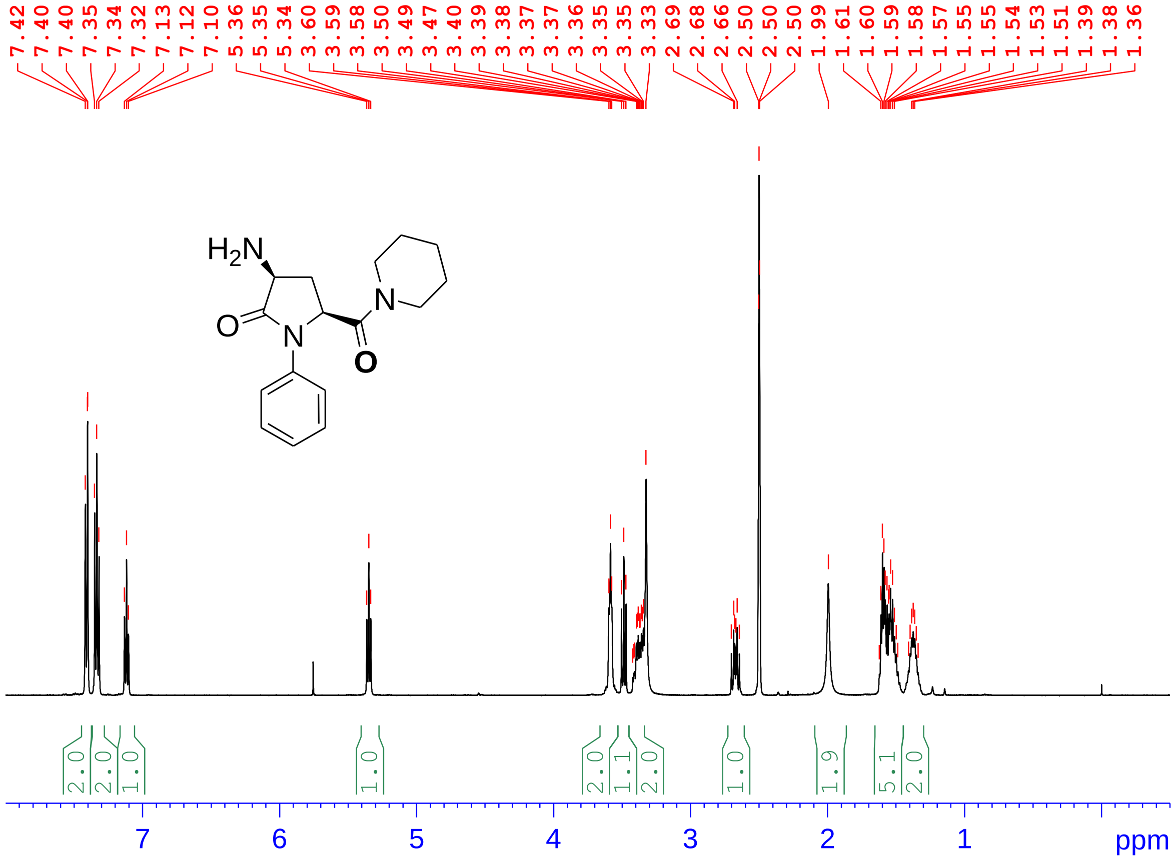

Figure S14. ^{1}H NMR (500 MHz, DMSO-d_6) spectrum of **5a**.

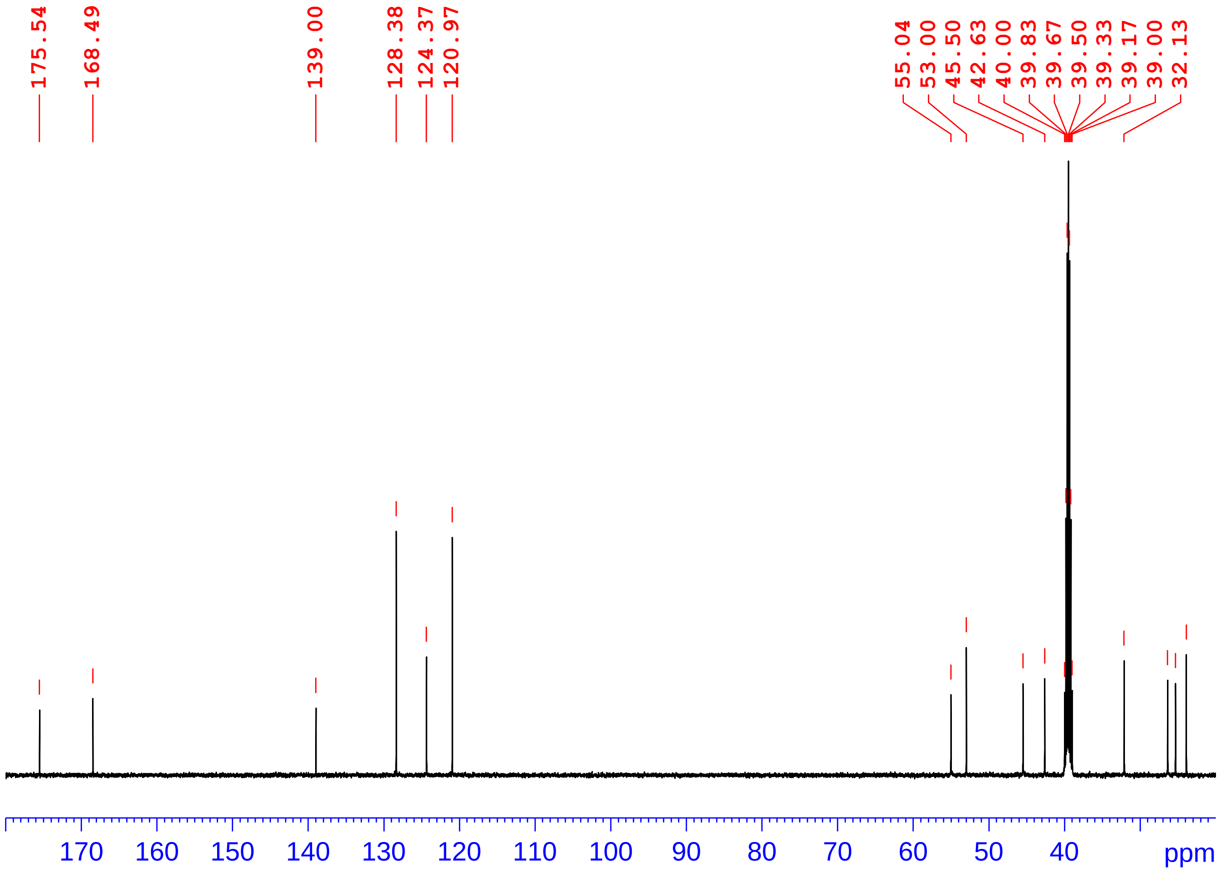

Figure S15. ^{13}C NMR (126 MHz, DMSO-d_6) spectrum of **5a**.

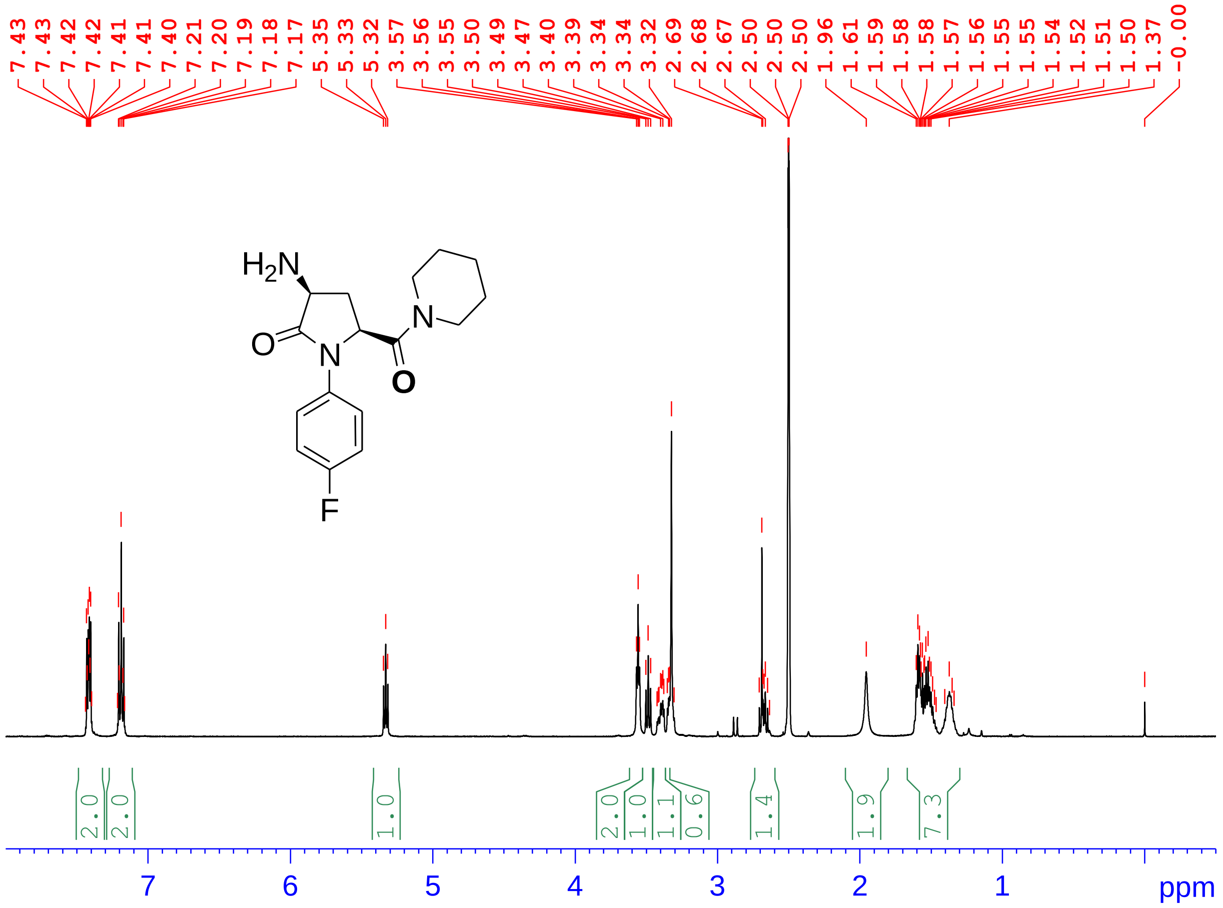

Figure S16. ^{1}H NMR (500 MHz, DMSO-d_6) spectrum of **5b**.

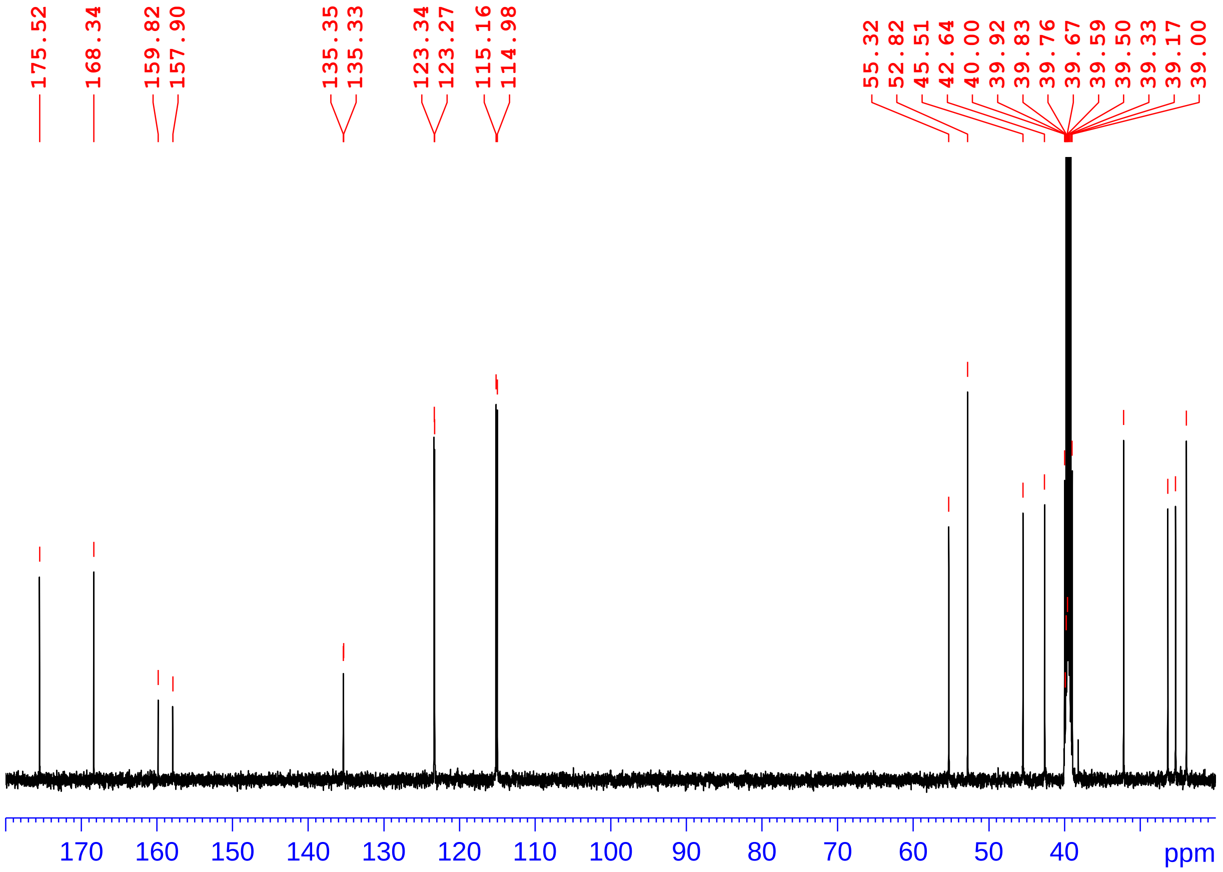

Figure S17. ^{13}C NMR (126 MHz, DMSO-d_6) spectrum of **5b**.

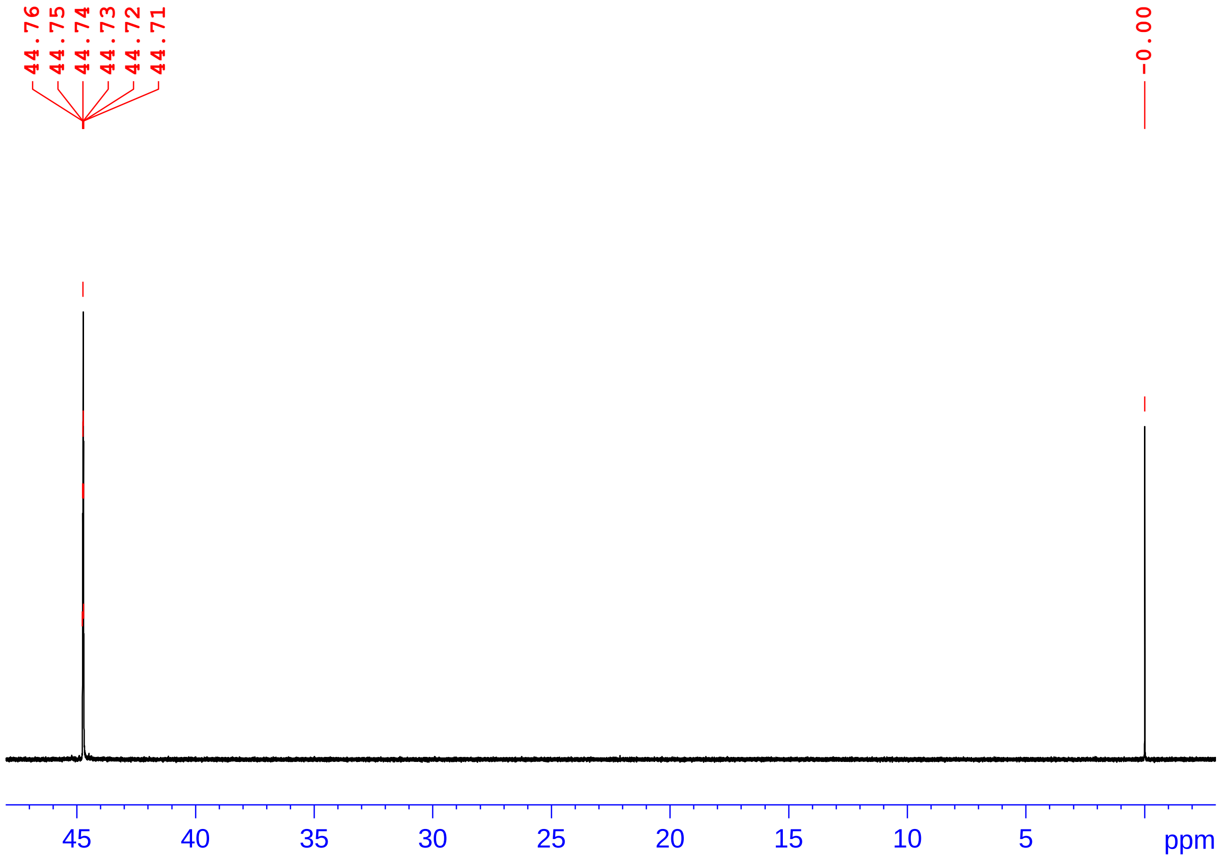

Figure S18. ^{19}F NMR (470 MHz, DMSO-d_6) spectrum of **5b**.

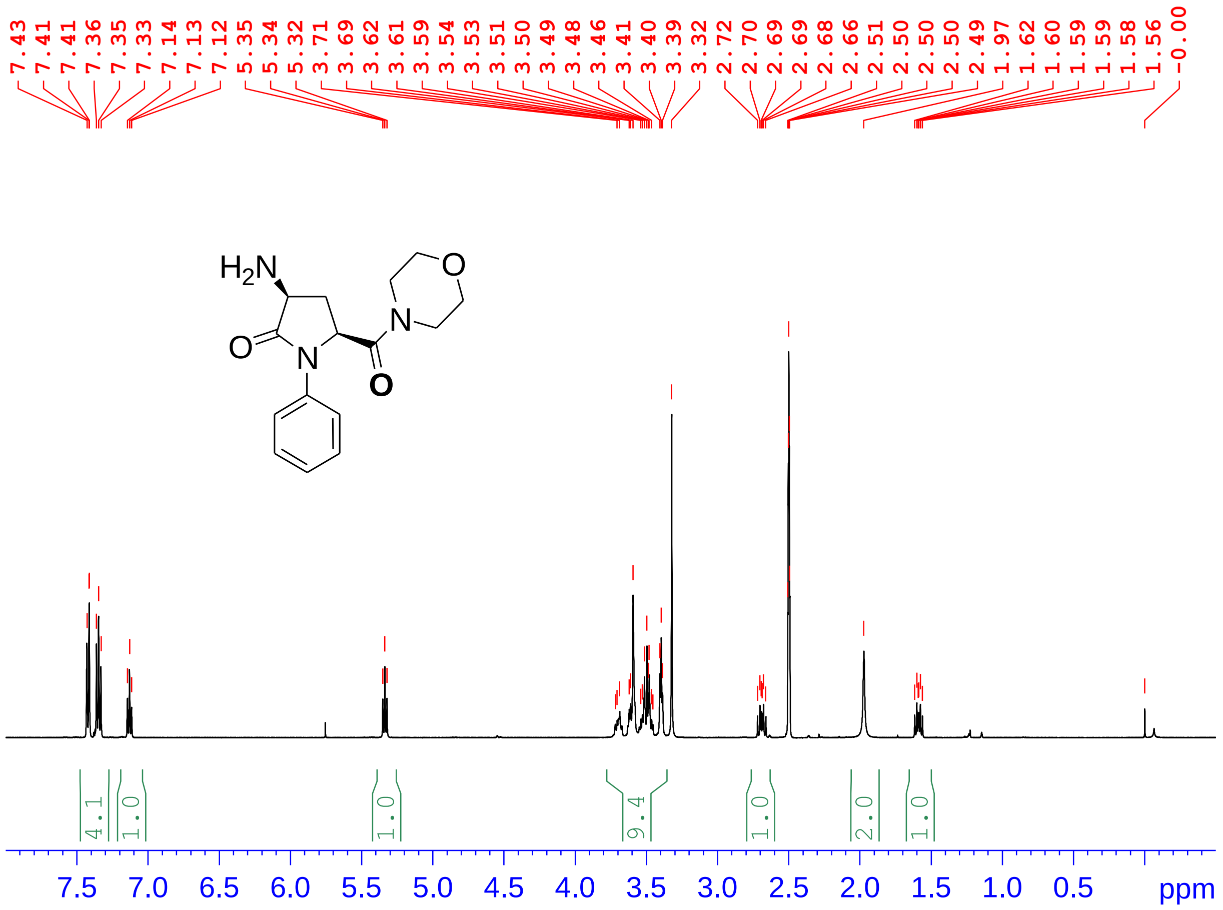

Figure S19. ^{1}H NMR (500 MHz, DMSO-d_6) spectrum of **6a**.

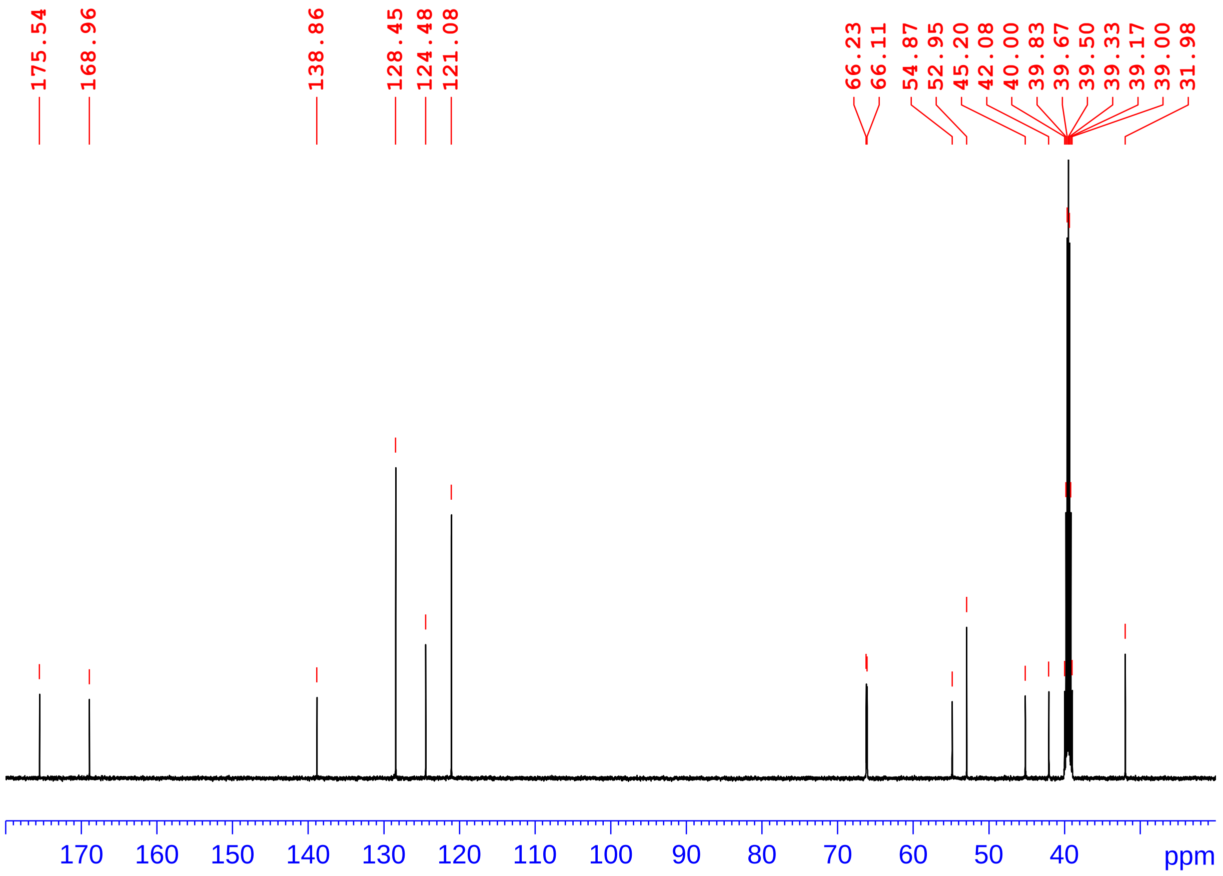

Figure S20. ^{13}C NMR (126 MHz, DMSO-d_6) spectrum of **6a**.

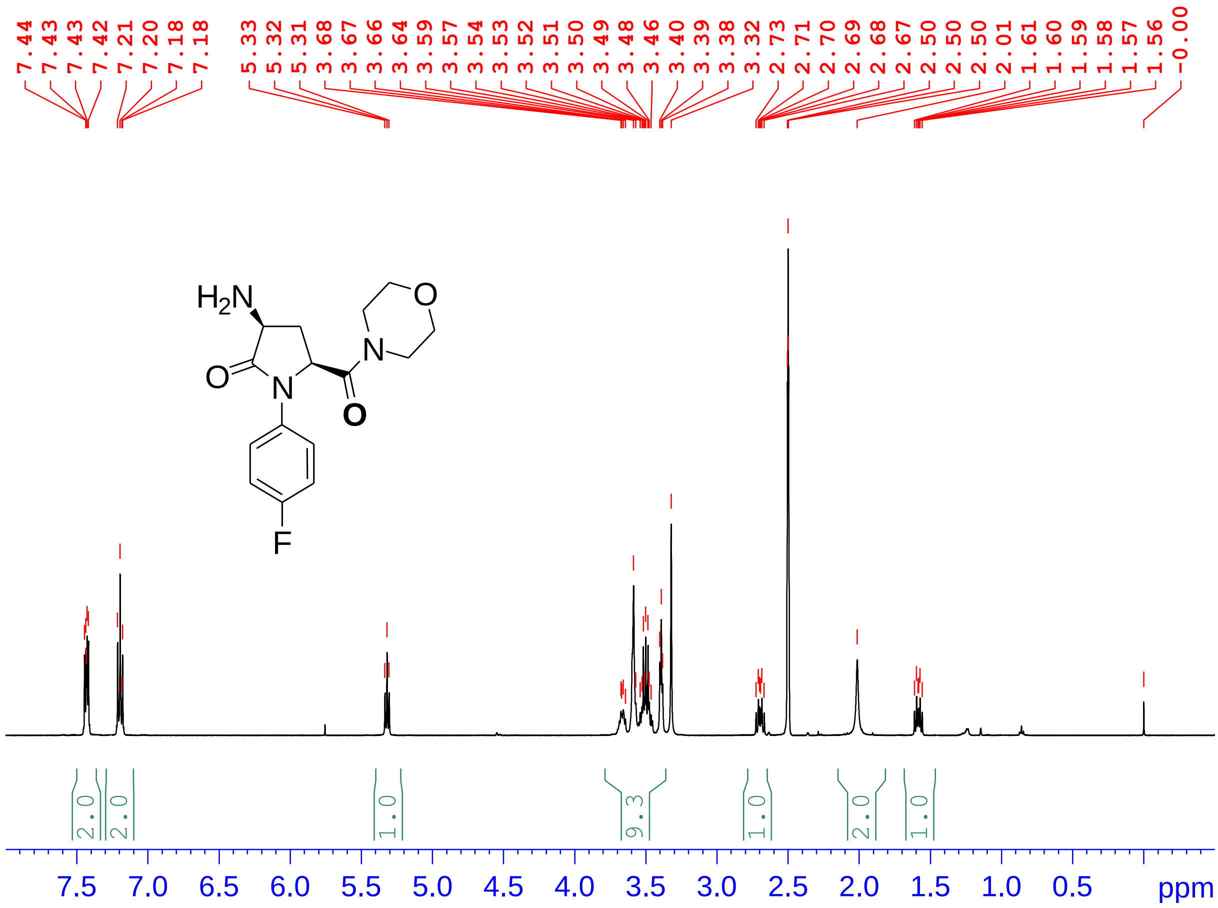

Figure S21. ^{1}H NMR (500 MHz, DMSO-d_6) spectrum of **6b**.

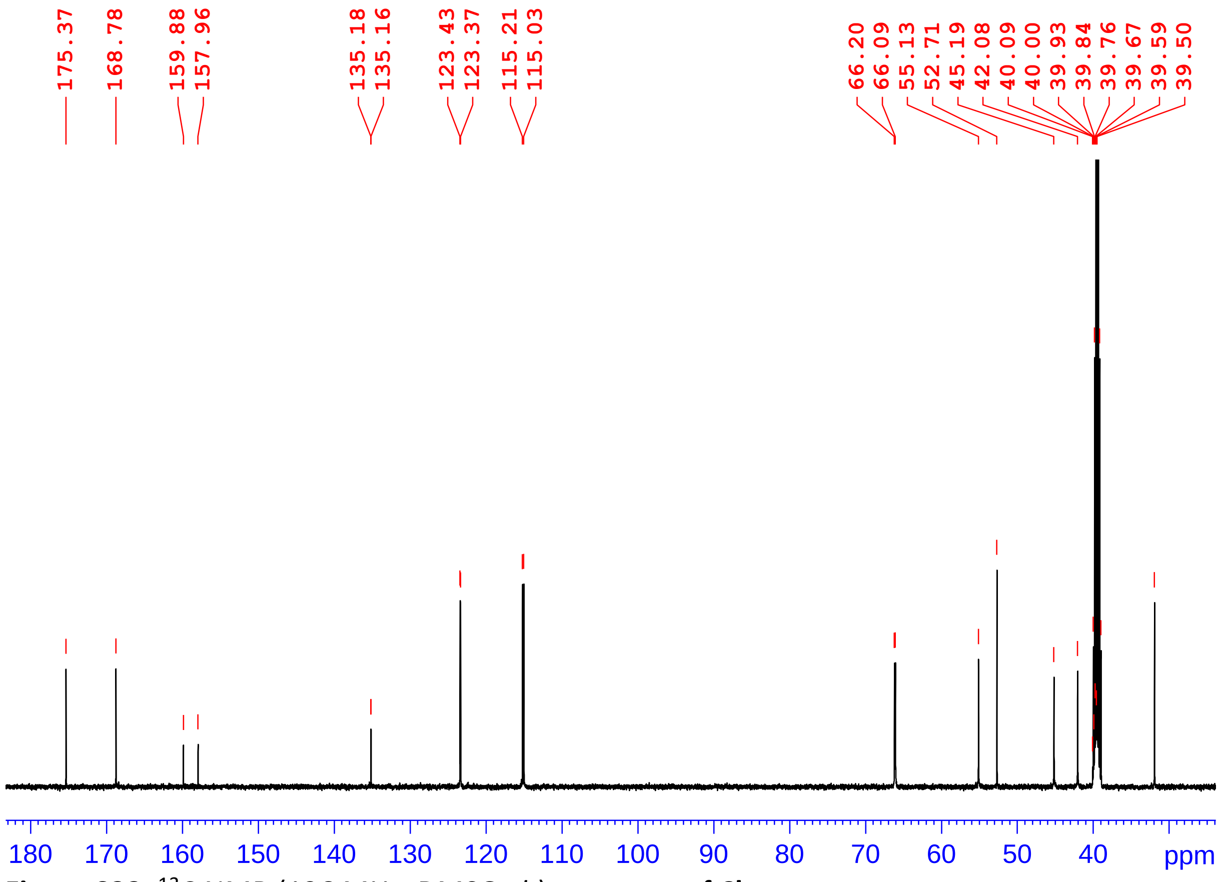

Figure S22. ^{13}C NMR (126 MHz, DMSO-d_6) spectrum of **6b**.

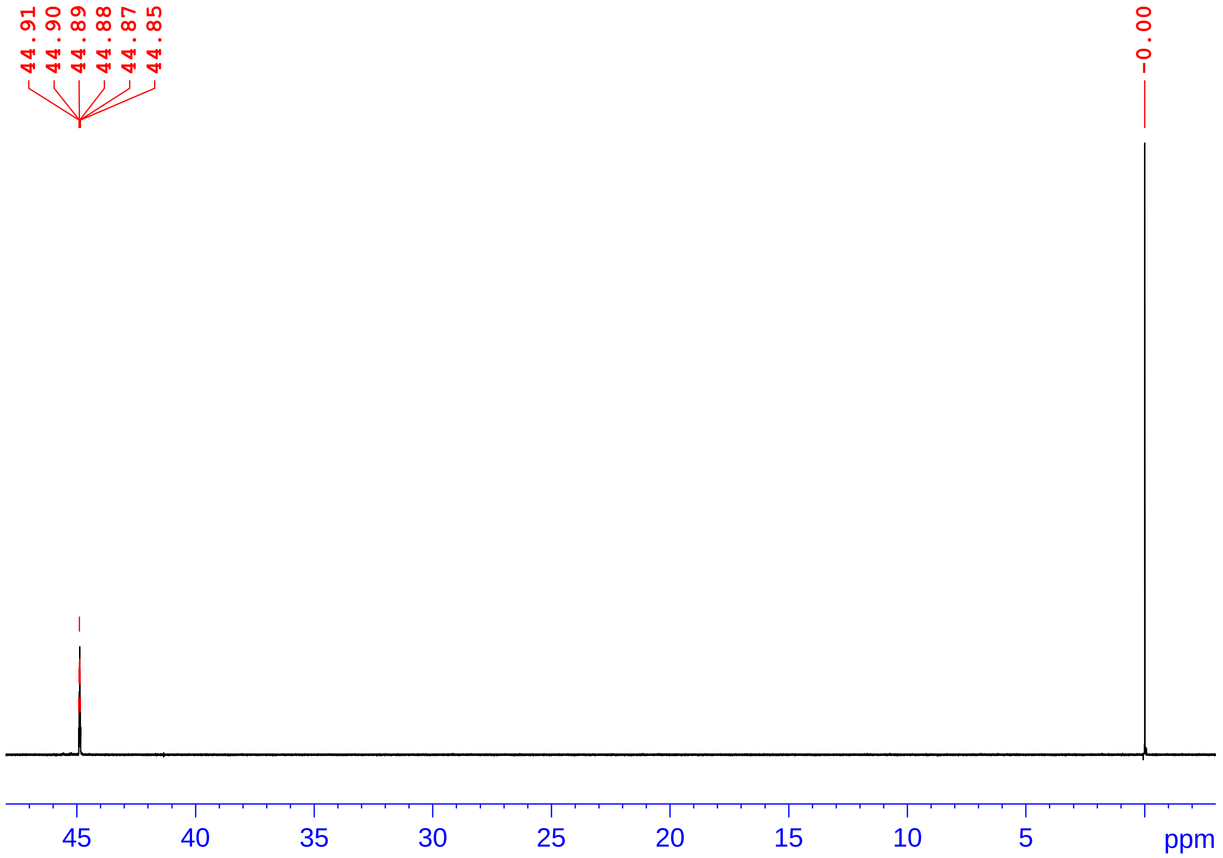

Figure S23. ^{19}F NMR (376 MHz, DMSO-d_6) spectrum of **6b**.

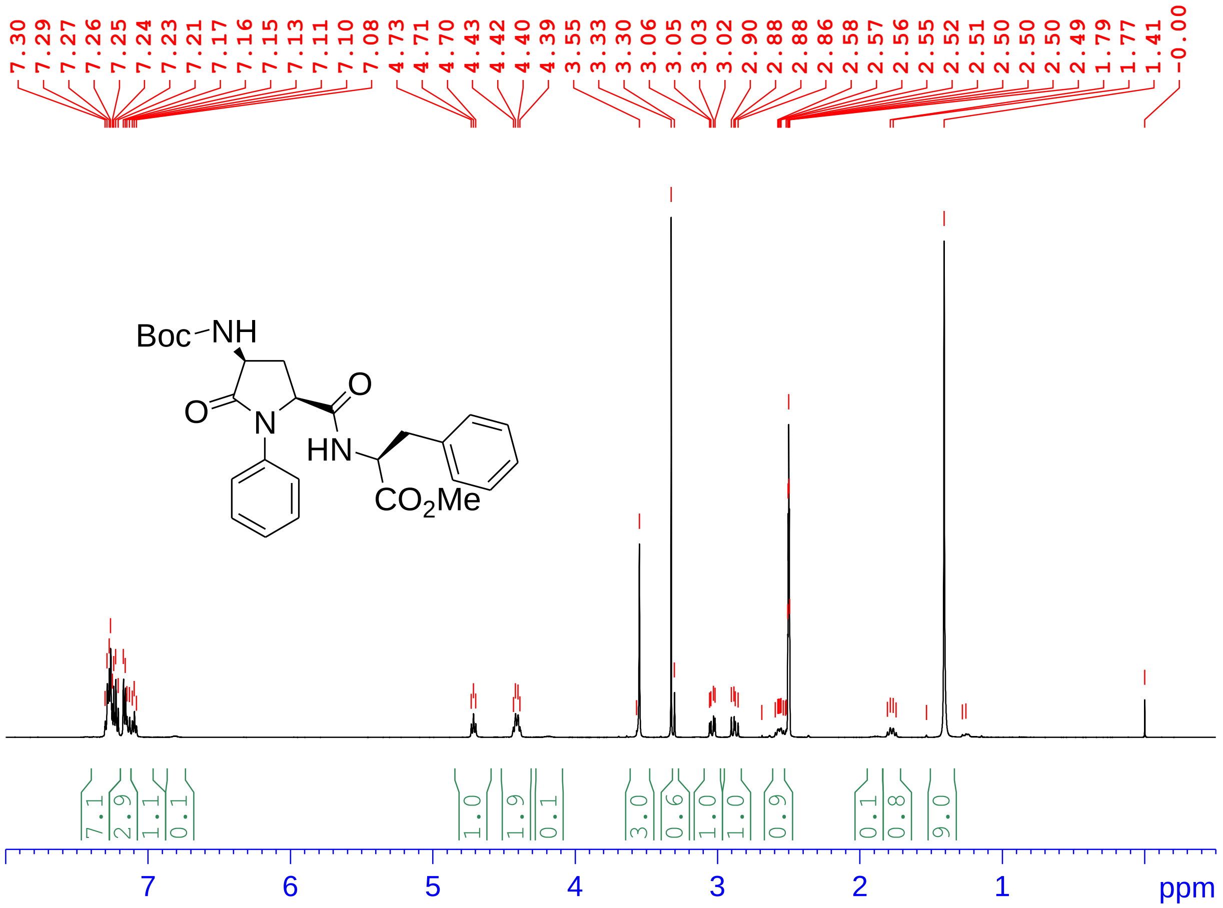

Figure S24. ^{1}H NMR (500 MHz, DMSO-d_6) spectrum of **7a**.

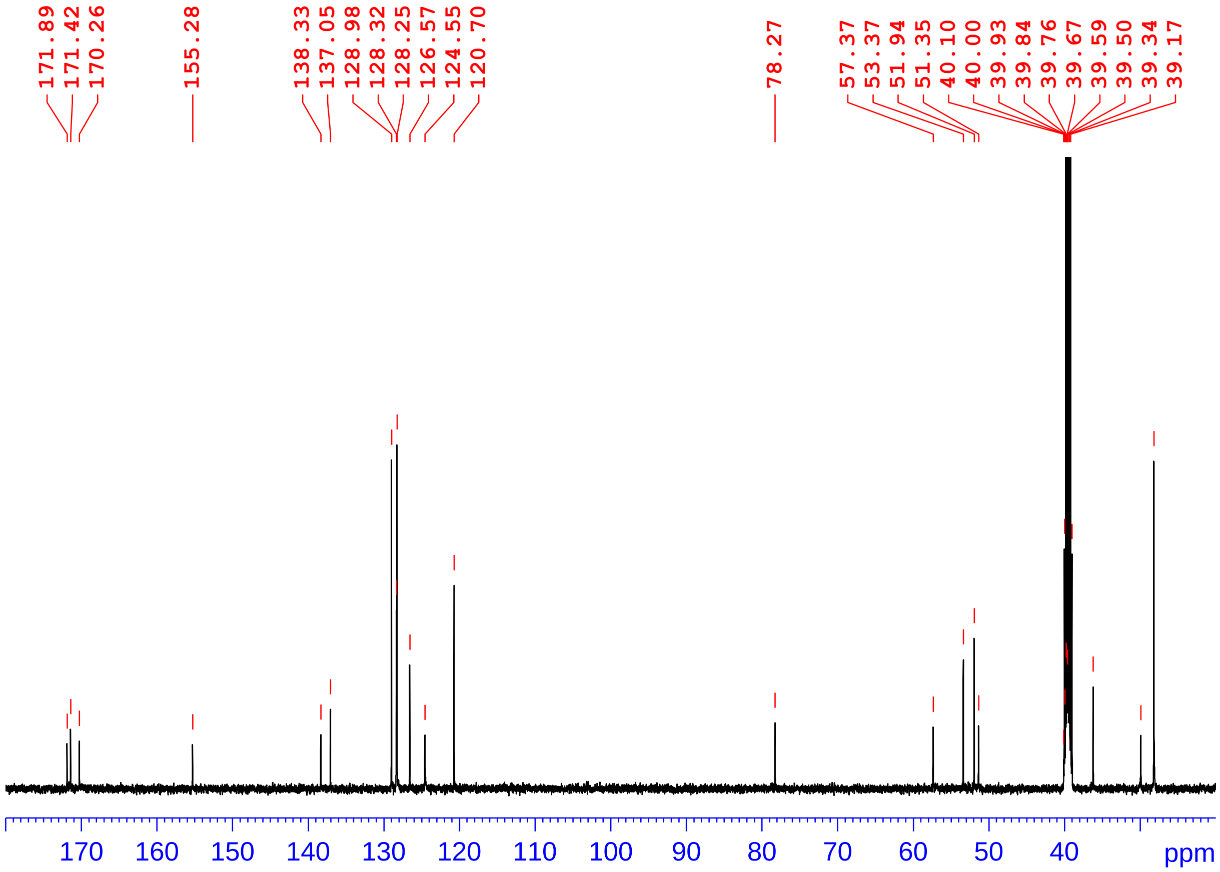

Figure S25. ^{13}C NMR (126 MHz, DMSO-d_6) spectrum of **7a**.

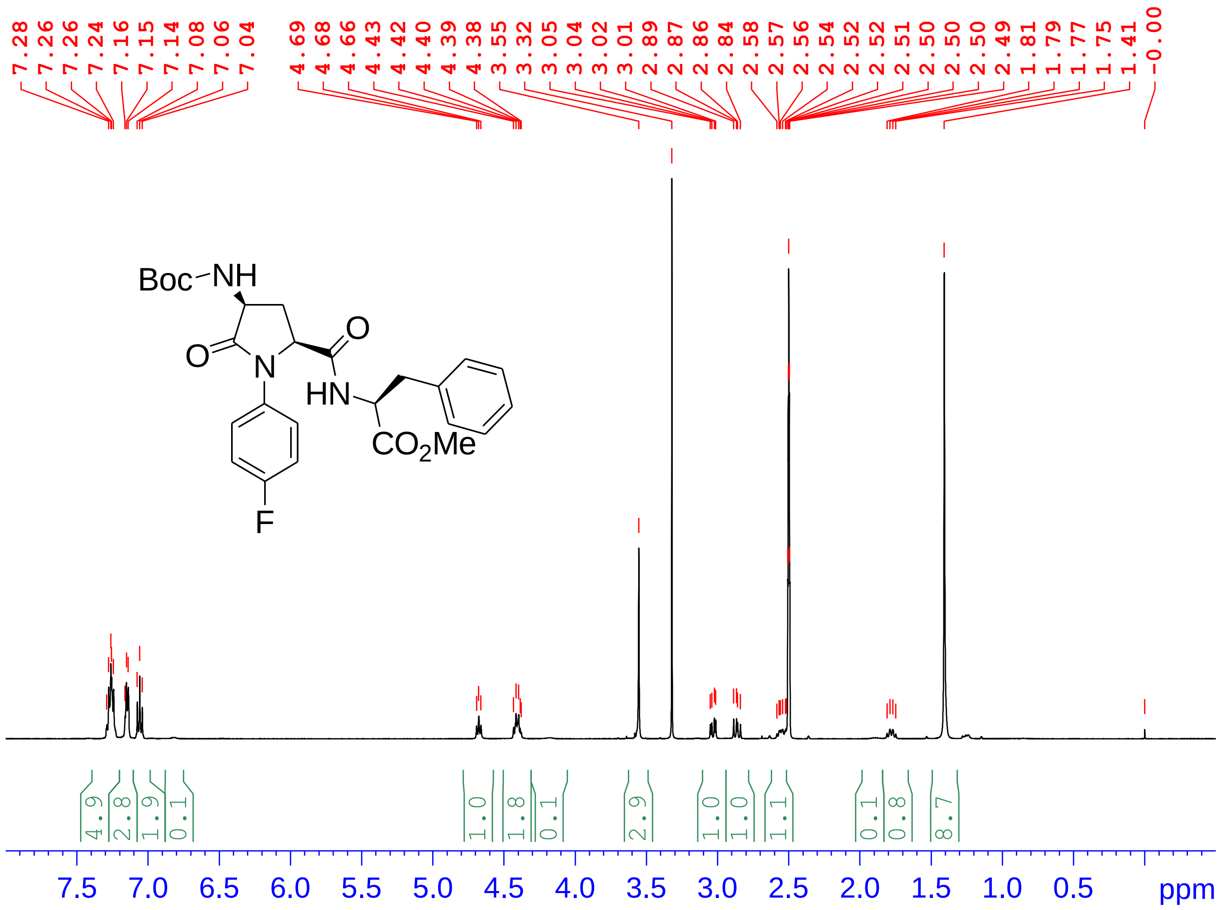

Figure S26. ^{1}H NMR (500 MHz, DMSO-d_6) spectrum of **7b**.

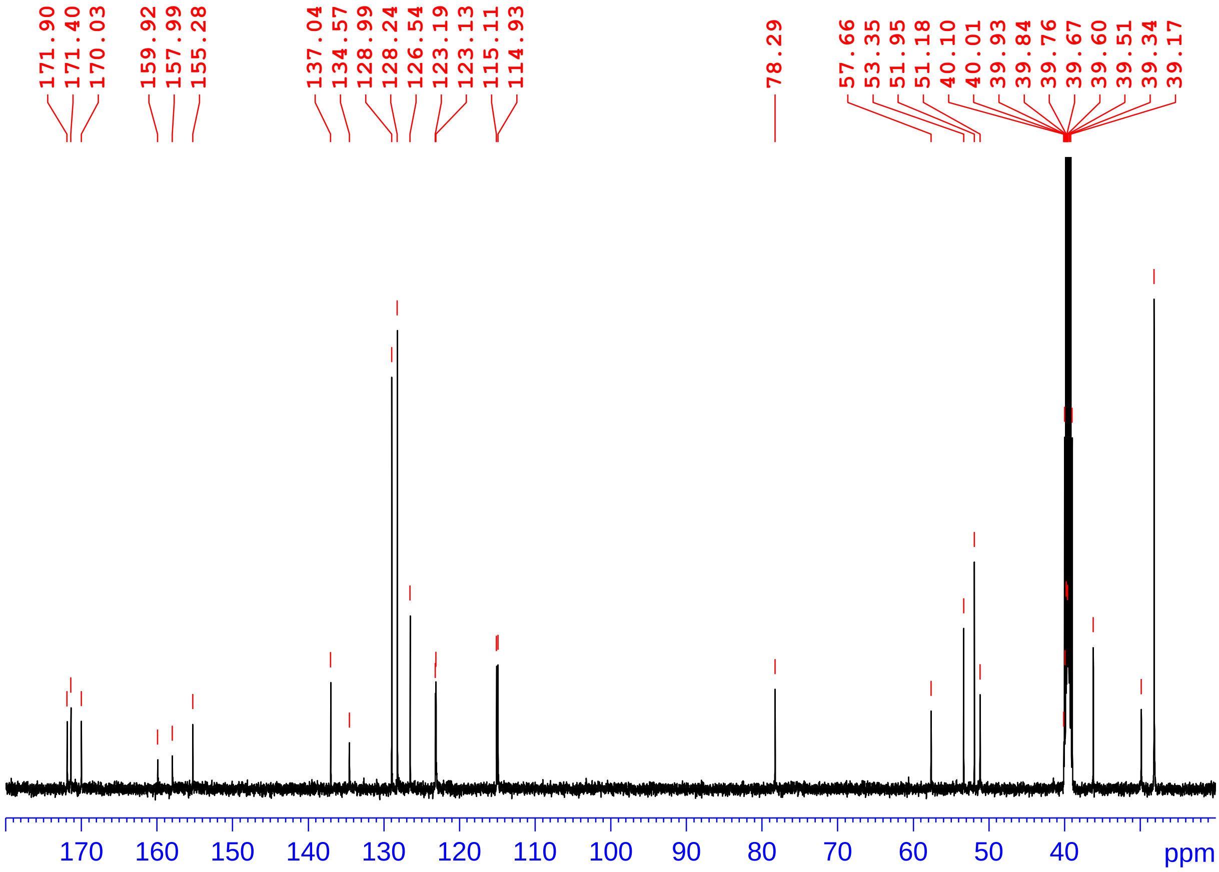

Figure S27. ^{13}C NMR (126 MHz, DMSO-d_6) spectrum of **7b**.

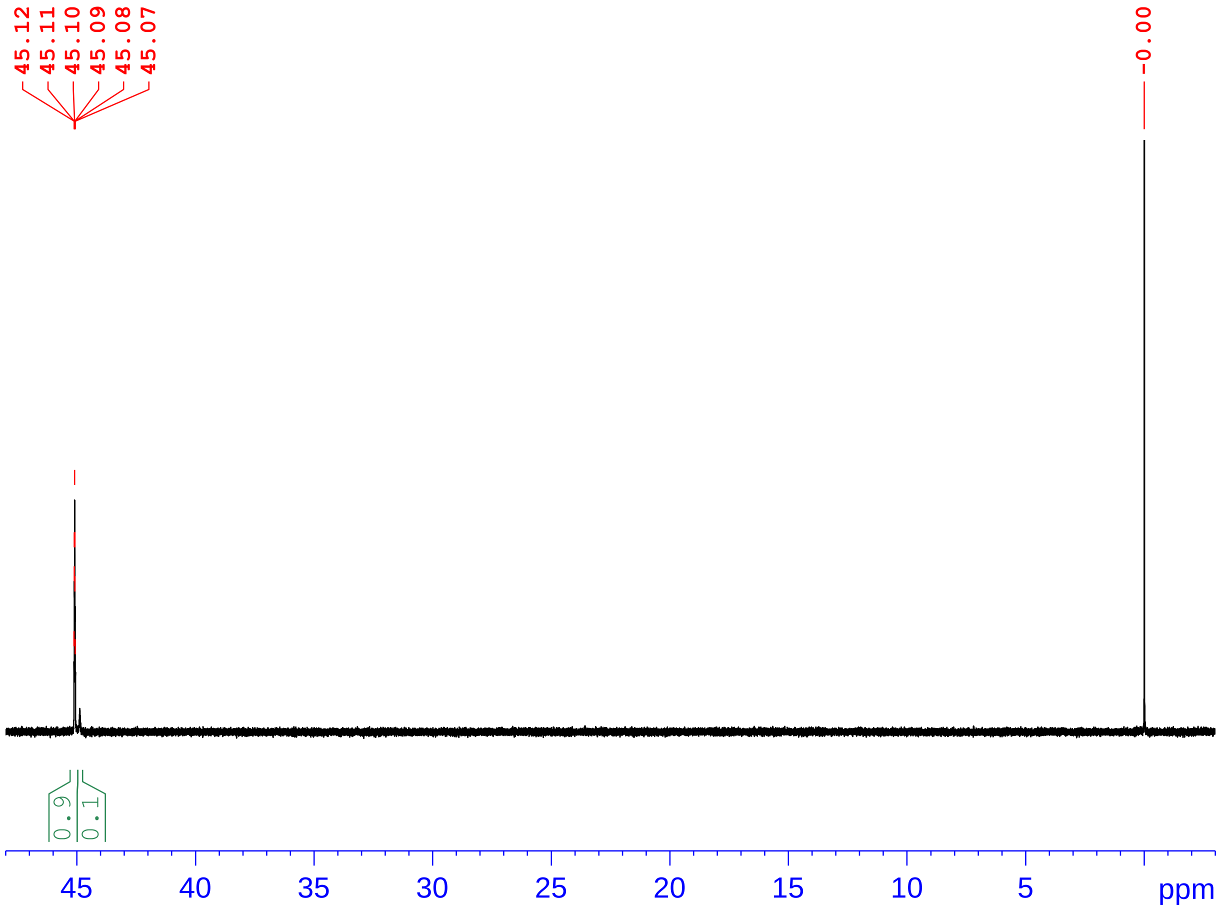

Figure S28. ^{19}F NMR (470 MHz, DMSO-d_6) spectrum of **7b**.

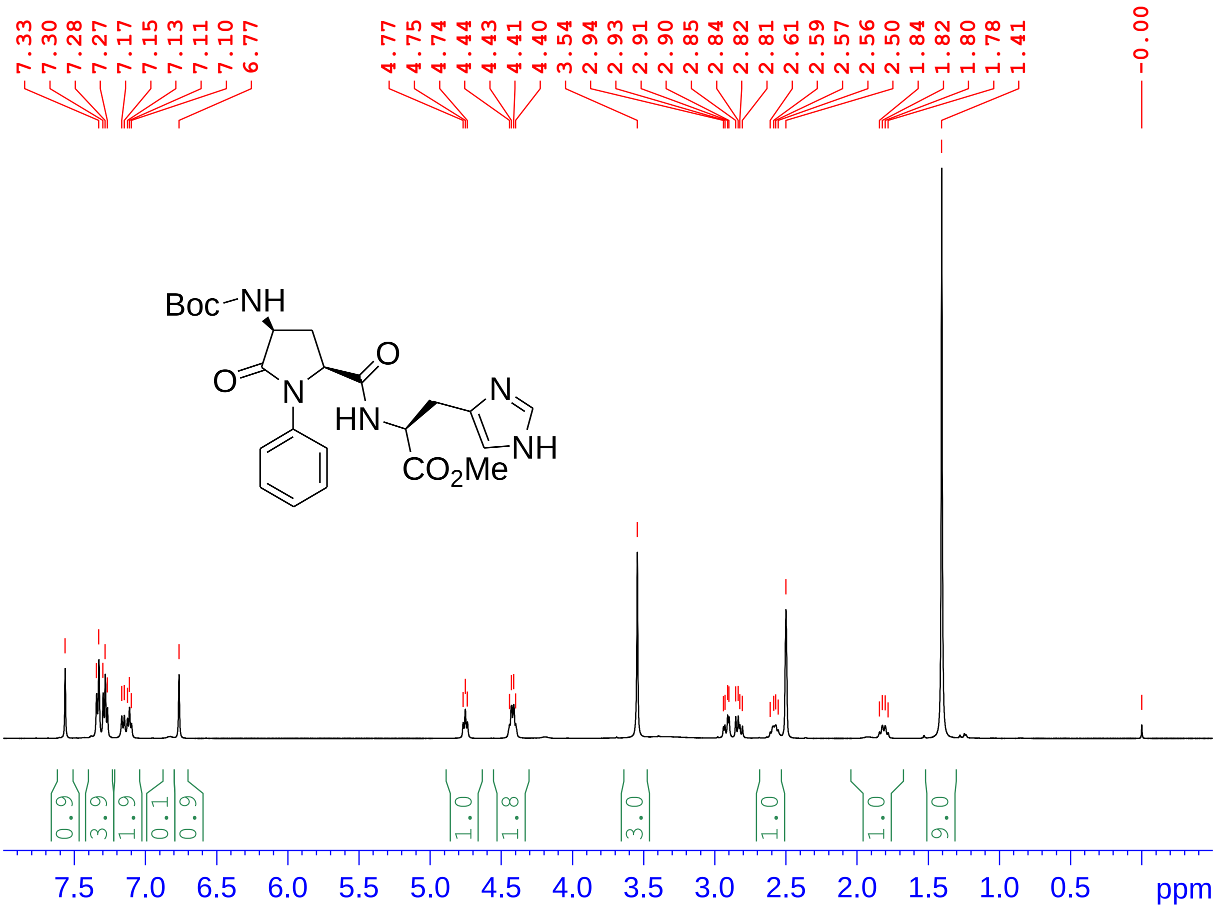

Figure S29. ^{1}H NMR (500 MHz, DMSO-d_6) spectrum of **8a**.

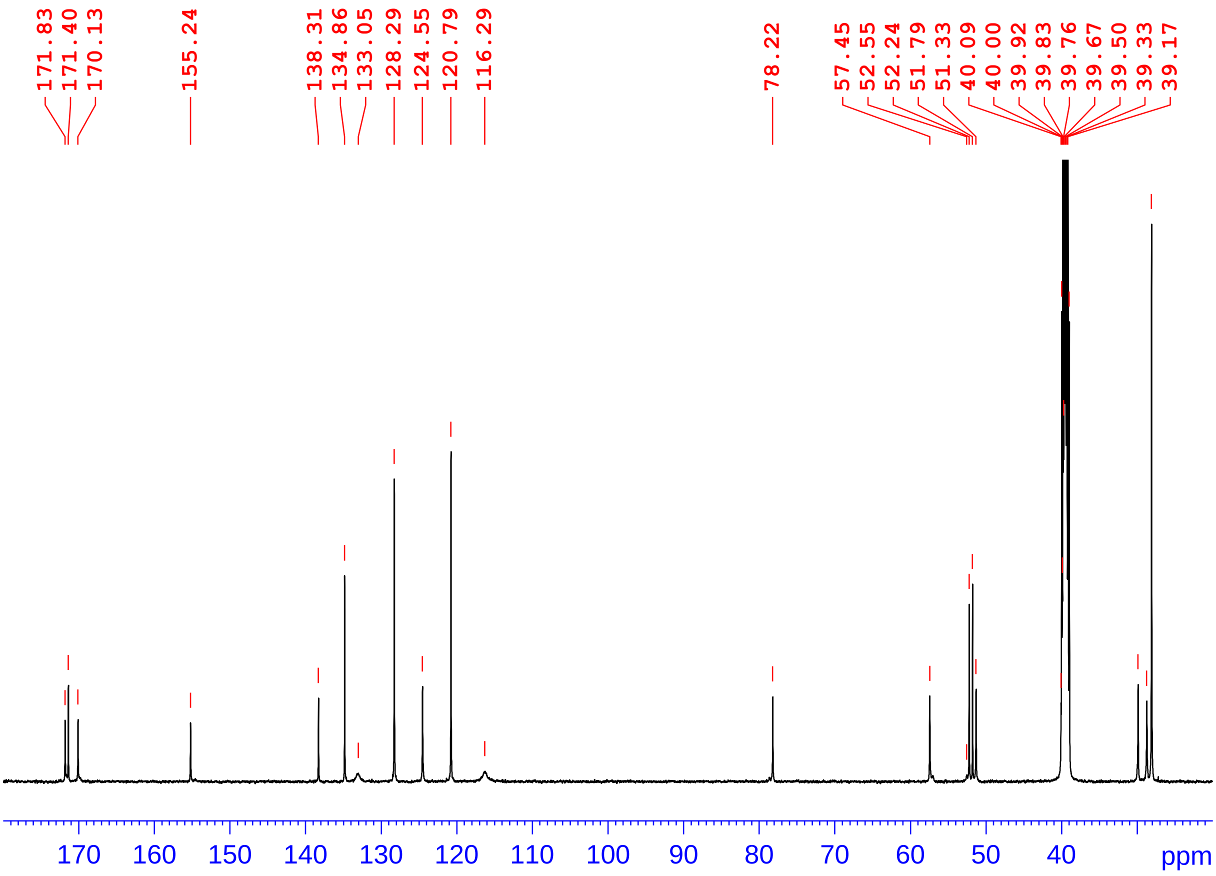

Figure S30. ^{13}C NMR (126 MHz, DMSO-d_6) spectrum of **8a**.

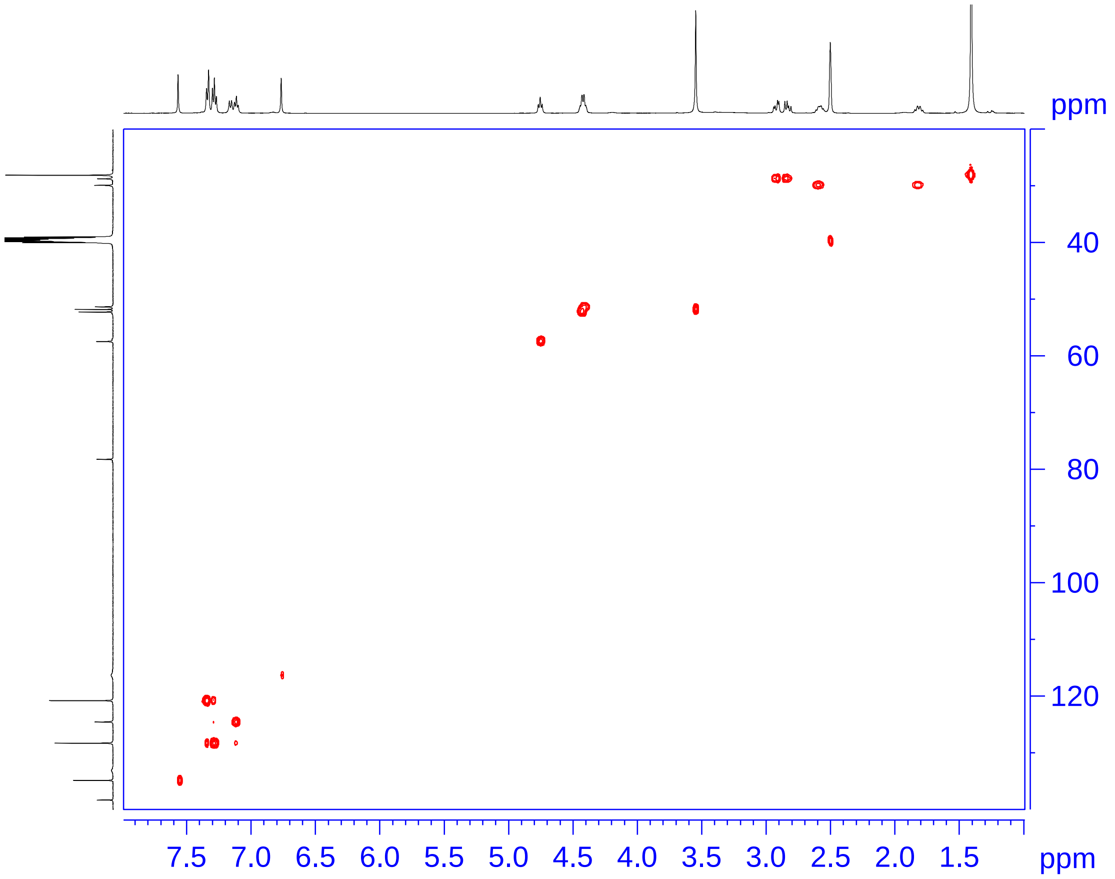

Figure S31. 2D ^{1}H-^{13}C HSQC (500 MHz, DMSO-d_6) spectrum of **8a**.

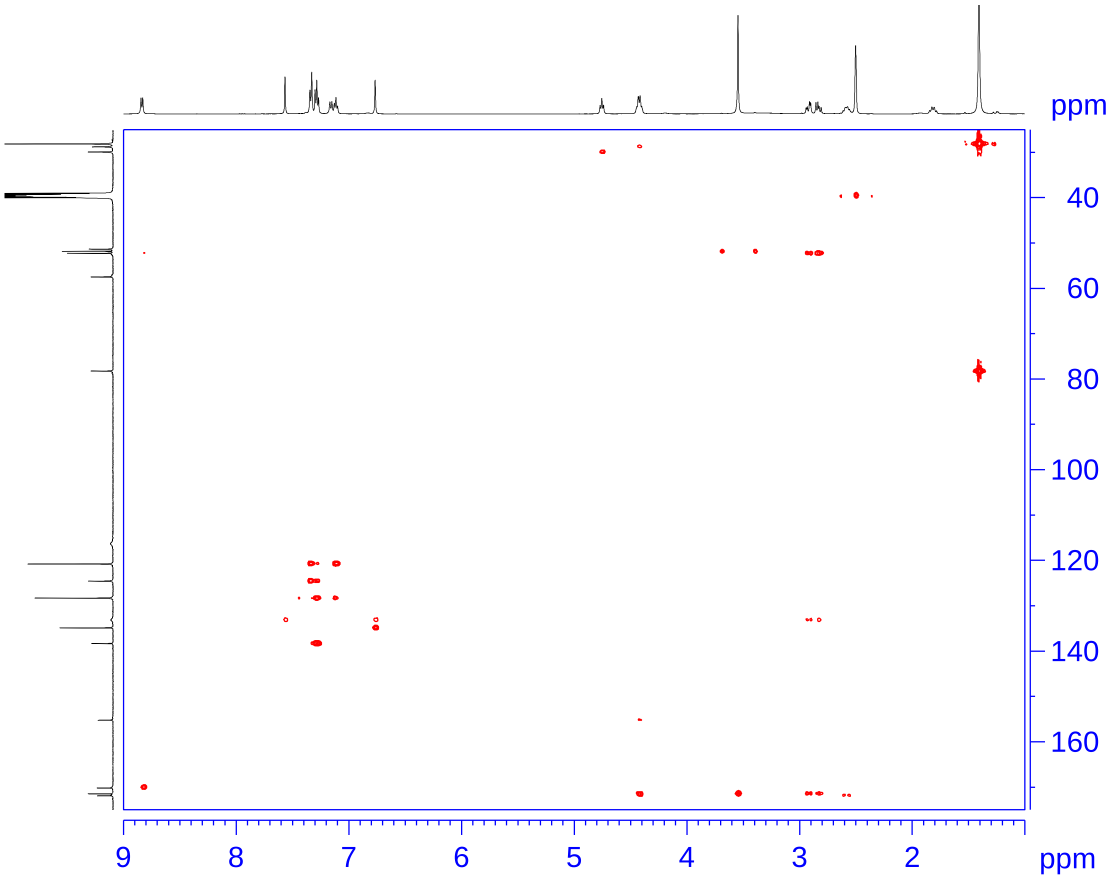

Figure S32. 2D ^{1}H-^{13}C HMBC (500 MHz, DMSO-d_6) spectrum of **8a**.

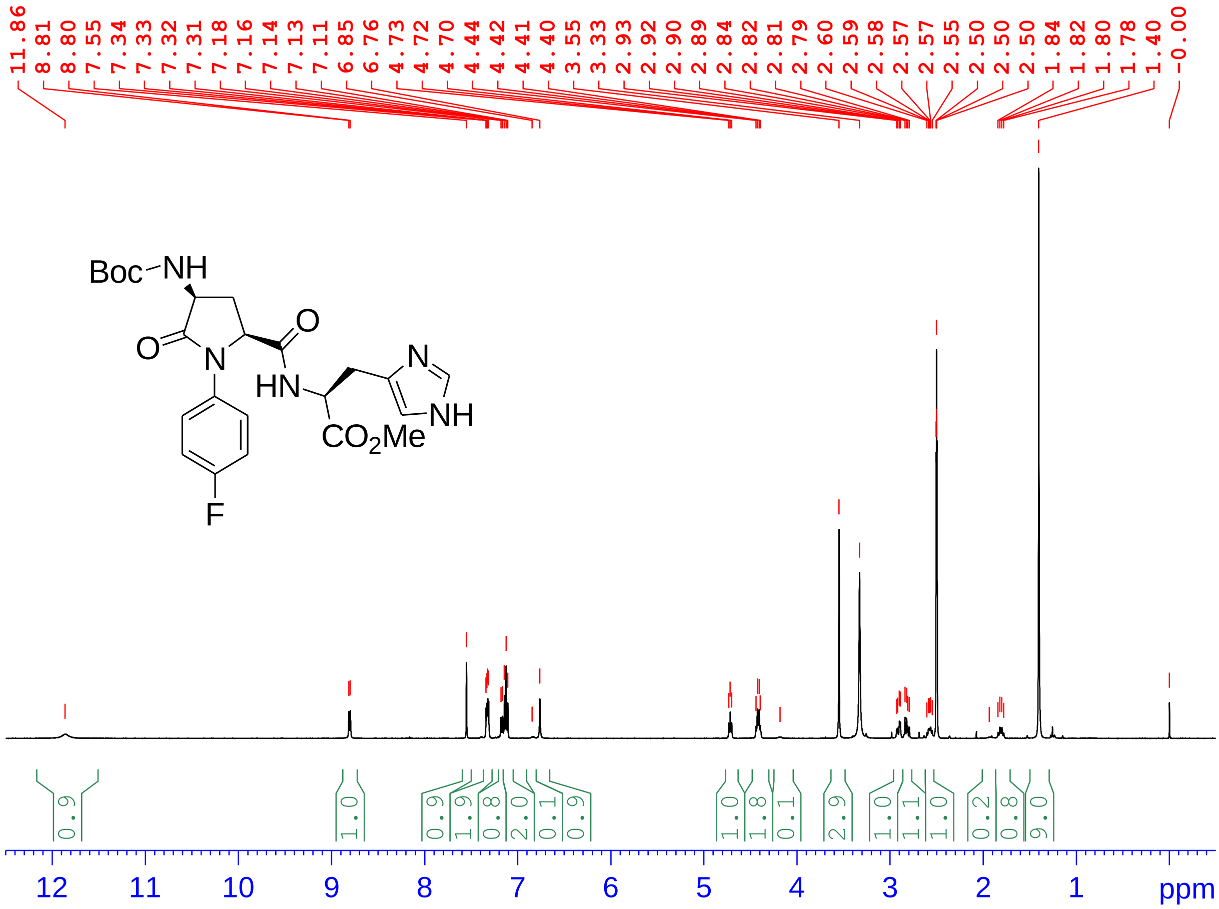

Figure S33. ^{1}H NMR (500 MHz, DMSO-d_6) spectrum of **8b**.

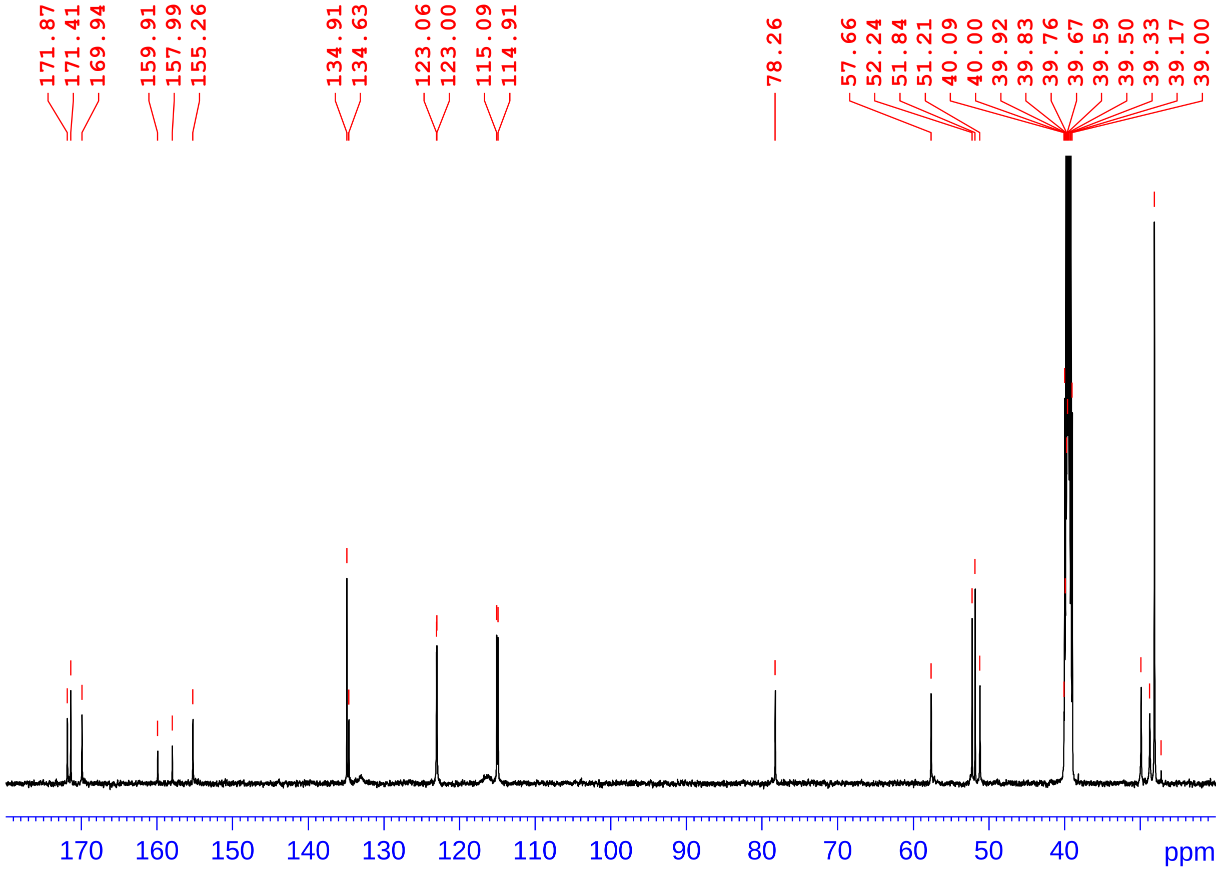

Figure S34. ^{13}C NMR (126 MHz, DMSO-d_6) spectrum of **8b**.

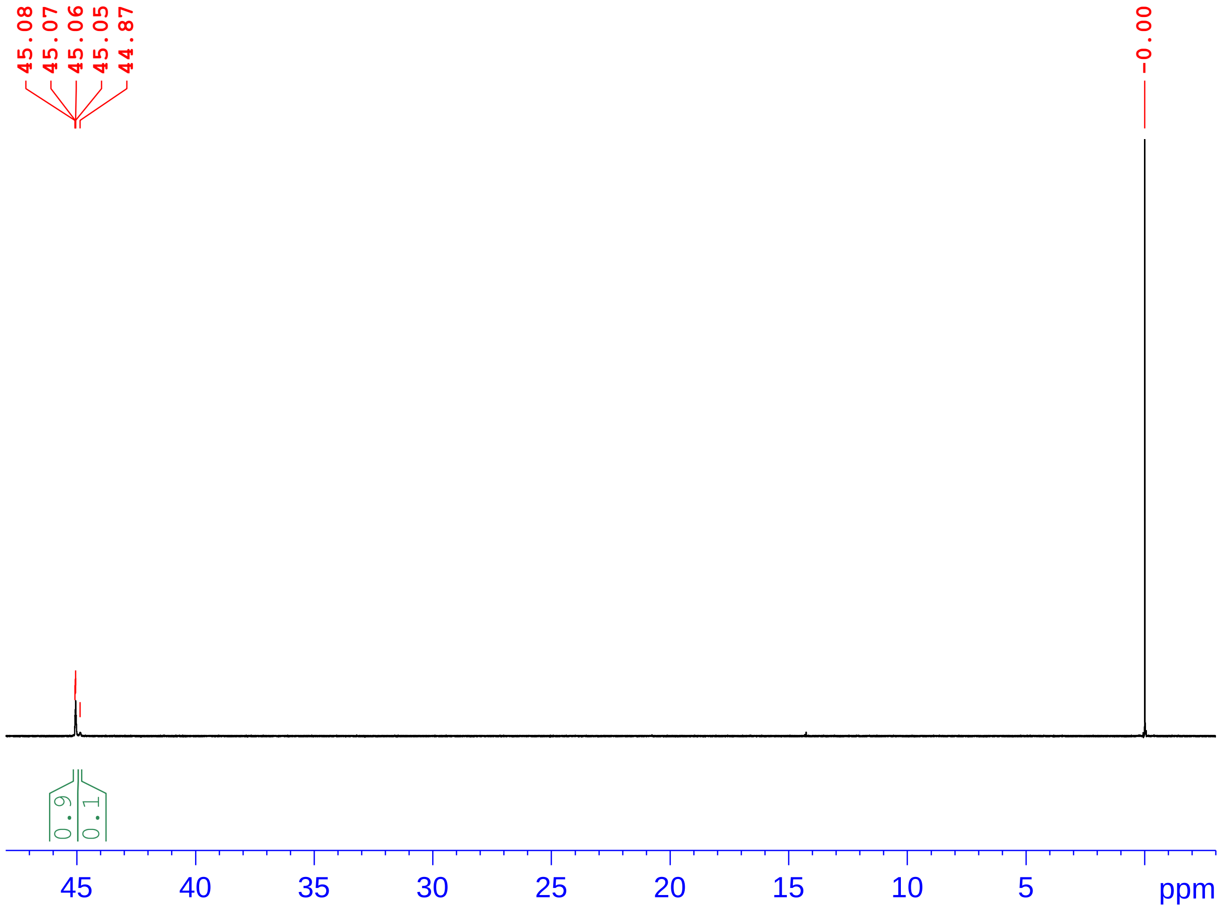

Figure S35. ^{19}F NMR (376 MHz, DMSO-d_6) spectrum of **8b**.

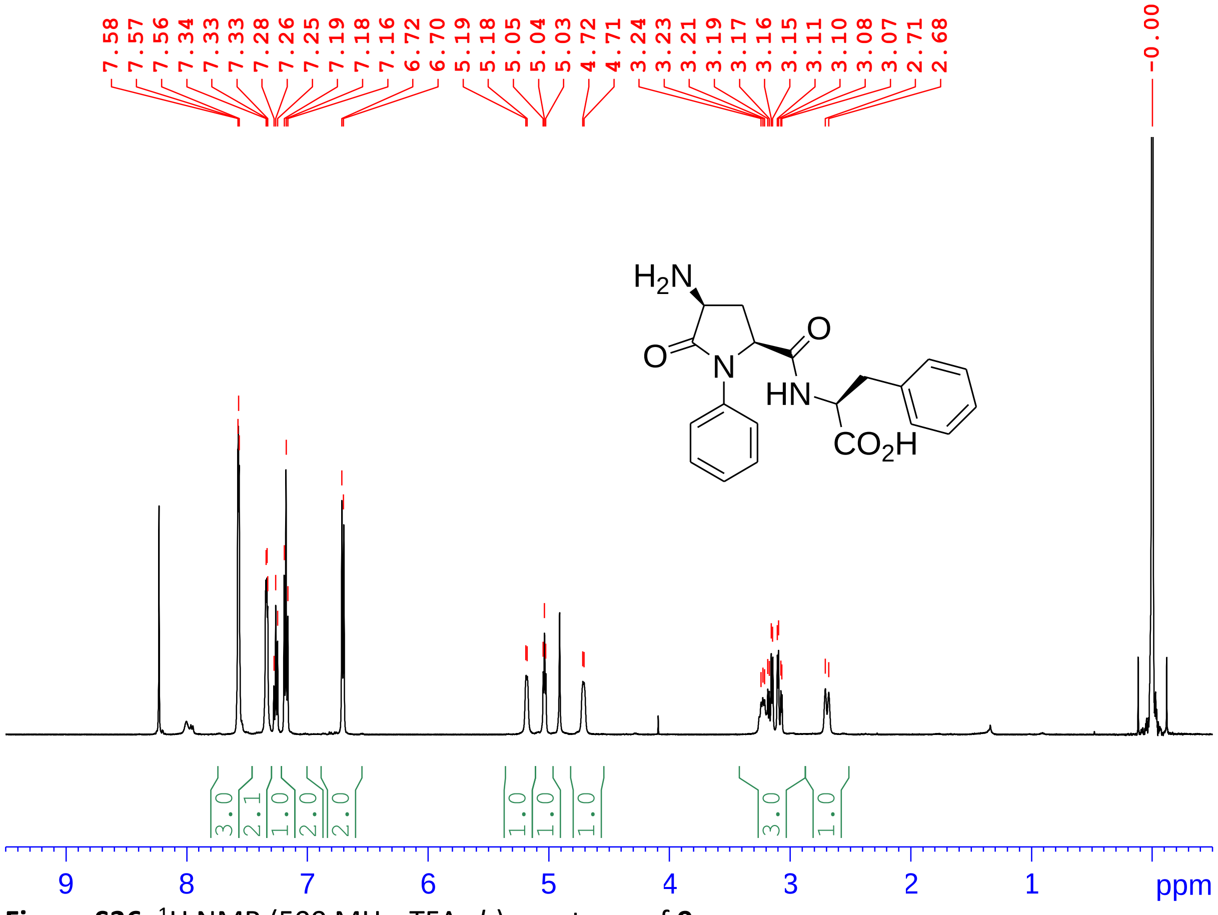

Figure S36. ^{1}H NMR (500 MHz, TFA-d_1) spectrum of **9a**.

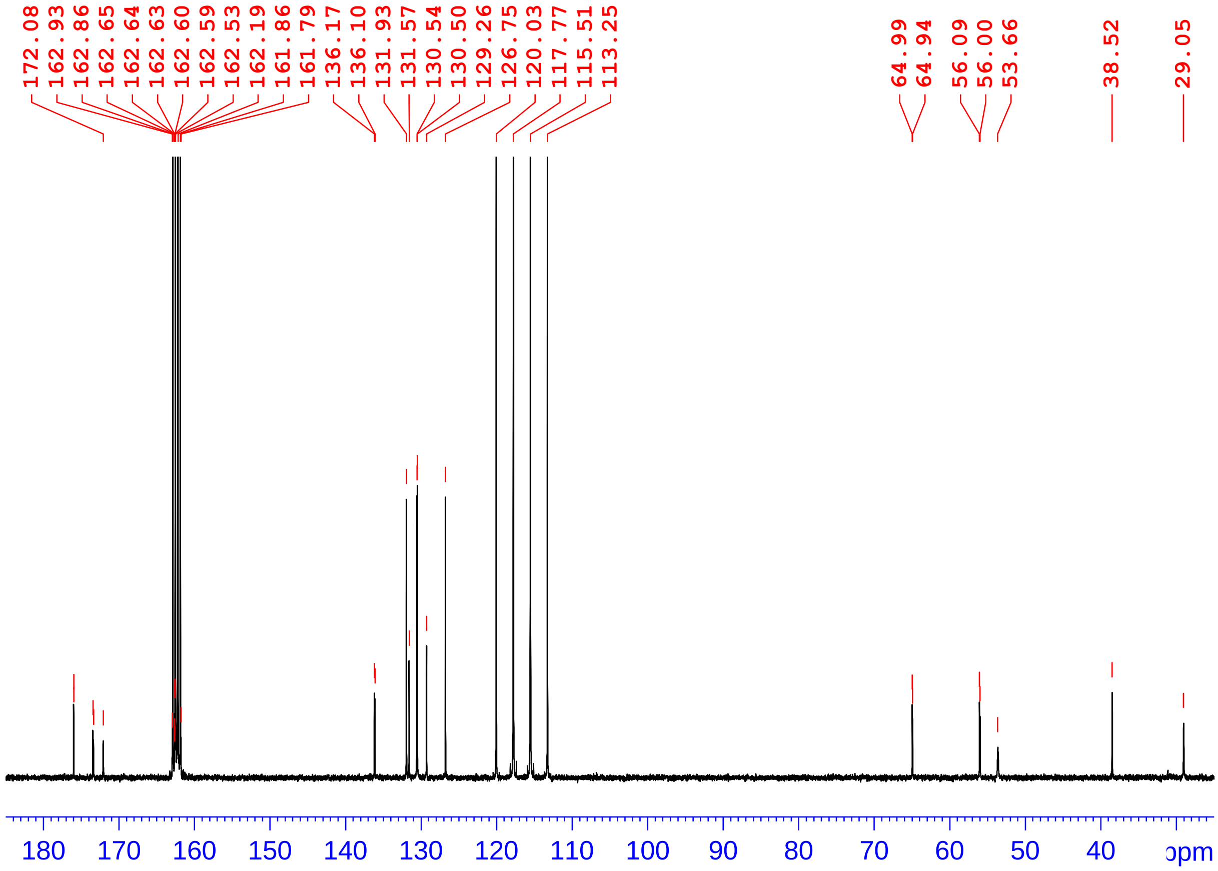

Figure S37. ^{13}C NMR (126 MHz, TFA-*d*$_1$) spectrum of **9a**.

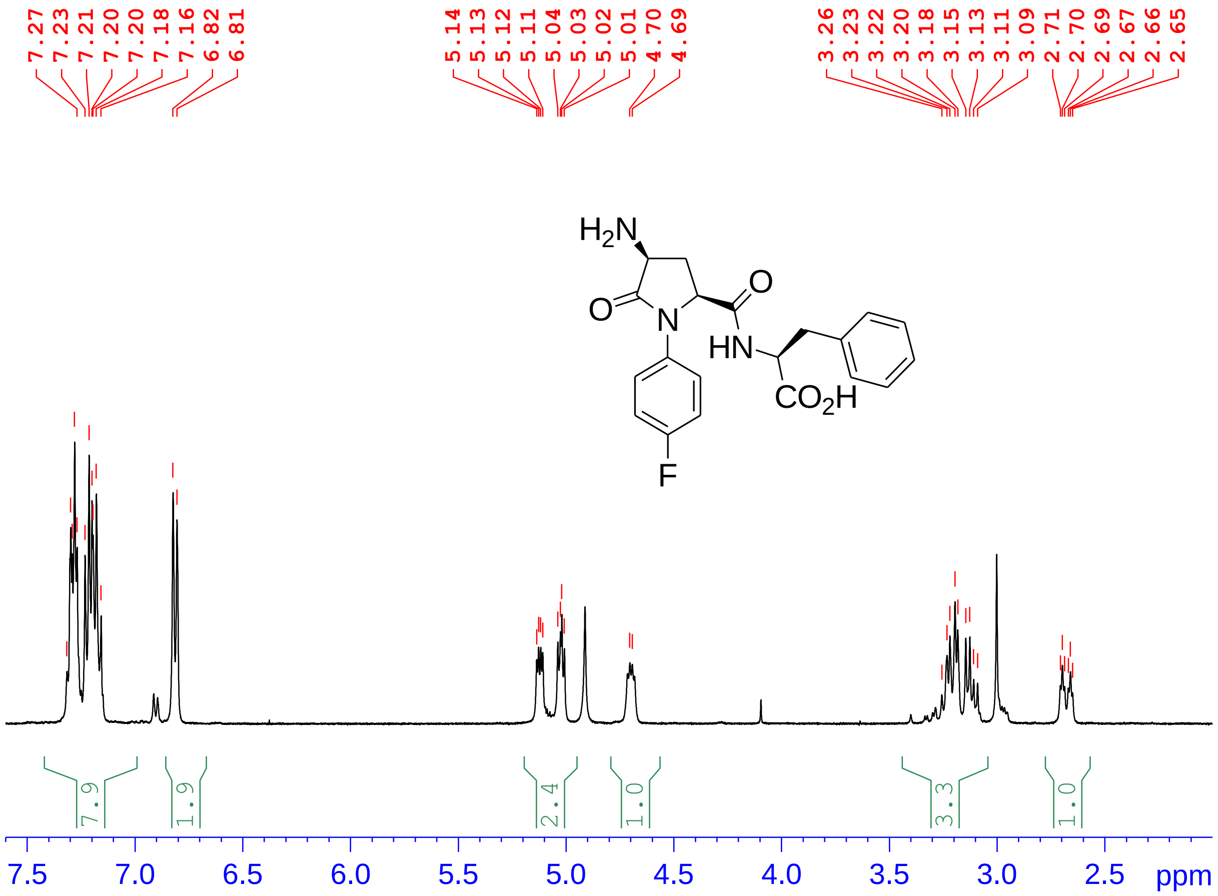

Figure S38. ^{1}H NMR (400 MHz, TFA-*d*$_1$) spectrum of **9b**.

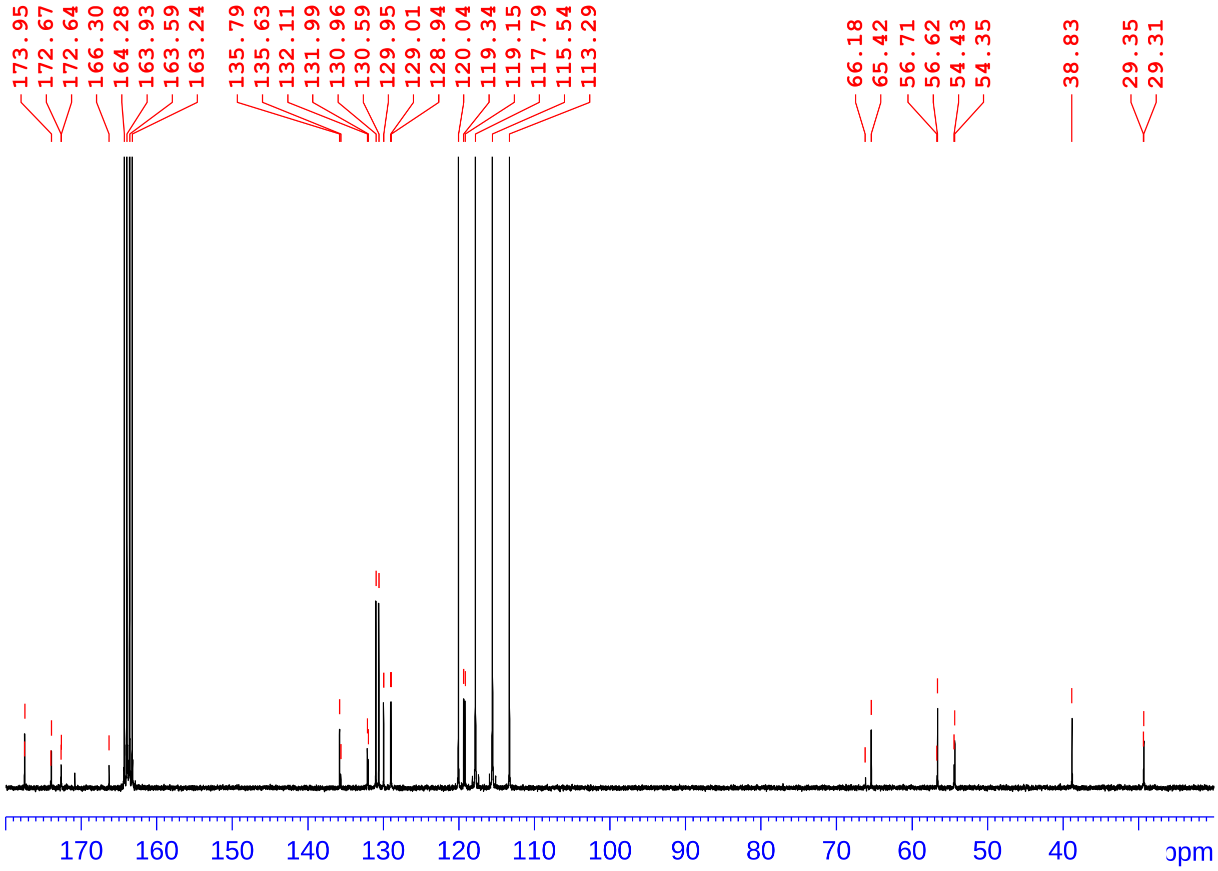

Figure S39. ^{13}C NMR (126 MHz, TFA-*d*$_1$) spectrum of **9b**.

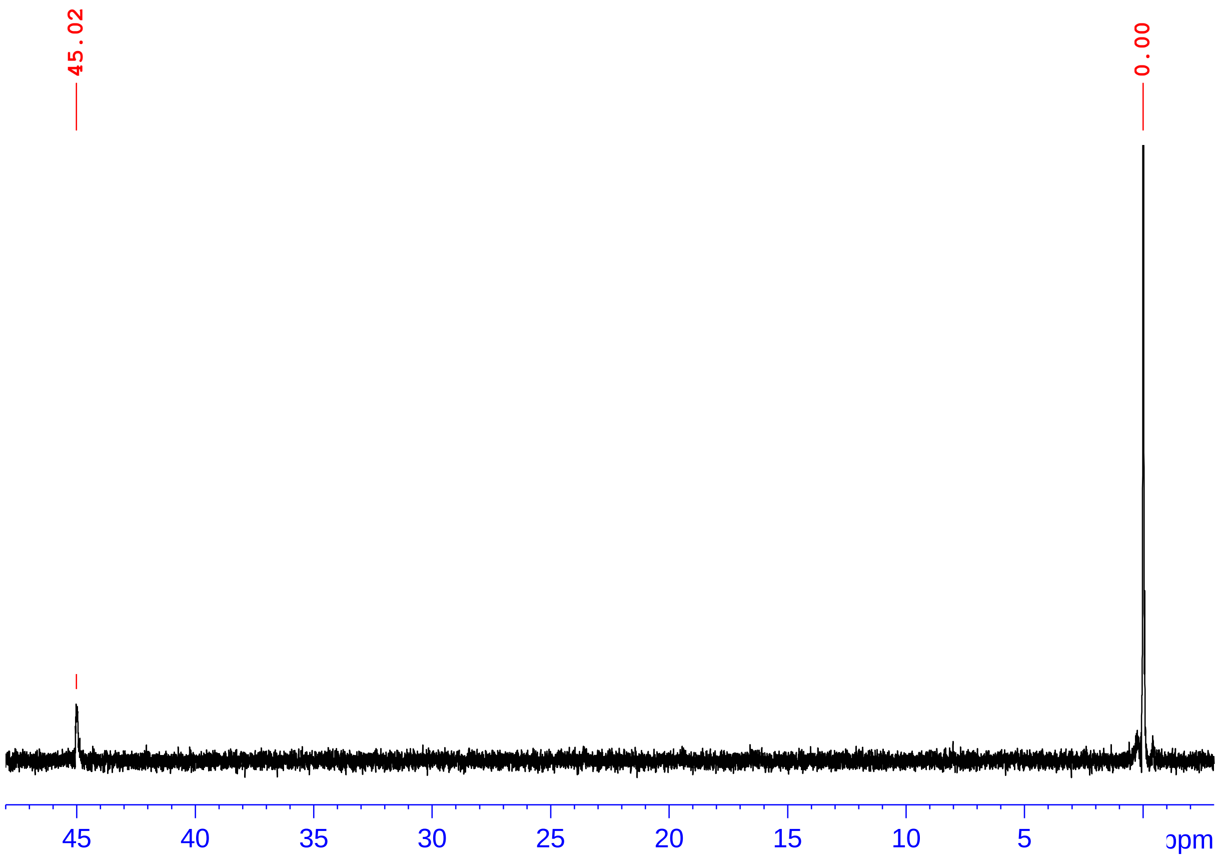

Figure S40. ^{19}F NMR (376 MHz, DMSO-*d*$_6$) spectrum of **9b**.

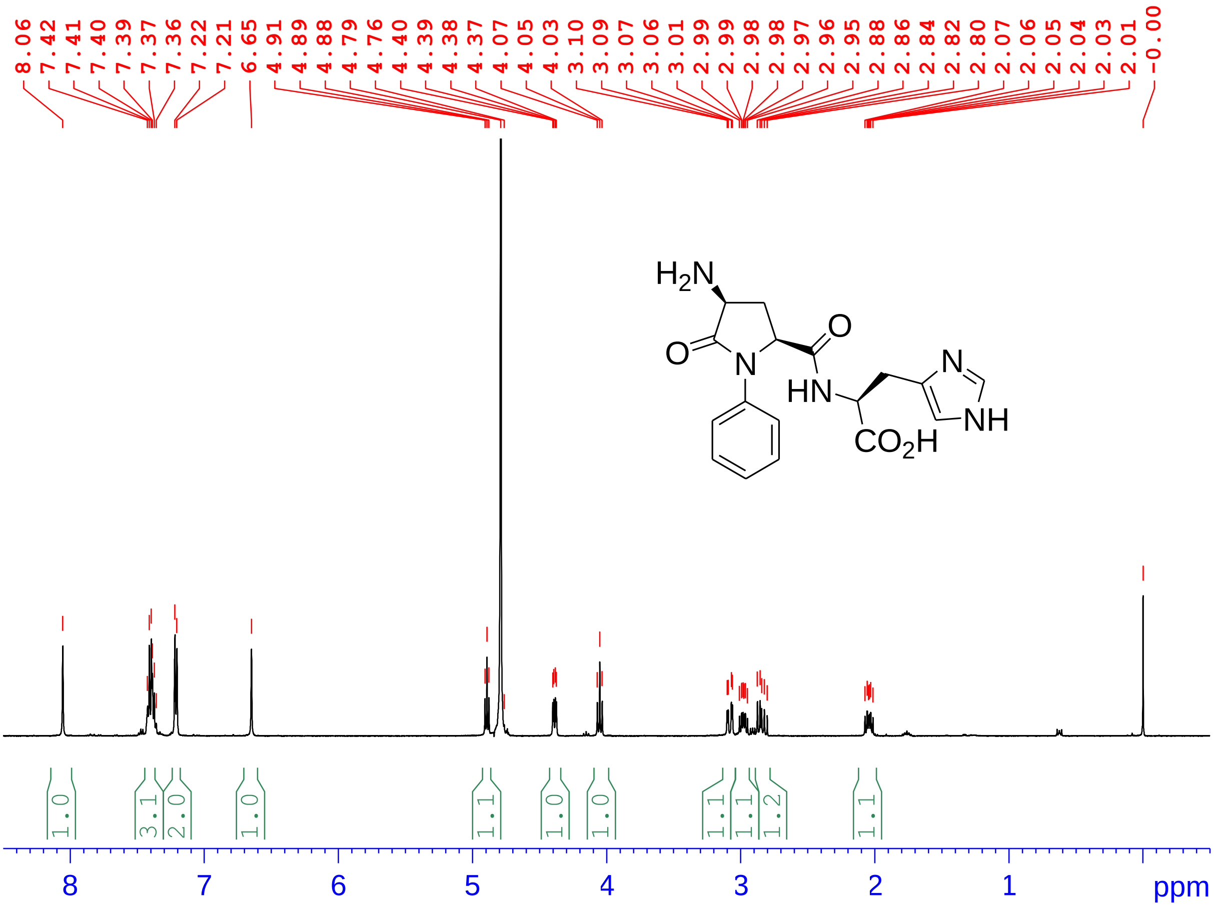

Figure S41. ^{1}H NMR (500 MHz, D_2O) spectrum of **10a**.

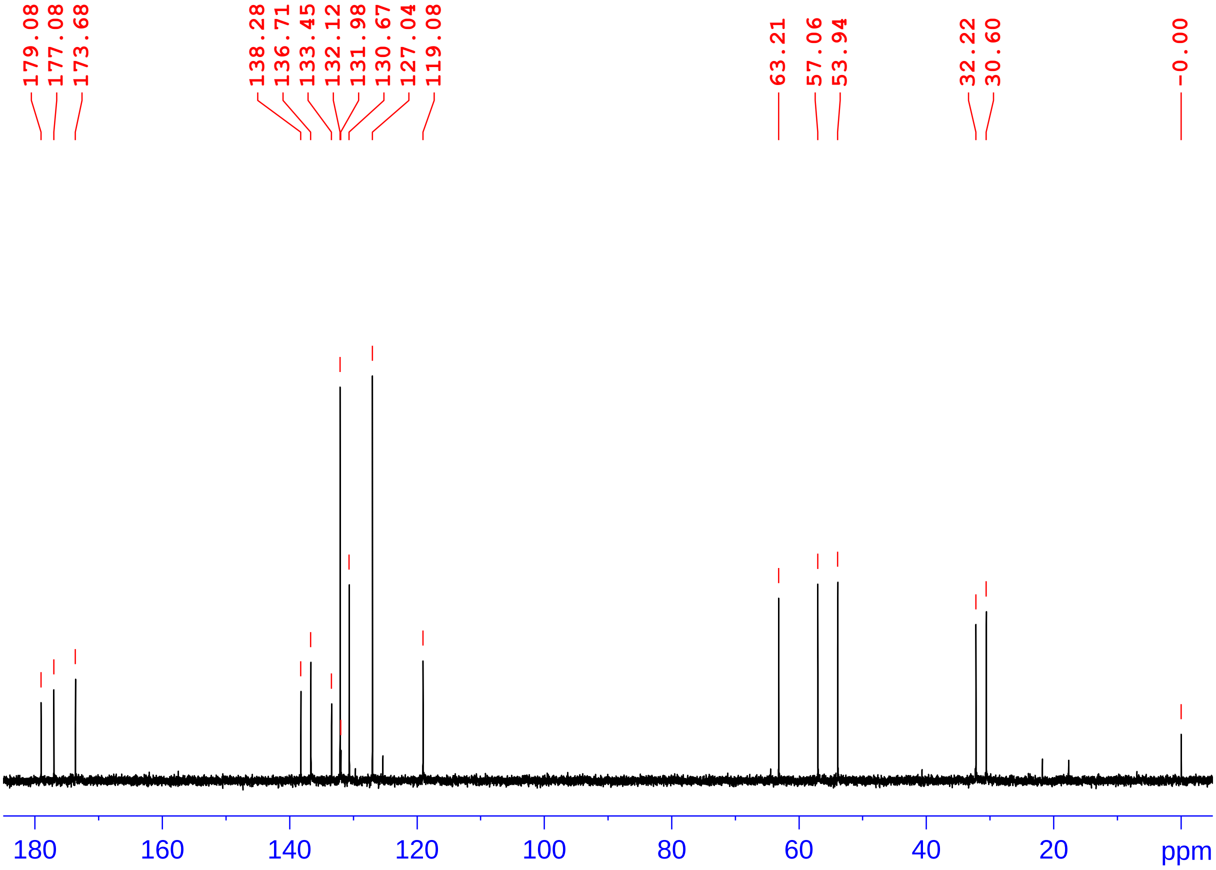

Figure S42. ^{13}C NMR (126 MHz, D_2O) spectrum of **10a**.

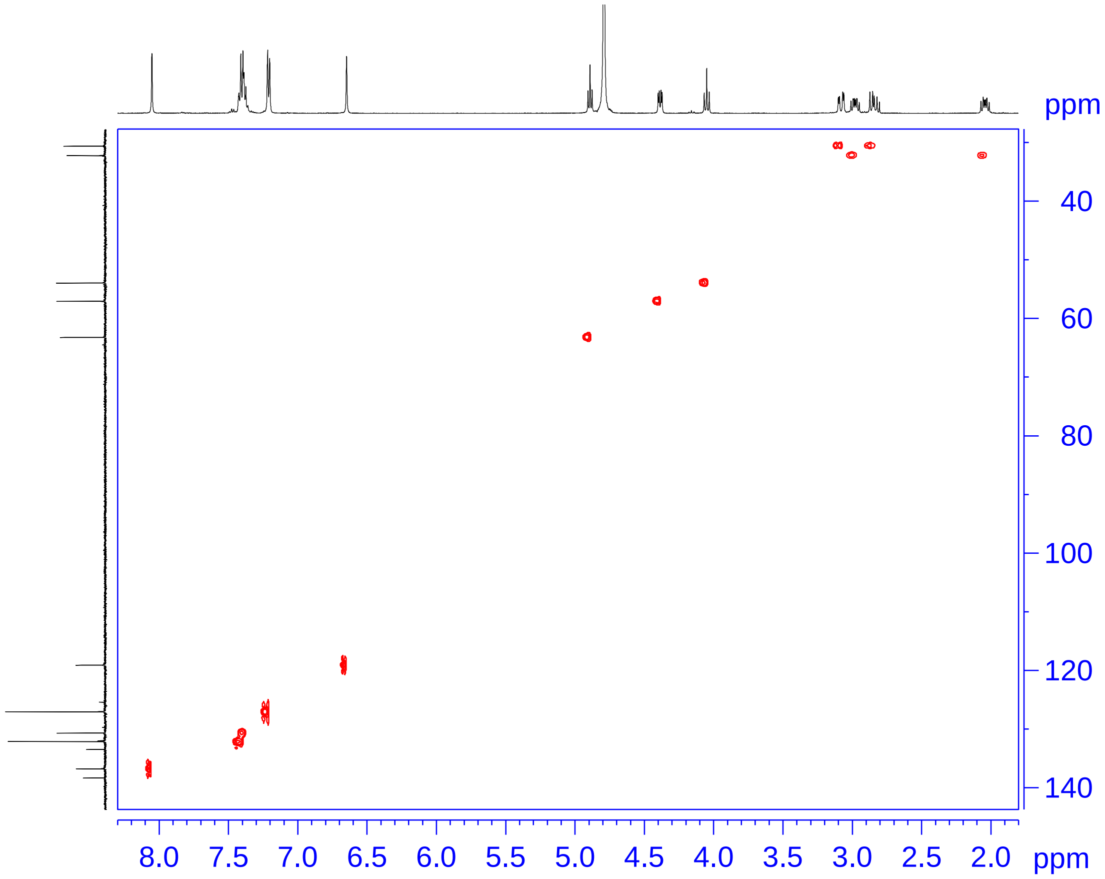

Figure S43. 2D ^1H-^{13}C HSQC (500 MHz, D_2O) spectrum of **10a**.

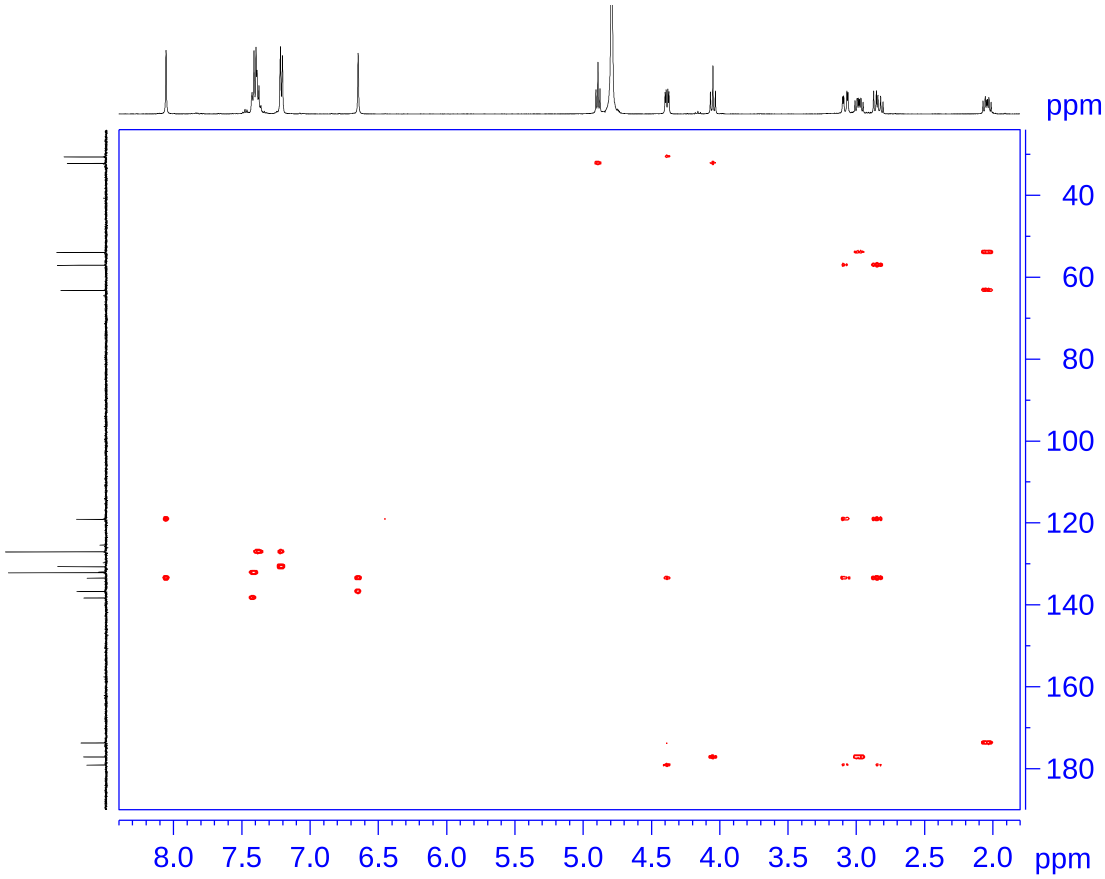

Figure S44. 2D ^1H-^{13}C HMBC (500 MHz, D_2O) spectrum of **10a**.

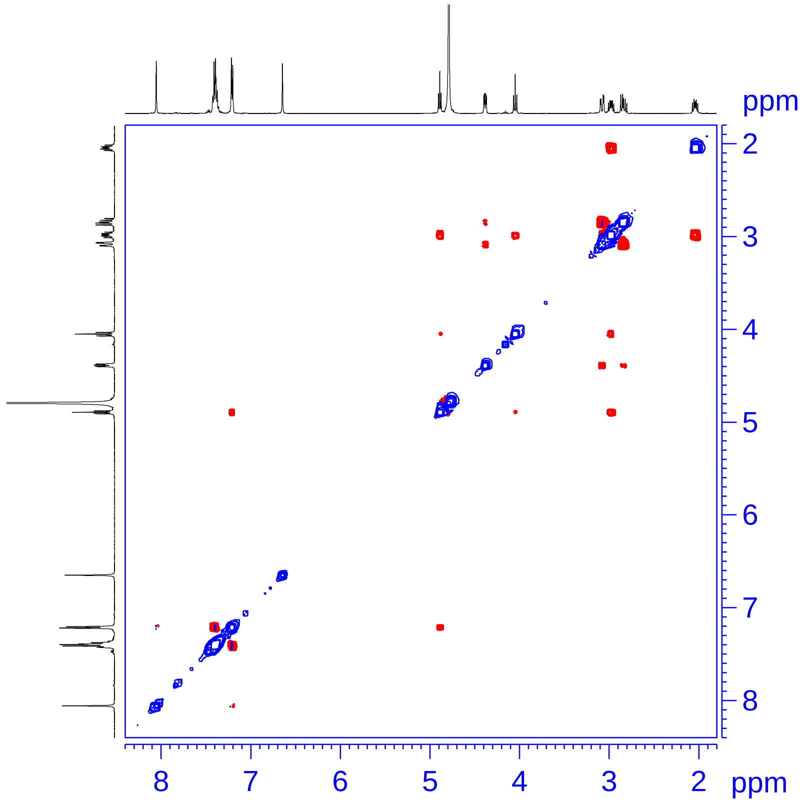

Figure S45. 2D ^{1}H-^{1}H NOESY (500 MHz, D_2O) spectrum of **10a**.

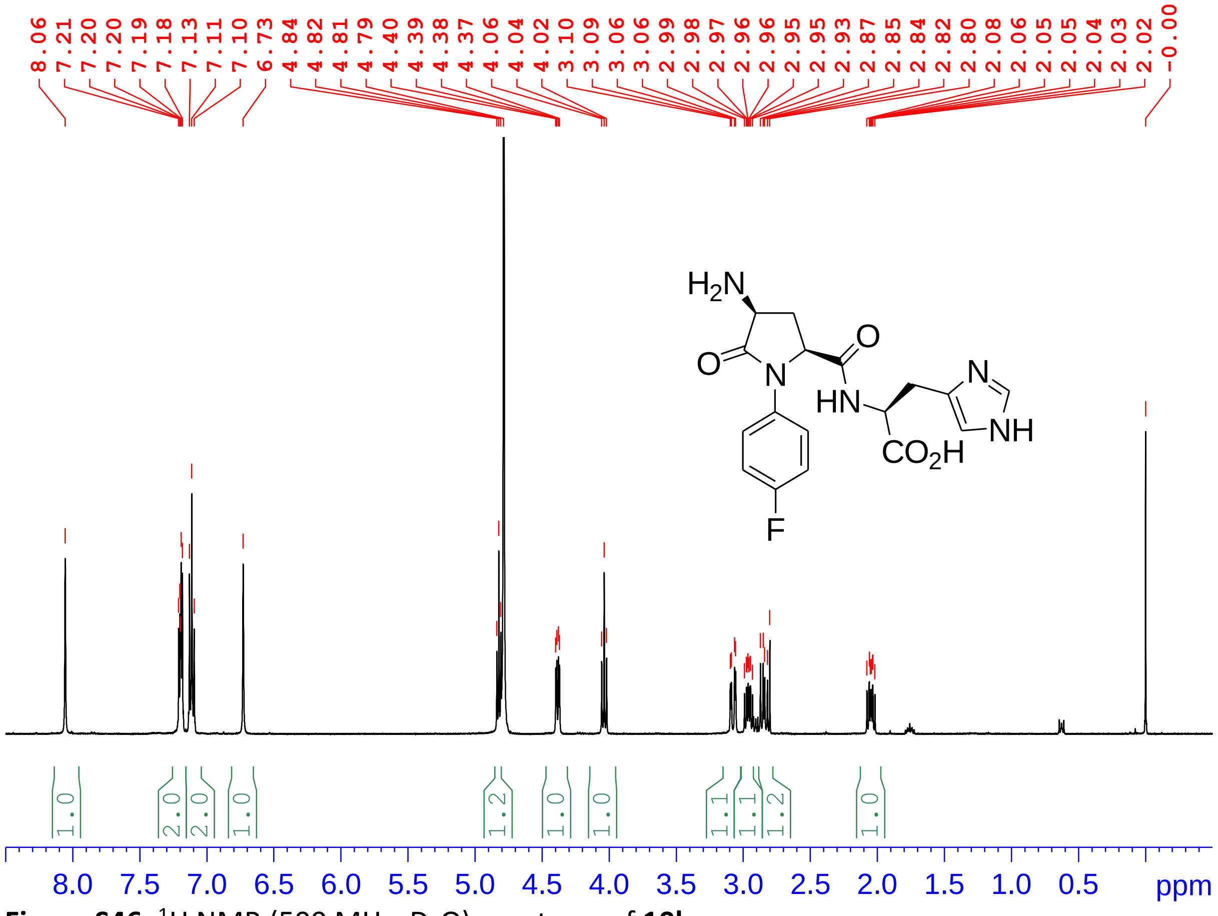

Figure S46. ^{1}H NMR (500 MHz, D_2O) spectrum of **10b**.

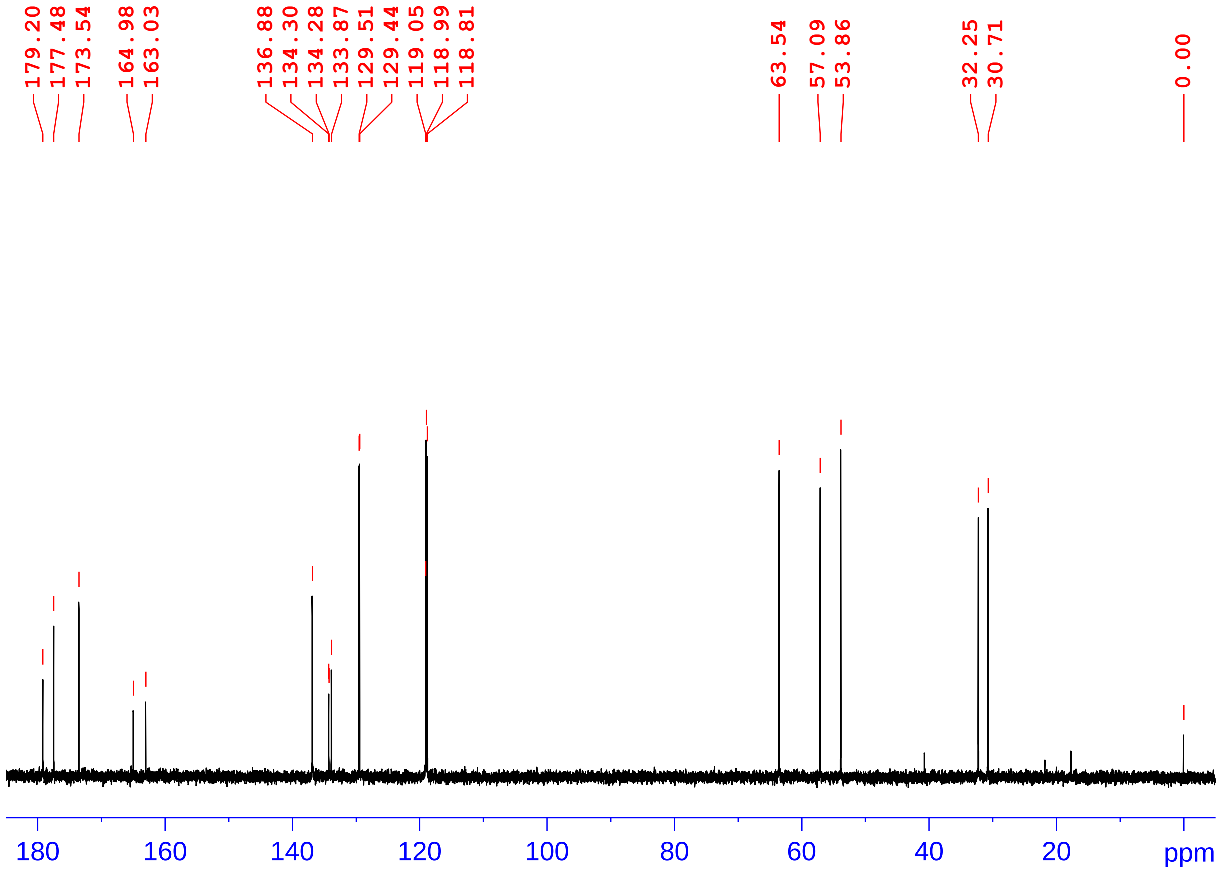

Figure S47. ^{13}C NMR (126 MHz, D_2O) spectrum of **10b**.

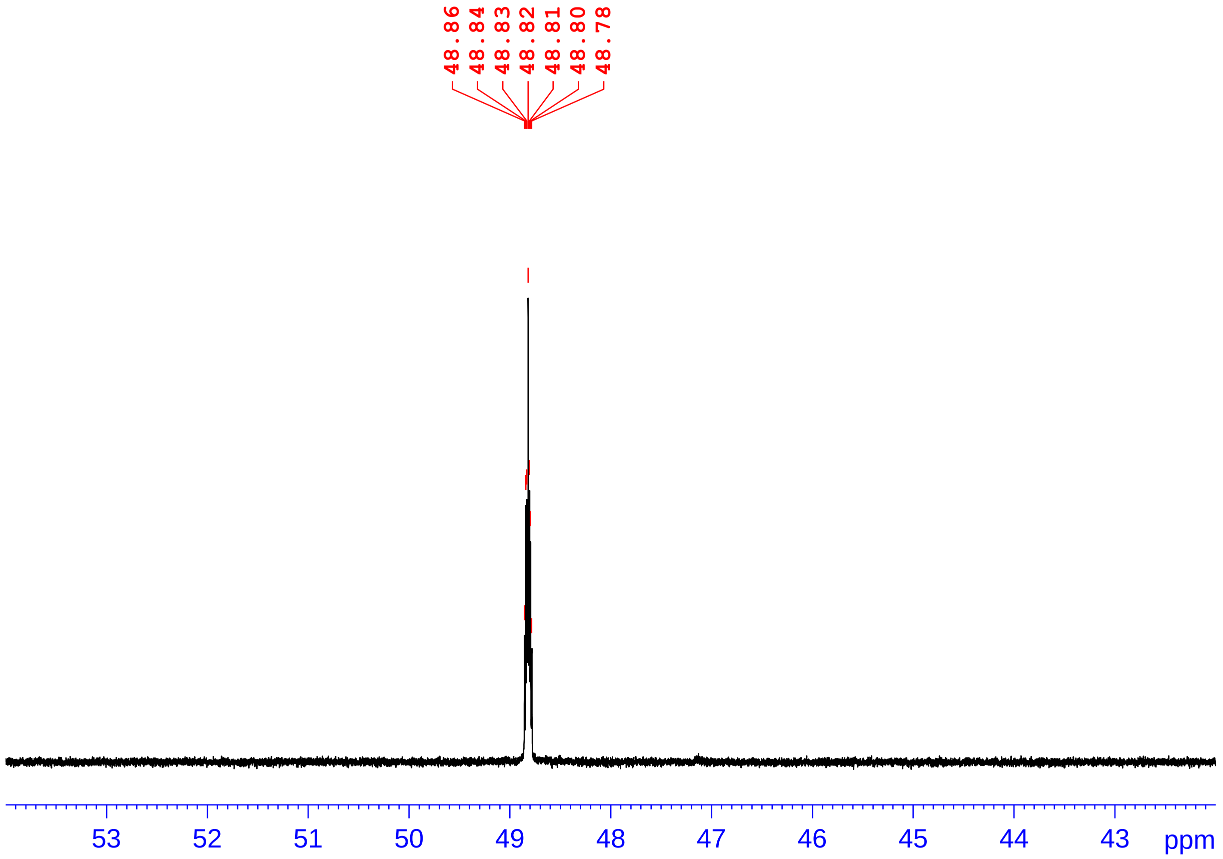

Figure S48. ^{19}F NMR (376 MHz, D_2O) spectrum of **10b**.

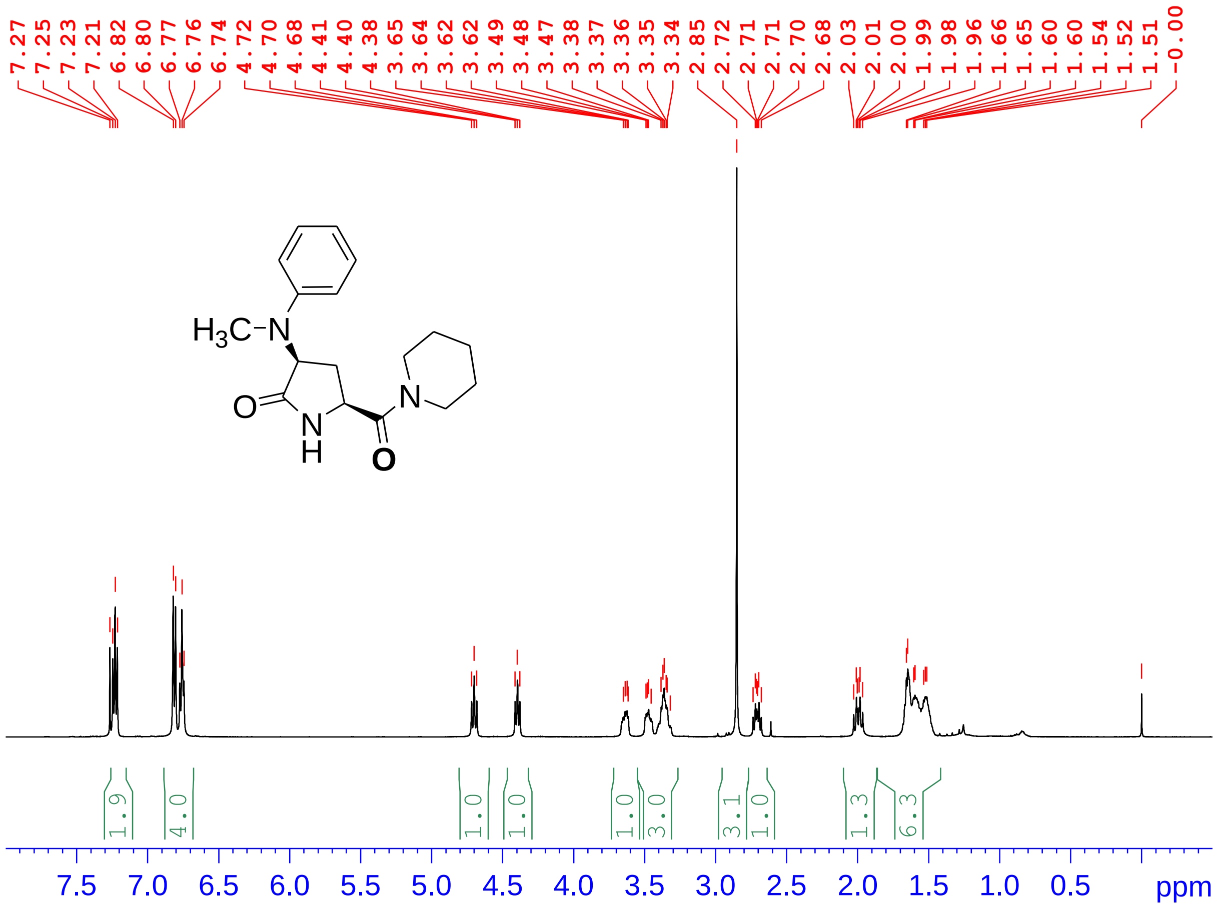

Figure S49. ^{1}H NMR (500 MHz, $CDCl_3$) spectrum of **12**.

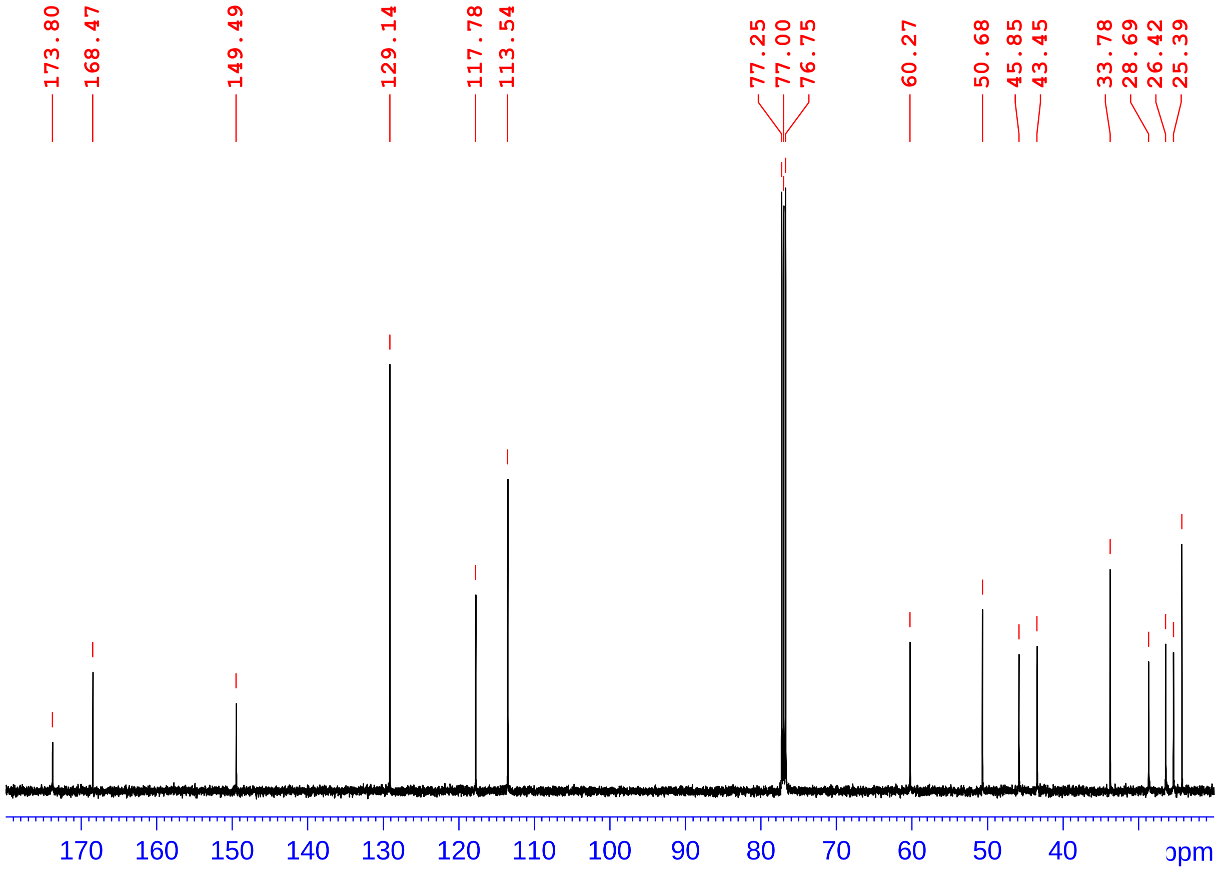

Figure S50. ^{13}C NMR (126 MHz, $CDCl_3$) spectrum of **12**.

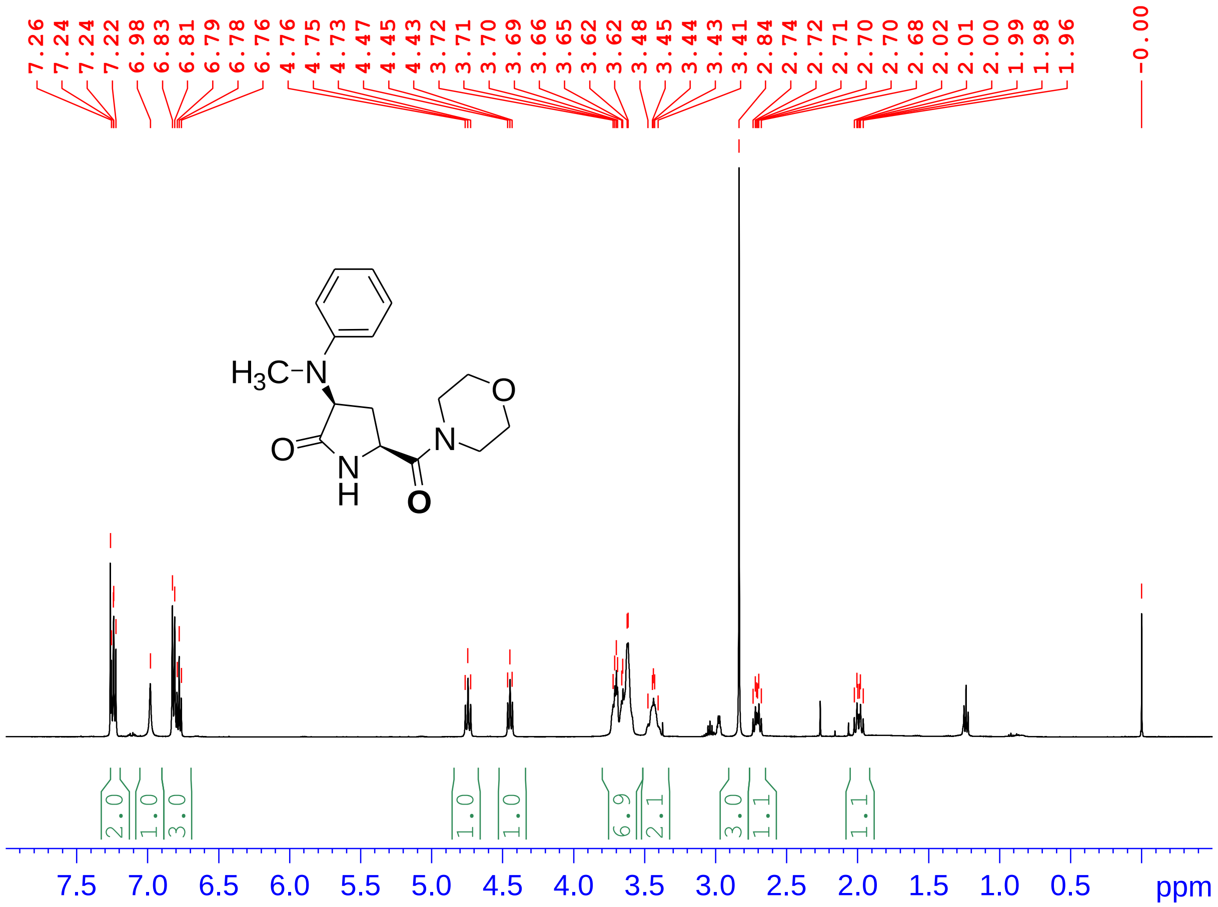

Figure S51. ^{1}H NMR (500 MHz, $CDCl_3$) spectrum of **13**.

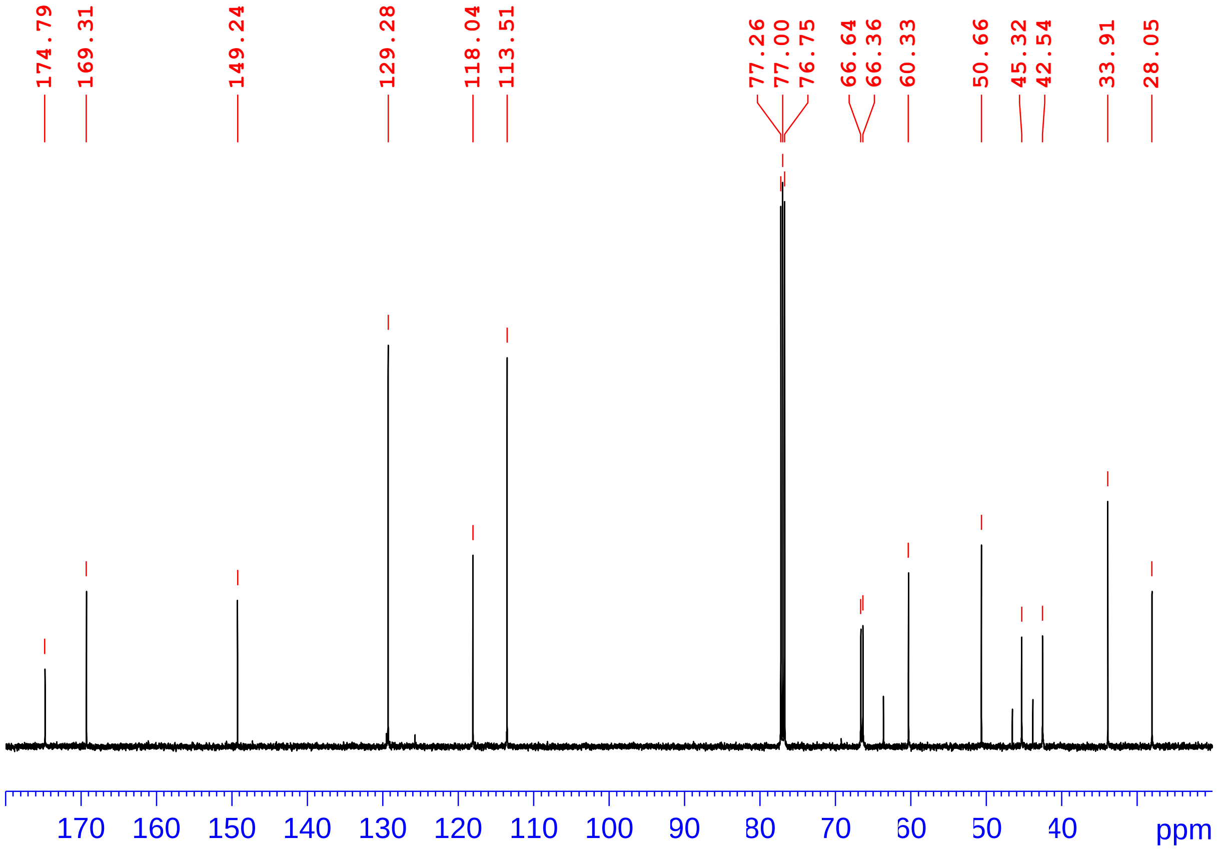

Figure S52. ^{13}C NMR (126 MHz, $CDCl_3$) spectrum of **13**.

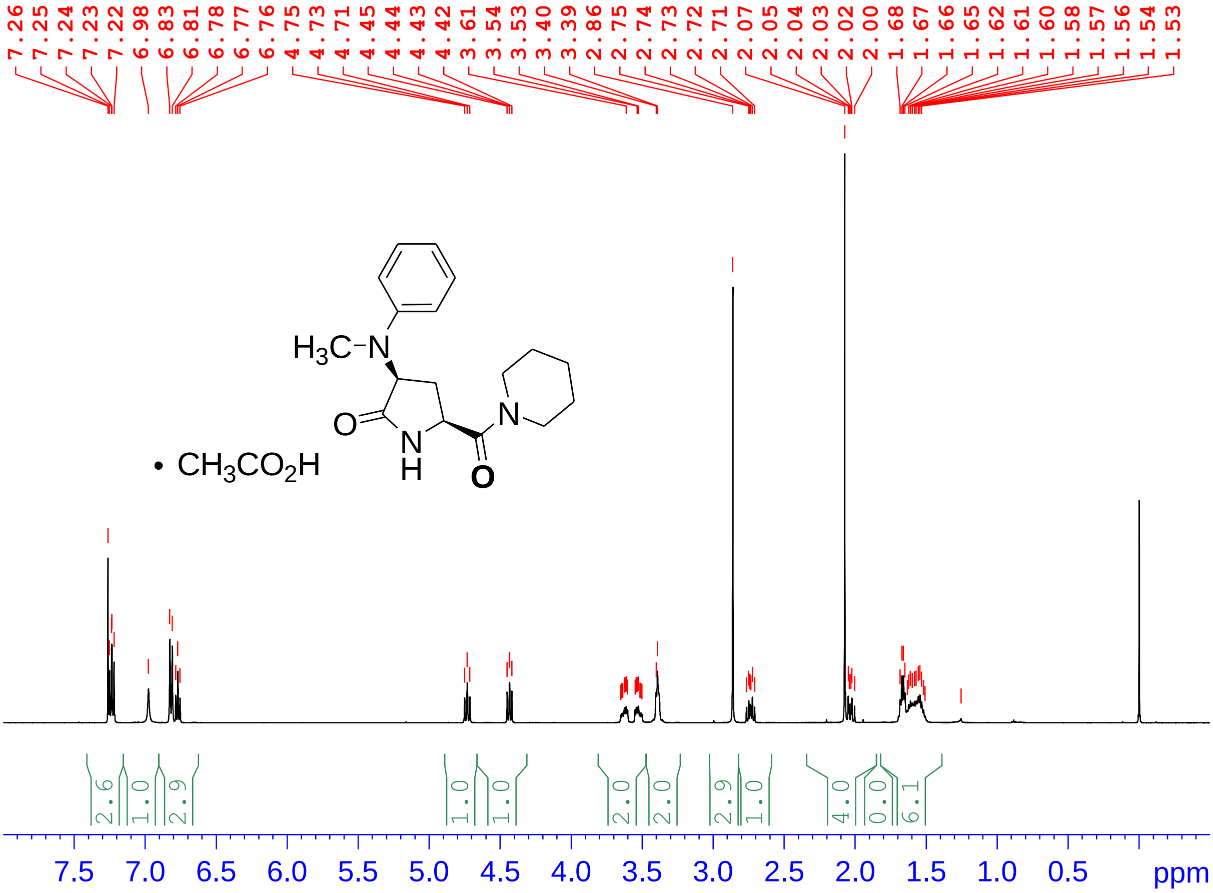

Figure S53. ^{1}H NMR (500 MHz, $CDCl_3$) spectrum of **14**.

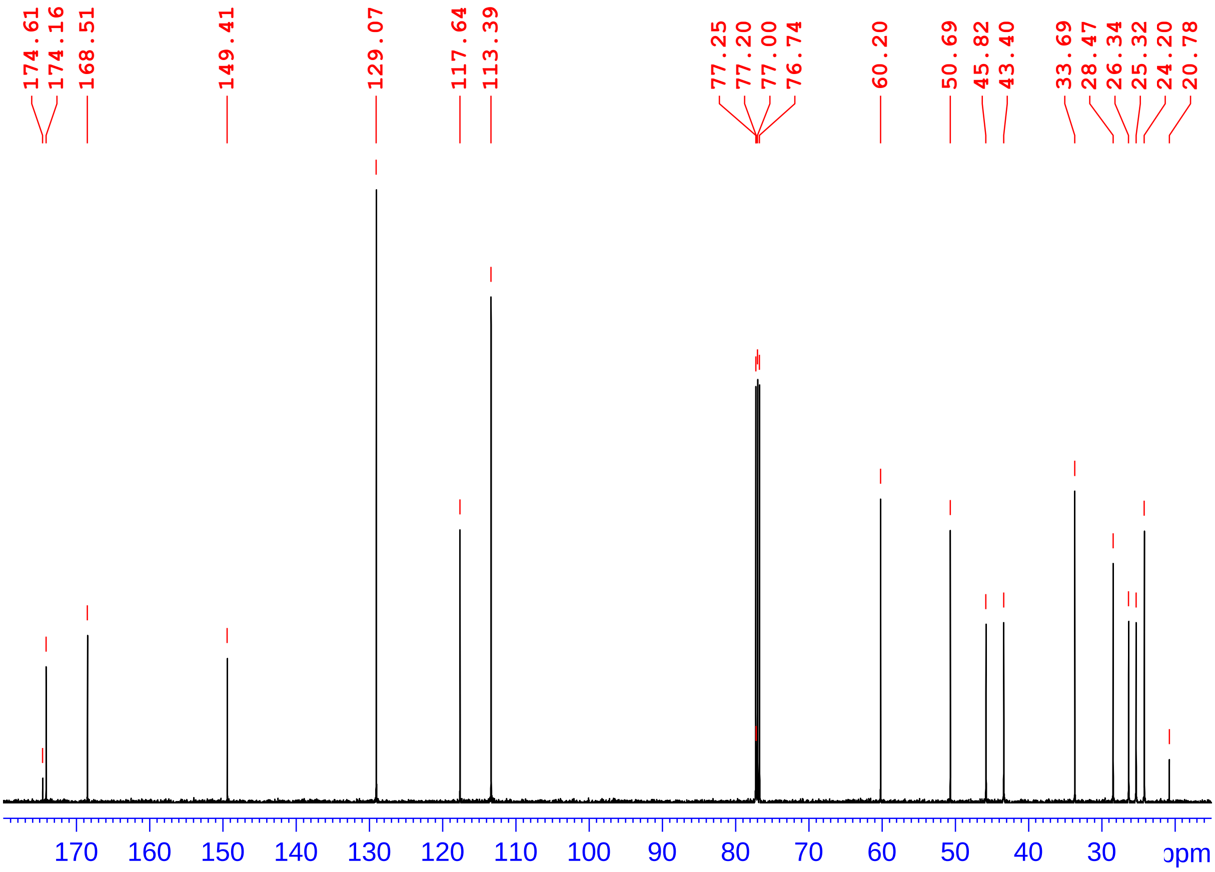

Figure S54. ^{13}C NMR (126 MHz, $CDCl_3$) spectrum of **14**.

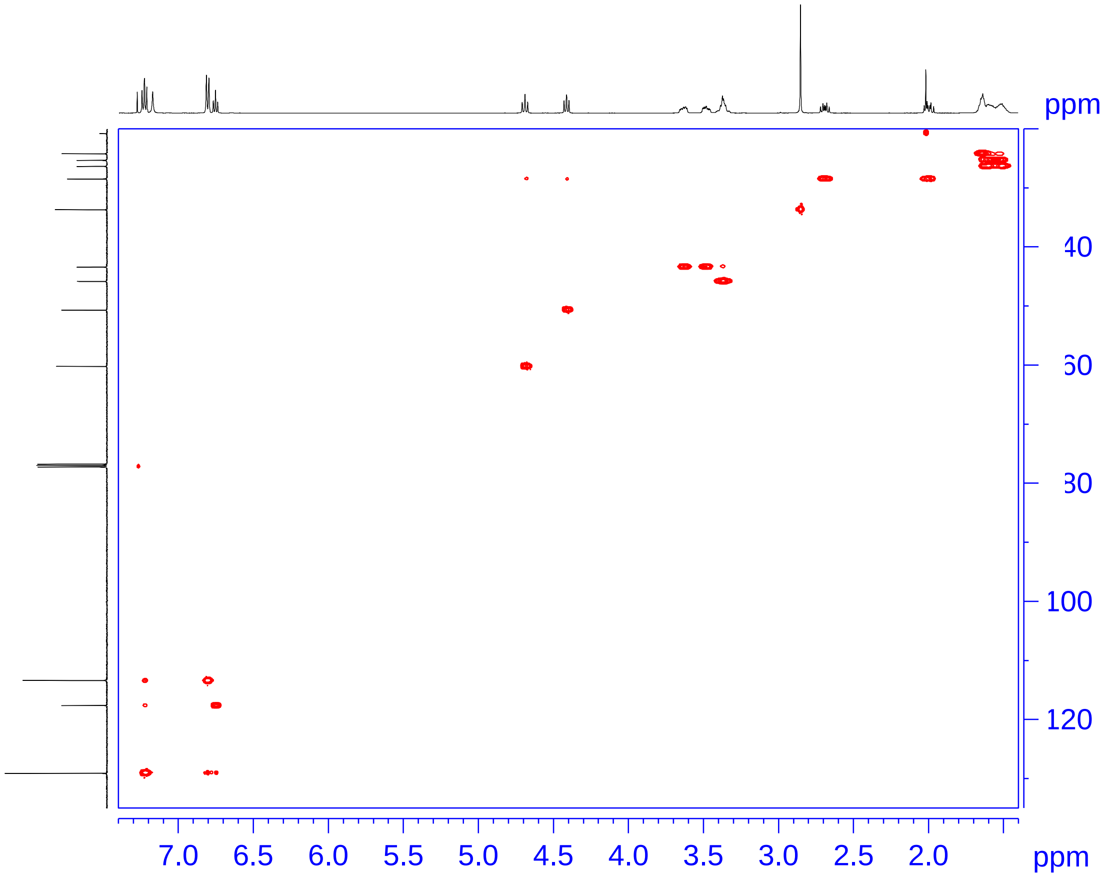

Figure S55. 2D ^{1}H-^{13}C HSQC (500 MHz, $CDCl_3$) spectrum of **14**.

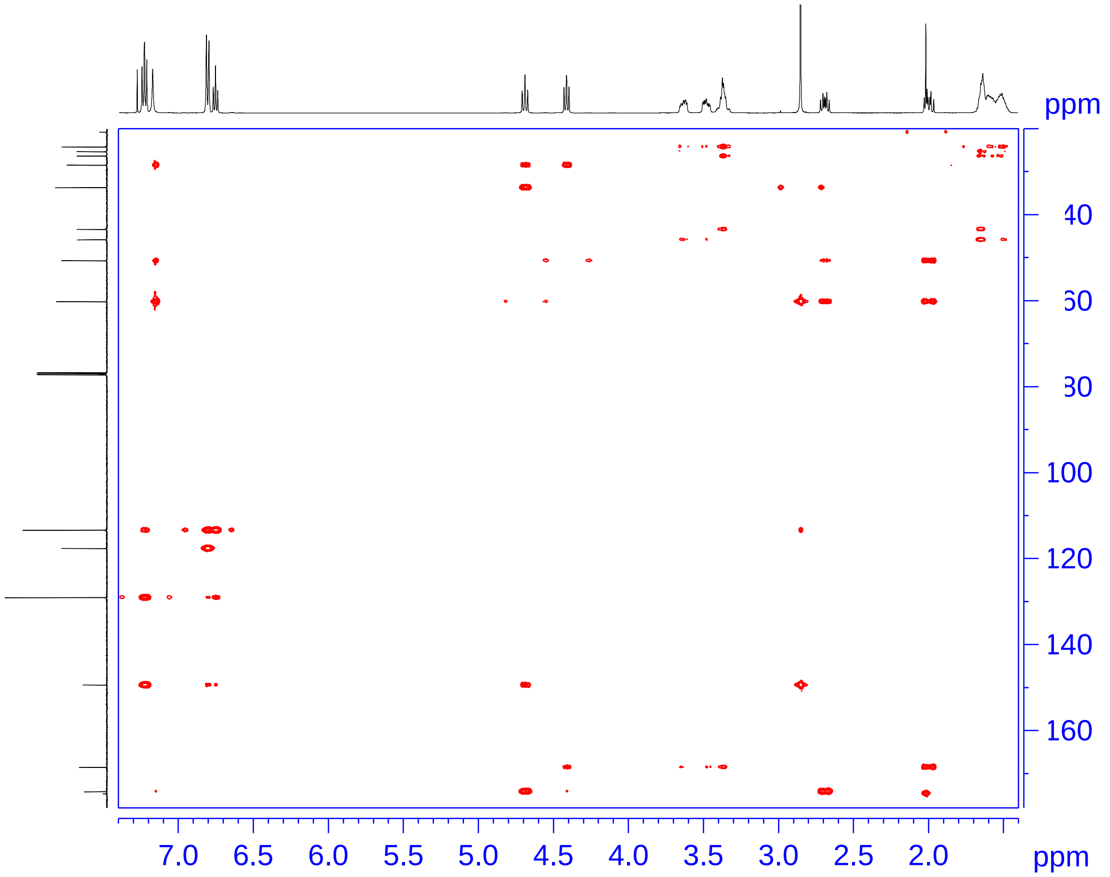

Figure S56. 2D ^{1}H-^{13}C HMBC (500 MHz, $CDCl_3$) spectrum of **14**.

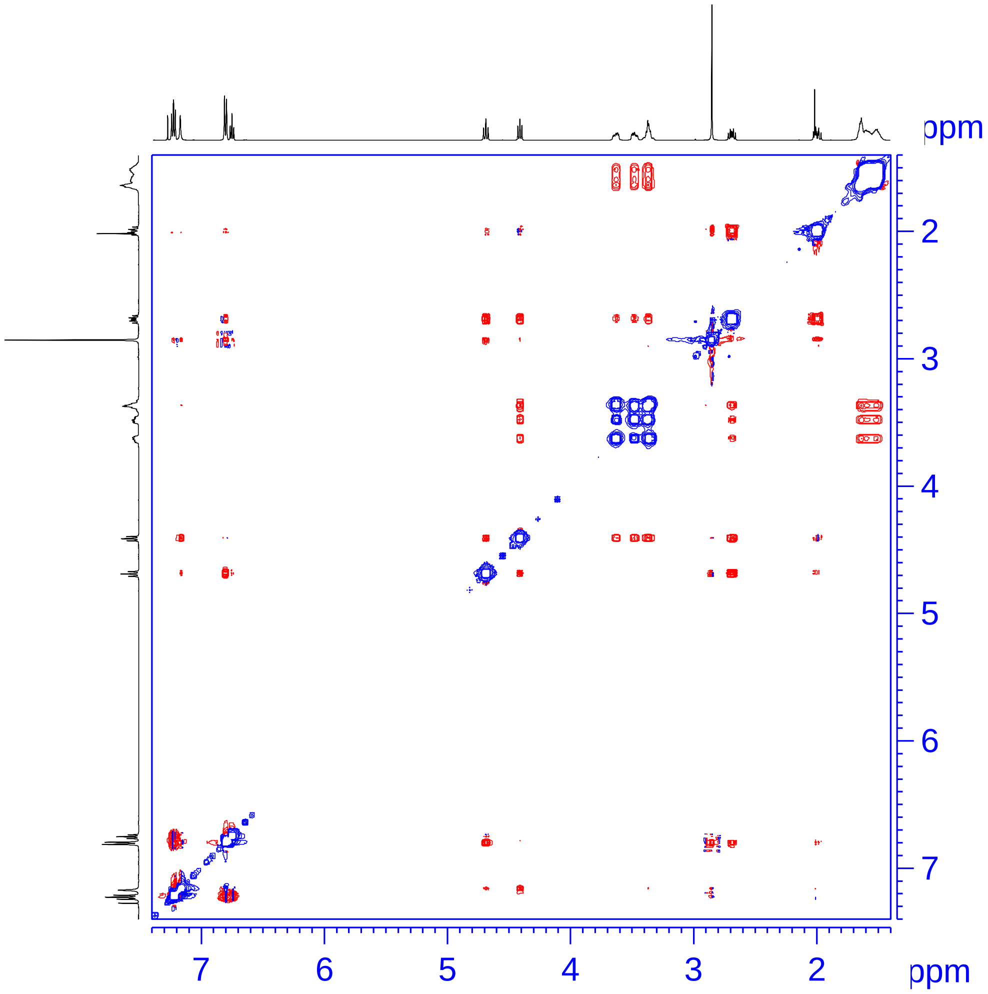

Figure S57. 2D ^{1}H-^{13}C NOESY (500 MHz, $CDCl_3$) spectrum of **14**.

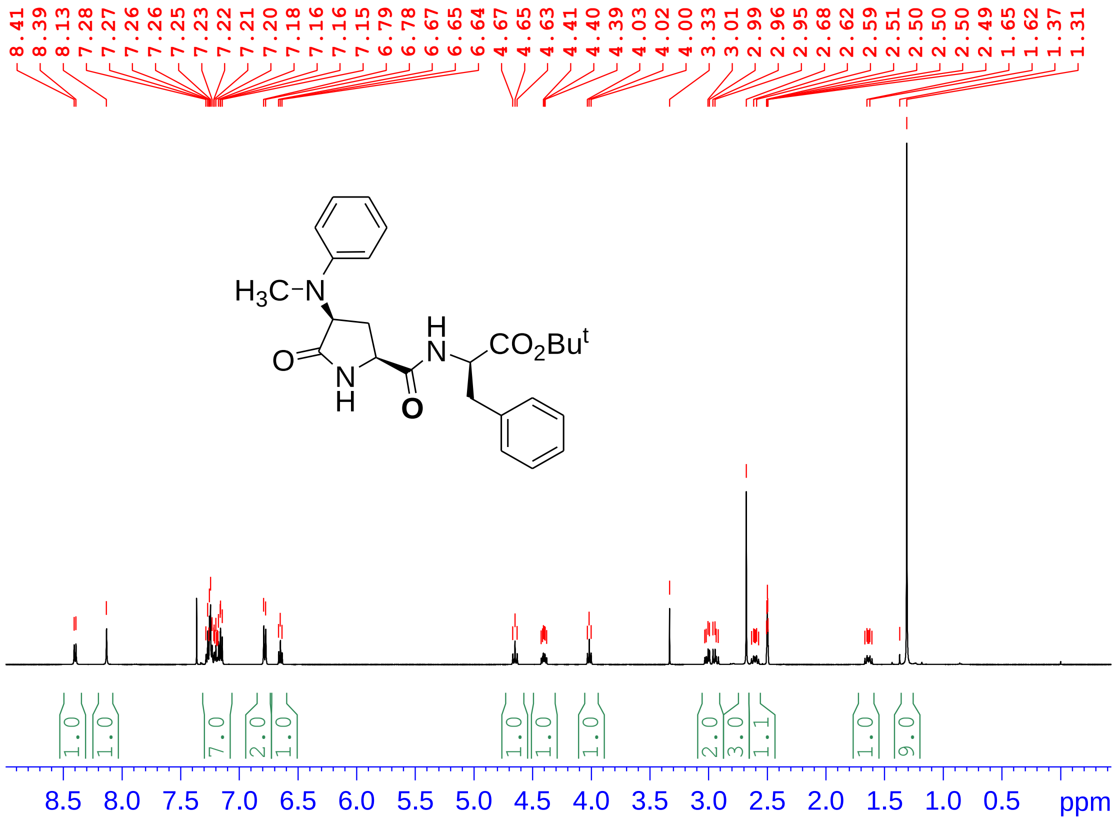

Figure S58. ^{1}H NMR (500 MHz, DMSO-d_6) spectrum of **15**.

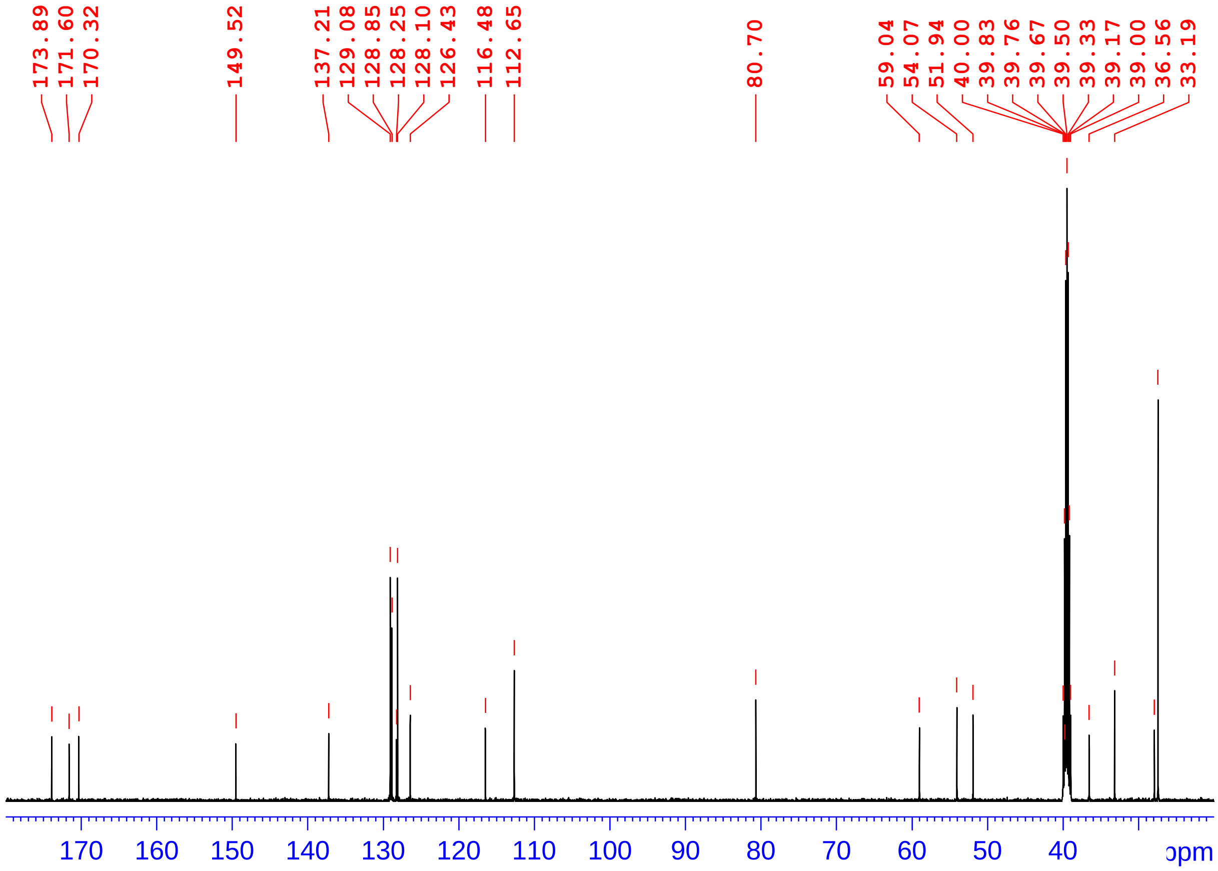

Figure S59. ^{13}C NMR (126 MHz, DMSO-d_6) spectrum of **15**.

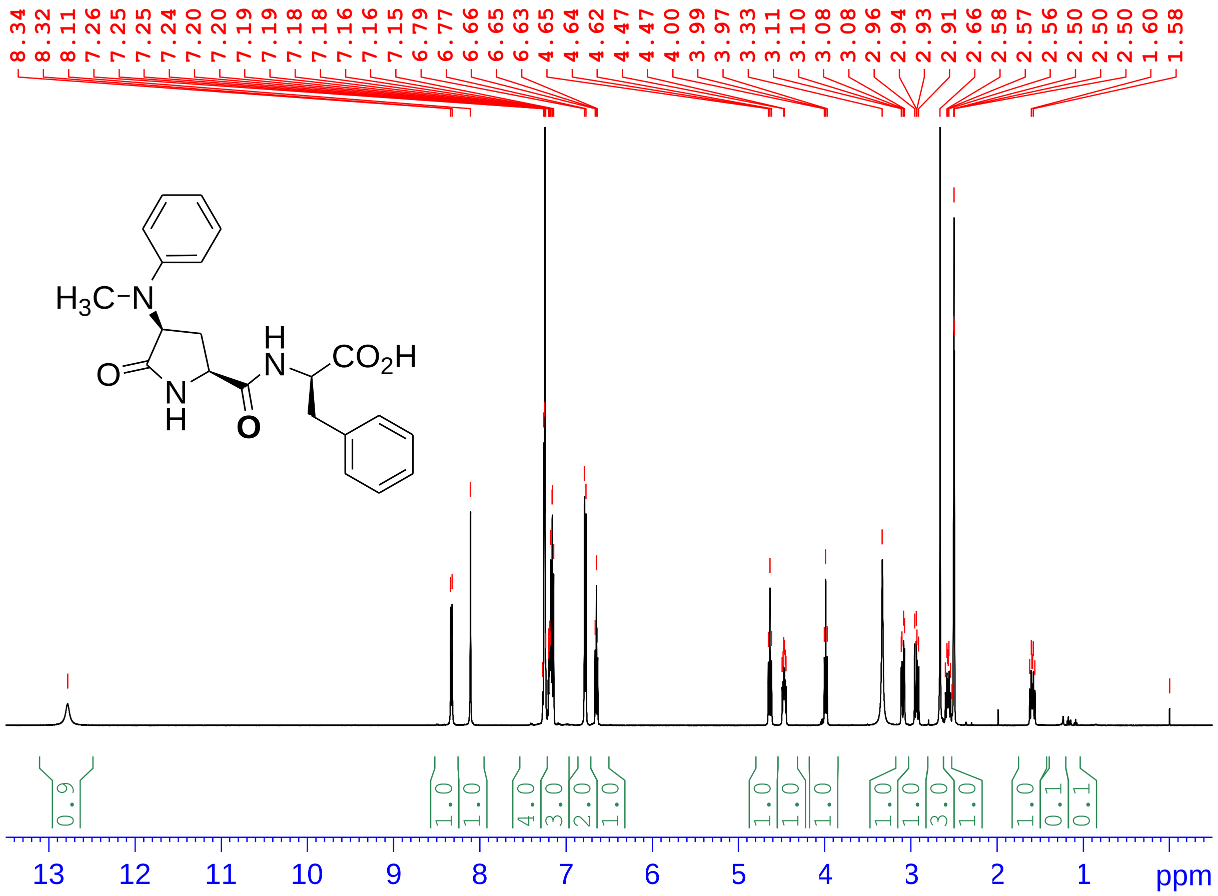

Figure S60. ^{1}H NMR (500 MHz, DMSO-d_6) spectrum of **16**.

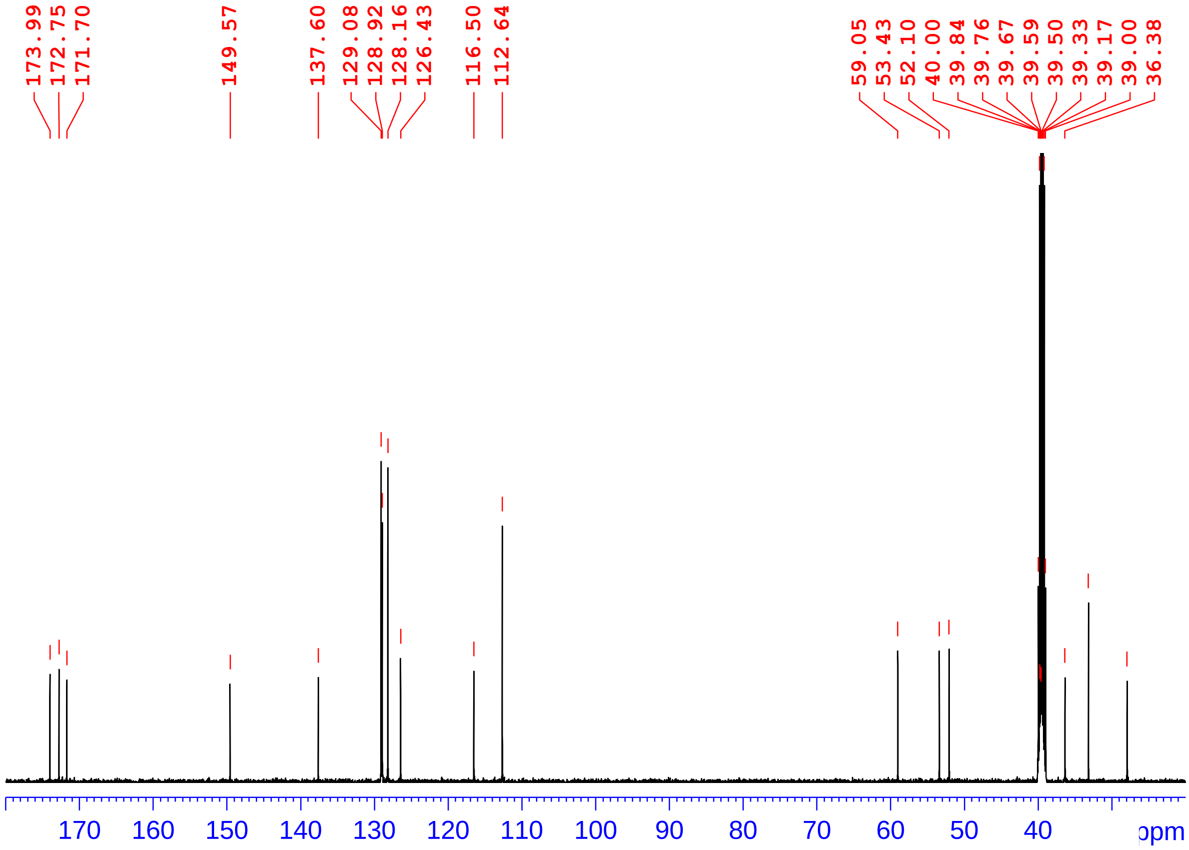

Figure S61. ^{13}C NMR (126 MHz, DMSO-d_6) spectrum of **16**.

X-Ray Diffraction Data

Table S1. Selected X-ray single-crystal data and structure refinement details of compound **14**

Compound	**14**
CCDC no.	**2290217**
Molecular formula	$C_{19}H_{27}N_3O_4$
Molecular weight	361.43
Crystal description	Colorless planks
Solvent for crystallization	*n*-hexane–EtOAc, 10 : 1
Radiation type	Mo-*K*α
T, K	295(2)
Crystal system	orthorhombic
Space group	$P2_12_12_1$
a, Å	5.7515(6)
b, Å	16.924(2)
c, Å	19.7410(18)
α, deg.	90.00
β, deg.	90.00
γ, deg.	90.00
V, Å^3	1921.6(4)
Z	4
$\rho_{calc.}$, g·cm^{-3}	1.249
μ, g·cm^{-3}	0.088
θ_{min} / θ_{max}, deg.	3.747/22.483
Independent reflections	6650
R_{int}	0.0589
Reflections with $I > 2\sigma(I)$	4446
Completeness (%) (for θ, deg.)	99.00 (28.00)
S on F^2	0.989
$R_1[I > 2\sigma(I)]$	0.0765
$wR_2[I > 2\sigma(I)]$	0.1656
R_1 (all reflections)	0.1450
wR_2 (all reflections)	0.2273
$\Delta\rho_{max}$ / $\Delta\rho_{min}$, e·Å^{-3}	0.17/–0.27
